# Multiphosphorylation-Dependent Recognition of Anti-pS2 Antibodies against RNA Polymerase II C-Terminal Domain Revealed by Chemical Synthesis

**DOI:** 10.1021/jacs.4c01902

**Published:** 2024-04-19

**Authors:** Emanuele Piemontese, Alina Herfort, Yulia Perevedentseva, Heiko M. Möller, Oliver Seitz

**Affiliations:** † Institut für Chemie, Humboldt-Universität zu Berlin, Brook-Taylor-Straße 2, 12489 Berlin, Germany; ‡ Institut für Chemie, Universität Potsdam, Karl-Liebknecht-Straße 24-25, 14476 Golm, Germany

## Abstract

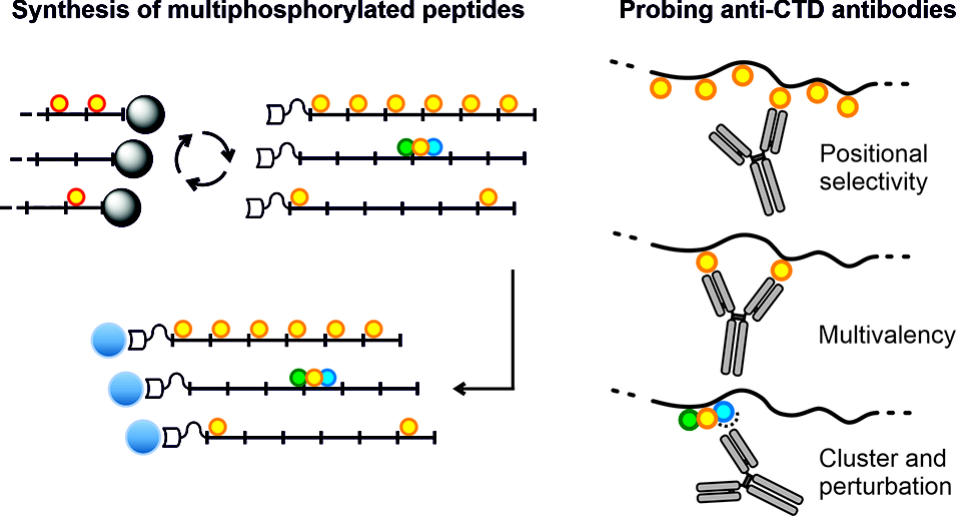

Phosphorylation is a major constituent of the CTD code, which describes the set of post-translational modifications on 52 repeats of a YSPTSPS consensus heptad that orchestrates the binding of regulatory proteins to the C-terminal domain (CTD) of RNA polymerase II. Phospho-specific antibodies are used to detect CTD phosphorylation patterns. However, their recognition repertoire is underexplored due to limitations in the synthesis of long multiphosphorylated peptides. Herein, we describe the development of a synthesis strategy that provides access to multiphosphorylated CTD peptides in high purity without HPLC purification for immobilization onto microtiter plates. Native chemical ligation was used to assemble 12 heptad repeats in various phosphoforms. The synthesis of >60 CTD peptides, 48–90 amino acids in length and containing up to 6 phosphosites, enabled a detailed and rapid analysis of the binding characteristics of different anti-pSer2 antibodies. The three antibodies tested showed positional selectivity with marked differences in the affinity of the antibodies for pSer2-containing peptides. Furthermore, the length of the phosphopeptides allowed a systematic analysis of the multivalent chelate-type interactions. The absence of multivalency-induced binding enhancements is probably due to the high flexibility of the CTD scaffold. The effect of clustered phosphorylation proved to be more complex. Recognition of pSer2 by anti-pSer2-antibodies can be prevented and, perhaps surprisingly, enhanced by the phosphorylation of “bystander” amino acids in the vicinity. The results have relevance for functional analysis of the CTD in cell biological experiments.

## Introduction

Phosphorylation of specific amino acid side chains is one of nature’s most important means of modulating the structure and function of proteins. Understanding the biological consequences of protein phosphorylation requires precise control over the phosphorylation events. Chemical synthesis provides a powerful tool to investigate and manipulate protein phosphorylation in a controlled and systematic manner.^1^ One of the key advantages of chemical synthesis is the ability to create phosphopeptides and phosphoproteins with site-specific phosphorylation.^2−7^ This level of control is crucial because phosphorylation often occurs at multiple sites within a protein, and each phosphorylation event can have distinct functional consequences.^8−17^

**Figure 1:**
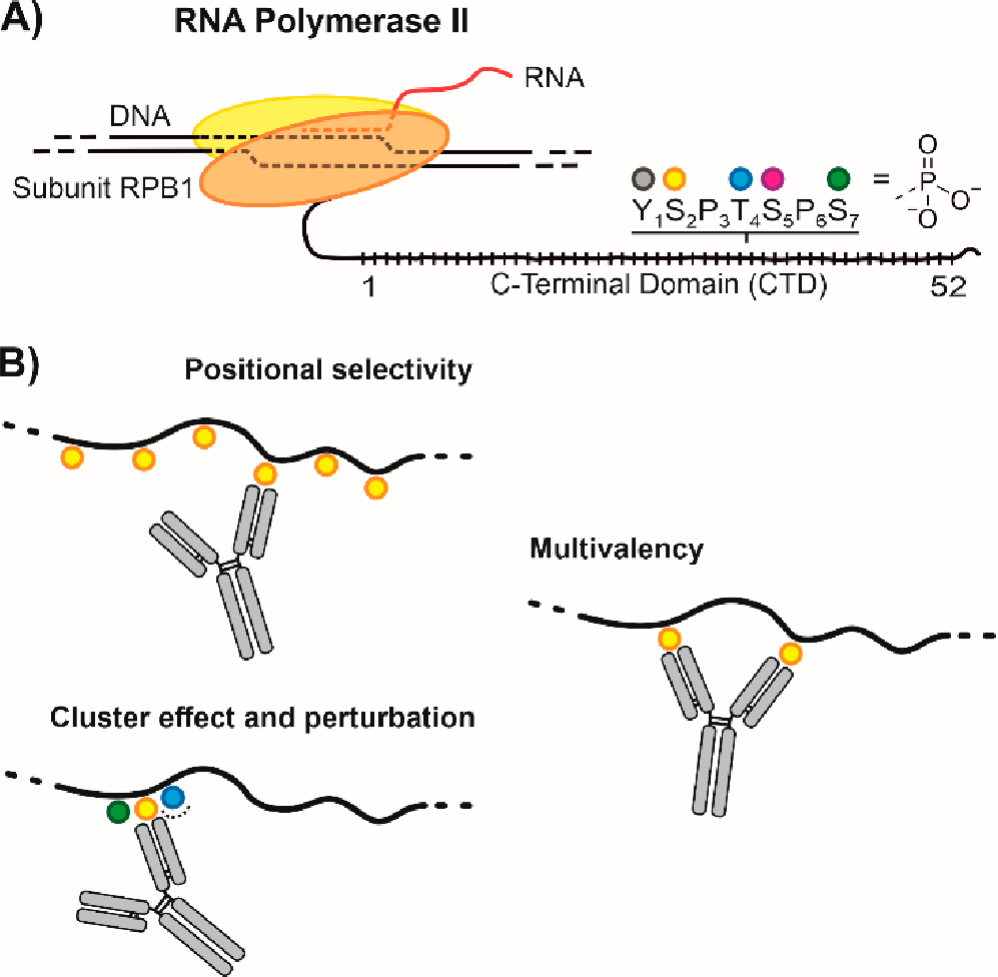
(A) The C-terminal domain (CTD) of subunit RBP1 of RNA polymerase II comprises multiple repeats of a YSPTSPS consensus motif. Phosphorylation of Y1, S2, T4, S5, and S7 and other post-translational modifications contribute to the CTD code recognized by regulatory proteins. (B) Antibodies are important tools for CTD analysis. Chemical synthesis of long, multiply phosphorylated CTD peptides can provide information on whether antibody binding is position-specific and enhanced by multivalent interactions and the effects of adjacent second and third phosphorylation. In this study, the focus is on pSer and pThr.

A prime example of multiphosphorylation is found at the C-terminal domain (CTD) of the large subunit of RNA polymerase II. In humans, the CTD is composed of 52 repeats of the consensus heptad −YSPTSPS– (Figure 1A). Any amino acid (but Pro) can exist in phosphorylated form.^18,19^ The domain acts as a landing hub,^20^ and post-translational modifications control the recruitment of proteins regulating transcription, RNA splicing, and chromatin remodeling.^21^ CTD phosphorylation changes dynamically during transcription^22^ and all the possible modification states form the so-called CTD code.^19^ Functional analysis of the CTD modifications relies on monoclonal phospho-specific antibodies used, for example, in chromatin immunoprecipitation assays (ChIP).^23−27^ Relative to their frequent usage, the specificity of these key tools and potential interferences by bystander modifications have been little studied (Figure 1B).^26,28−32^ Antibodies are bivalent, and in principle, bivalency-enhanced interactions could occur when two phosphosites are presented at a suitable distance. Very strong binding of an antibody to only two phosphorus residues, when suitably positioned, may be mistaken for excessive CTD phosphorylation. Previous studies used phosphopeptides that span two to four heptad repeats. However, given the large distance between the two antigen binding sites, relatively long phosphopeptides need to be synthesized, which are out of reach of linear solid-phase phosphopeptide synthesis. Moreover, it often remains unclear how the recognition of phospho-specific anti-CTD antibodies is affected by second or even third phosphorylation events in neighboring heptads. One possible reason for this could be the difficulty in synthesizing multiphosphorylated peptides.^33^ It is therefore difficult to estimate under which conditions a specific antiphospho-CTD antibody will give false negative or false positive results. Without knowledge of the binding repertoire, uncertainties remain in the interpretation of antibody-based assays, and possibilities for targeted control tests remain unexplored.

Our goal was to develop a method that provides facile and reliable access to multiphosphorylated CTD peptides of up to 12 heptad repeats (84 amino acids) in length. As a key to rapid screening, we eliminated the need for HPLC purification. Given the difficulties in the synthesis of multiphosphopeptides, which have been widely reported,^12,34,35^ this represents a particular challenge. We also considered it useful to synthesize the phosphopeptides in a form that would allow quality control in solution and immobilization on microtiter plates, such that a library of possible phosphoforms can be analyzed by indirect ELISA. Herein, we report the HPLC-free synthesis of six and 12 heptad repeat long CTD peptides bearing up to six phospho residues. This allowed us to evaluate the binding characteristics of three commercially available monoclonal antibodies (mAbs) from different vendors that are pS2-specific. With a library of 60 phosphopeptides, we tested positional selectivities, cross-reactivities, perturbation by second and third phosphorylation, and multivalency-enhanced interactions. The data point to previously unknown features of antibody–CTD interactions.

## Results

### Synthesis of Multiphosphorylated Peptides

Among the CTD modifications, phosphorylation at serine and threonine has been the most extensively studied. We therefore focused our investigations on the development of a synthesis method providing access to long serine/threonine-phosphorylated CTD peptides. In solid-phase synthesis, phosphoserine/-threonine is commonly introduced using N-Fmoc-protected building blocks bearing a single benzyl group to protect the phosphomonoester. This minimizes β-elimination at dibenzyl-protected phosphoserine/threonine, a typical side reaction occurring during treatment with piperidine used for Fmoc cleavage.^36^ However, with monobenzylated building blocks, piperidine remains in the form of a piperidinium benzylphosphate. In the coupling step, the piperidine can react with the activated carboxylic acid, theoretically consuming one equivalent of reagent per phosphorylation. This, the bulkiness of the building blocks, and electrostatic repulsions have been considered to be the main factors complicating the synthesis of multiphosphorylated peptides.^1^

**Figure 2:**
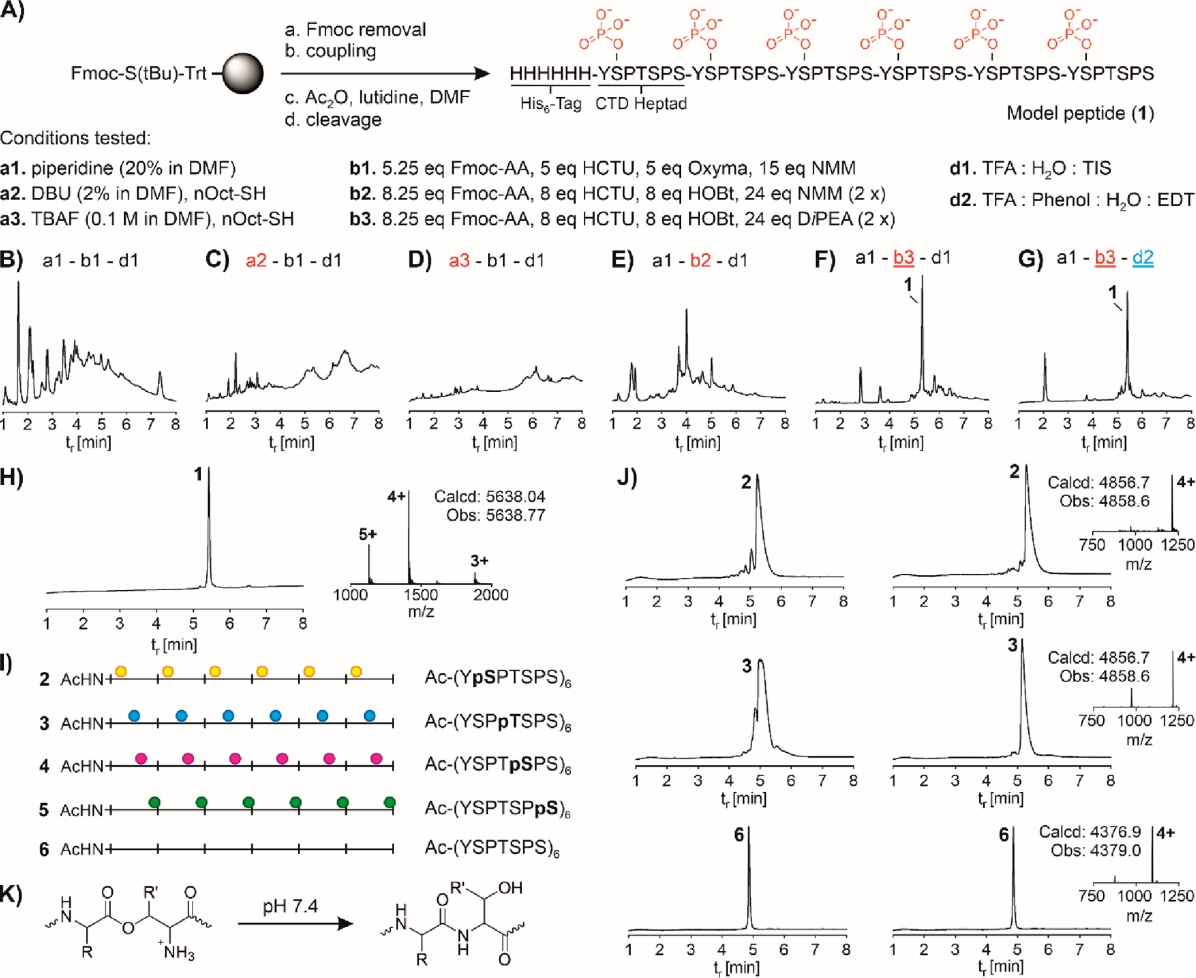
Synthesis of the hexaphosphorylated CTD peptides. (A) Optimization of linear solid-phase synthesis of a His_6_-tagged hexaheptad peptide (**1**) phosphorylated at each Ser2 residue. (B–G) UPLC analysis of crude peptides obtained after variation of conditions for Fmoc removal (a1–a3), coupling (b1–b3), and TFA cleavage (d1, d2). Variations of the initially used protocol (a1, b1, d1) are highlighted in color. For further details on the synthesis method, see SI paragraph 4.1. (H) UPLC-HRMS analysis of HPLC-purified peptide **1**. (I) Schematic depiction and sequence of hexaheptad peptide phosphorylated at each of Ser2 (**2**), Thr4 (**3**), Ser5 (**4**), and Ser7 (**5**) and nonphosphorylated peptide **6**. (J) UPLC-MS analysis of HPLC-purified and lyophilized hexaheptad peptides dissolved in H_2_O (left) or 200 mM NaH_2_PO_4_ buffer at pH 7.4 (right). (K) O → N acyl shift reverses depsipeptide formation.

For an optimization of *O*-phosphopeptide synthesis, we targeted the six heptad repeats long peptide **1**, bearing six phosphorylations at Ser2 of each heptad (Figure 2A). The peptide further contained an N-terminal hexahistidine tail, which was deemed to be useful for affinity purification and immobilization on microtiter plates. Solid-phase synthesis was performed in a 25 μmol scale on Trt-TentaGel resin using open-column reactors and a parallel peptide synthesizer. First, we used 5.25 equiv of each amino acid building block in coupling reactions including 5 equiv of HCTU, NMM as base, and Oxyma as additive. NMM was chosen instead of DIPEA with the aim to minimize liberation of piperidine from the piperidinium benzylphosphate and, hence, decrease the unproductive consumption of building blocks. Furthermore, we speculated that the use of NMM could help reduce β-elimination and racemization during coupling. HPLC analysis of material obtained after TFA cleavage did not contain the full-length peptide, but a series of truncation sequences (Figure 2B). Recently, DBU was used as a replacement of piperidine to reduce the extent of β-elimination during Fmoc cleavage at elevated temperature.^37,38^ However, synthesis of the hexaphosphopeptide **1** still failed (Figure 2C) when we replaced 20% piperidine in DMF with 2% DBU in DMF containing octanethiol as scavenger of dibenzofulvene formed during Fmoc cleavage. Reducing the concentration of DBU and avoiding octanethiol provided no improvement (data not shown). Inspired by work from Ueki et al.,^39^ we used 0.1 M TBAF in the Fmoc cleavage step, however, without success (Figure 2D). Based on the unsuccessful attempts to optimize Fmoc cleavage, we concluded that the problems occurred elsewhere. Next, we increased the equivalents of the reagents, employed double coupling, and evaluated the influence of the coupling additive and the base, keeping the uronium-based HCTU as activator.^40^ While the higher amount of reagents and the single substitution of Oxyma with HOBt did not provide the full product (Figure 2E), replacing the weak base NMM with DIPEA also proved beneficial. The HPLC trace of the crude product (Figure 2F) now revealed a major peak showing the *m*/*z* ratio expected for hexaphosphopeptide **1**. However, UPLC-MS analysis revealed peaks showing *m*/*z* ratios corresponding to benzyl- and *tert*-butyl adducts (M + 90 and M + 56). We inferred that water and triisopropylsilane (TIS) used as scavengers in TFA cleavage solutions were not strong enough to prevent the alkylation of the tyrosine residues. The testing of TFA cleavage cocktails showed that the addition of 1,2-ethanedithiole (EDT) and phenol to the cleavage cocktail was beneficial to reduce alkylation (Figure 2G).

Microwave heating has been reported to improve yields of peptides containing up to three phosphorylations.^41,42^ Application of a published protocol,^42^ which involved coupling of 5 equiv of amino acid at 72–75 °C with activation by 4.5 equiv of HBTU and 10 equiv of DIPEA, resulted in a mixture of compounds (Figure S1) containing minor amounts of the desired hexaphosphopeptide. A thorough analysis for the synthesis of multiphosphorylated peptides has been provided by Samarasimhareddy et al.,^12^ who suggested to increase the excess as the number of incorporated phosphoamino acids increases. This dose escalation approach minimizes the number of excess equivalents. Considering that only nanomole amounts of CTD phosphopeptides are required for antibody binding assays, we felt little need to minimize amino acid consumption. Instead, robustness and purity without HPLC purification are priorities for screening campaigns, and we therefore opted to apply an 8-fold excess of amino acid building blocks throughout the entire synthesis. To prove the general applicability of the synthesis method, we prepared the hexaphosphopeptides lacking a His tag with phosphorylations on all the possible serine and threonine residues (**2**–**5**, Figure 2I). While assembly of heavily phosphorylated peptides was remarkably smooth with the optimized conditions, UPLC-MS analysis of HPLC-purified compounds showed side-peaks that had *m*/*z* values identical to those of the main peaks (Figure 2J, left). We considered the possibility of an N→O acyl shift reaction forming a depsipeptide bond (Figure 2K, left), which has been observed previously in the synthesis of phosphotyrosine-containing peptides.^43^ Under acidic conditions, such as in HPLC eluates, hydroxyl groups of serine and threonine can attack the α-carboxy group of the N-terminal neighbor. Previously it had been reported that an N→O acyl shift was triggered by phosphorylation at tyrosine.^43^ Also with the CTD peptides, the byproduct did not form in the absence of phosphorylation (Figure 2J, bottom left). Fortunately, higher product purities were obtained once the phosphopeptides were dissolved in pH 7.4 buffer (Figure 2J, right). Under these conditions, an O→N acyl shift is favored, and depsipeptides should disappear (Figure 2K). An alternative explanation suggests that side peaks with identical *m*/*z* ratio may result from *cis*–*trans* proline isomerization. However, the disappearance of the isomeric byproducts at neutral pH and the lack of side peaks for the unphosphorylated peptide contradict this explanation.

### Design and Preparation of the Library

Along with serine 5, phosphorylation at serine 2 is one of the most studied modifications of the CTD. A variety of anti-pS2-specific antibodies have been used to obtain insights into the CTD code by ChIP,^44−47^ Western blot,^48−50^ immunofluorescence,^51^ and immunostaining,^52^ among many others,^53−56^ and to detect CTD binders. We selected three different recombinant rabbit-generated IgG phospho-specific anti-pS2 antibodies: E1Z3G (Cell Signaling Technology), EPR18855 (Abcam), and 2G1 (Thermo Fisher/Invitrogen). In contrast to previous epitope mappings focusing on diheptapeptides, we assembled peptides containing 6 and 12 heptad repeats to enable positional scanning and meaningful studies of potential multivalency effects. Focusing on the phosphorylation of serine and threonine residues, hexaheptad peptides were synthesized on a parallel peptide synthesizer. After assembly of the CTD core, an amino acid PEG_6_ linker was coupled prior to chain elongation with six subsequent histidine residues. The His_6_ tag enabled the rapid and parallel purification of the SPPS crude products by affinity capture to Ni-NTA-functionalized agarose beads. Cleavage impurities and truncation sequences are washed out, and the products are, then, eluted using a solution of acetic acid 250 mM in water, which is easily removed upon lyophilization (Figure S7).

**Figure 3:**
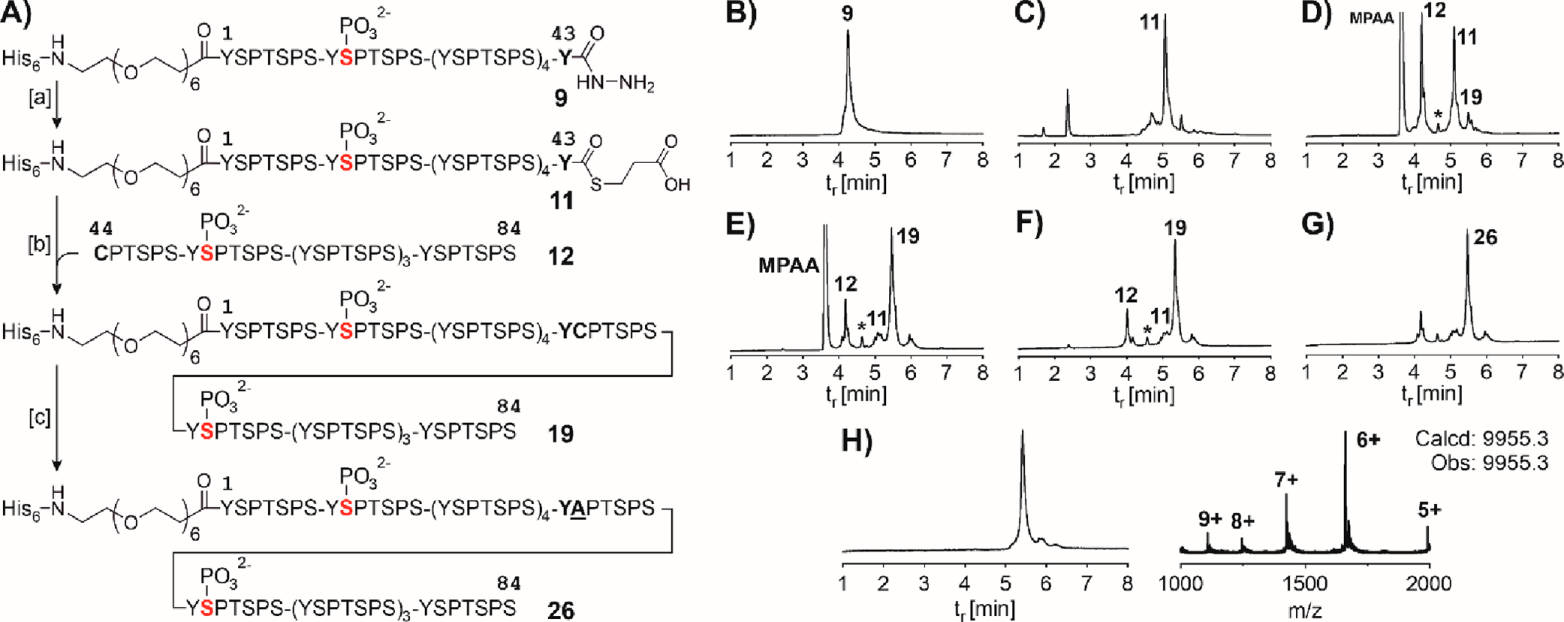
(A) Representative example of the synthesis of CTD peptides (**24**–**30**) composed of 12 heptad repeats by native chemical ligation. Conditions: [a] (i) 1 mM peptide, 15 equiv of AcAc, 200 equiv of MPA in buffer (6 M Gdn-HCl, pH 3), 30 min, room temp; (ii) pH 7.5, 1 h; [b] 10 mM N-terminal and 12.5 mM C-terminal fragment, 200 mM MPAA, 50 mM TCEP HCl in buffer (6 M Gnd-HCl, 200 mM Na_2_HPO_4_, pH 7), 4 h, room temp; [c] 1 mM crude peptide, 200 mM TCEP HCl, 100 mM NaBEt_4_ in buffer (6 M Gnd-HCl, 0.2 M sodium citrate pH 4.5), 5 min, room temp. UPLC analysis of (B) HPLC purified peptide hydrazide **9**, (C) after conversion to peptide thioester **11**, (D) native chemical ligation between **9** and **12** at *t* = 0 and (E) at *t* = 4 h, (F) after gel filtration of native chemical ligation mixture, and (G) after desulfurization. (H) UPLC-MS analysis of HPLC-purified 90-mer **26**. The * marks the peaks related to the hydrolysis side product.

For the preparation of phosphopeptides with a length of 12 heptad repeats, two peptide fragments of comparable size were used in native chemical ligation (NCL) at a Tyr-Cys junction, which was to be converted to a Tyr-Ala junction after ligation. The choice of the junction was made to prevent the hydrolysis of the thioester peptides. In fact, in the previously reported^57^ synthesis of peptide **7** by auxiliary-assisted chemical ligation, we chose the junction between two heptads (Ser-Tyr), and we experienced a significant hydrolysis rate of the thioester at the serine C-terminal site. The hydrolysis was avoided by changing the junction and separating the thioester formation and NCL steps. The N-terminal segment was synthesized as a peptide hydrazide in an unphosphorylated form (**8**) and with pSer2 (**9**) at the second heptad (Figure 3A). The C-terminal segments were prepared in different phosphoforms that differed by the position of pSer2 residues. After HPLC purification the peptide hydrazides (**8** and **9**) were converted to MPA thioesters (**10** and **11**) by using Dawson’s pyrazolate method (Figure 3C).^58^ Ligation of peptide thioesters **10** and **11** with a slight excess of cysteine peptides (**12**–**16**) proceeded smoothly (Figure 3D → E), providing the 90-mer peptides as the major component after 4 h. For desulfurization, we applied Li’s tetraethylborate method^59^ directly on the gel-filtered crude of the ligation step (Figure 3F). As reported, the conversion was completed after only 5 min (Figure 3G). The reaction proceeded without detriment to phosphoserine. The desired products **24**–**30** were obtained in 10–50% yield over three steps and preparative HPLC purification (Table 1).

**Table 1:**
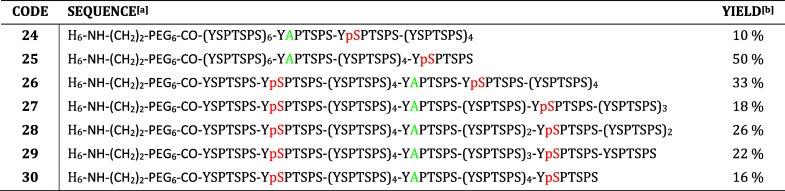
Dodecaheptad Phosphopeptides Are Accessed by Native Chemical Ligation and Ultrafast Desulfurization

a Letters in red mark the position of phosphorylation and in green the alanine mutations.

b Obtained after three-step synthesis shown in Figure 3.

### Antibody Binding to Monophosphorylated Peptides

**Figure 4:**
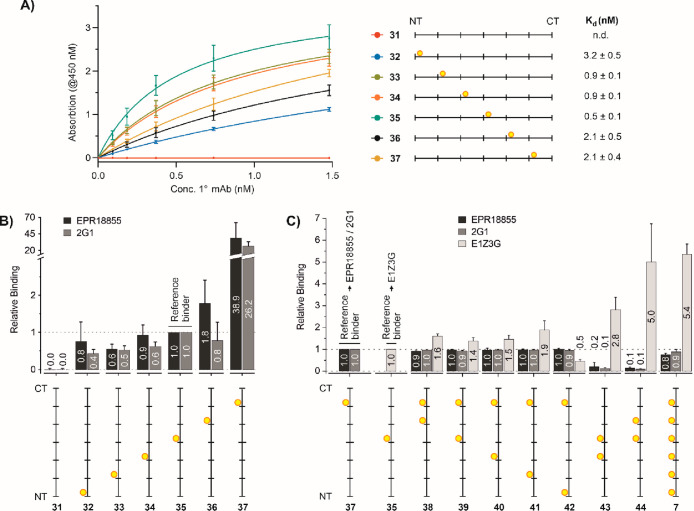
(A) Titration of the E1Z3G primary antibody to immobilized hexaheptade peptides. (B) Binding of EPR18855 and 2G1 antibodies to phosphorylated and monoSer2-phosphorylated peptides relative to peptide **35** (relative binding = 1). (C) Probing chelate and cluster effects upon binding of EPR18855, 2G1, and E1Z3G mAbs to hexaheptad peptides containing two or six pSer2 residues. Relative binding is based on the best monovalent binders: peptide **35** for E1Z3G and peptide **37** for antibodies EPR18855 and 2G1. Conditions: Immobilization: Ni-coated well + 100 μL of 30 nM CTD peptide in phosphate buffer (200 mM NaH_2_PO_4_, pH 7.4), 1 h, 25 °C; wash; primary antibody: for (A) 2-fold dilutions (from 1.48 nM) of antibody E1Z3G (A) or for (B, C) single dilution of E1Z3G (0.09 nM), EPR18855 (1.3 nM), and 2G1 (2.1 nM), 1 h, 37 °C; wash; secondary antibody: HRP-conjugated anti-rabbit antibody (1:4000 dilution), 30 min, 37 °C; wash; detection: i. 100 μL of TMB–H_2_O_2_ solution (1:1), 20 min, 25 °C, ii. 100 μL of 2 M H_2_SO_4_, 1 min, 25 °C. Washes and dilution of the antibodies were performed with NaH_2_PO_4_, 200 mM BSA (1% w/v), and 0.05% Tween-20 at pH 7.4. Assays were performed in triplicate with randomized positions of probes or antibodies.

To allow rapid screening, the His_6_-tagged CTD peptides were immobilized onto Ni^2+^-coated 96-well microtiter plates. The immobilized peptides were incubated with increasing concentrations of the anti-pS2 antibody E1Z3G. Detection of this antibody was achieved with a secondary horseradish peroxidase (HRP)-conjugated antibody used to trigger the tetramethylbenzidine (TMB) color reaction. For phosphorylated peptides **32**–**37**, absorbance increased with increasing concentration of anti-pS2 antibody (Figure 4A). This increase was not observed for unphosphorylated peptide **31**, confirming that no unspecific binding of the antibody to surfaces and the CTD sequence occurred. The titration curves for CTD peptides where pSer2 was shifted through the six heptads indicate that E1Z3G has the highest affinity for peptides having the phosphorylation in the central heptad. Dissociation constant *K*_d_ determined by curve fitting varied between *K*_d_ = 3.2 nM for **32**, having pS2 at the N-terminal heptad, and *K*_d_ = 0.5 nM for **35** with pS2 at heptad 4. Next, positional scanning was performed with anti-pS2 antibodies EPR18855 (Abcam) and 2G1 (Thermo Fisher/Invitrogen). Here, and in subsequent experiments, we refrained from obtaining titration curves and instead determined the fold change of antibody binding relative to that of a reference binder (Figure 4B). For most cases, the position of phosphorylation did not have much effect on binding of the two antibodies. Surprisingly, however, both EPR18855 and 2G1 had a very high affinity for peptide **37** (25–40-fold binding enhancement relative to **35**), which contained pSer2 within the C-terminal heptad.

In principle, different binding of the antibodies could result from varying the amounts and/or purities of the test peptides on the microplate surface. However, this cannot explain the opposing binding trends observed for three different antibodies, suggesting that differential binding is not caused by different microtiter plate loading but instead is due to different binding characteristics of the antibodies.

### Spatial Screening for Multivalency-Enhanced Binding

In many cases, antibody efficacies are improved by bivalency. It is therefore conceivable that the presence of two or more pSer2 residues within the CTD leads to increased binding of antibodies. Bivalency-enhanced binding can occur through the chelate effect and through statistical rebinding (cluster effect^60,61^). The chelate effect operates when the bivalent ligand, i.e., the bisphosphorylated CTD, bridges the two antigen binding sites of the antibody. The cluster effect would come into play when a second pSer2 residue comes into the vicinity of a pSer2-occupied antigen binding site, facilitating a rapid refilling after the release of the initially bound pSer2. To determine a distance–affinity relationship, we analyzed CTD peptides containing two pSer2 residues in increasing distance (Figure 4C). For the E1Z3G antibody, we assessed the increase in antibody binding relative to that of the best monovalent binder, phosphopeptide **35** (Figure 4A). Presentation of pSer2 residues in a distance of 1–5 heptads (**38**–**42**) enhanced the affinity to a negligible extent (<2-fold). The analysis of EPR18855 and 2G1 was based on phosphopeptide **37**, the monovalent binder with the highest affinity for the two antibodies (Figure 4B). Bivalency-enhanced binding was not observed.

A slightly different picture emerged when we investigated the cluster positioning of pSer2. In this analysis, the best monovalent binders (**35** for E1Z3G; **37** for EPR18855 and 2G1) were compared with the hexaphosphorylated peptide **7** and bivalent phosphopeptides **43** and **44**, which contained pSer2 at two adjacent heptads in internal position. A 5-fold enhancement of affinity was observed for the interaction of the E1Z3G antibody with hexaphosphopeptide **7**. Similarly, E1Z3G gained affinity when pSer2 in heptad 4 was accompanied by pSer2 in adjacent heptads (**43** and **44**). In contrast, a clustered presentation of pSer2 did not enhance binding of EPR18855 and 2G1 (see data for **7**, **38**, **43**, and **44**).

**Figure 5:**
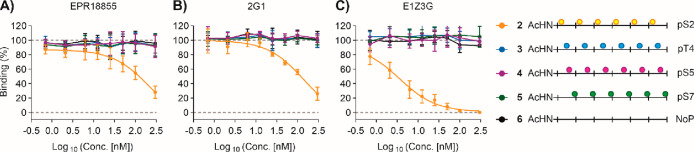
Binding specificity of antibodies (A) EPR18855, (B) 2G1, and (C) E1Z3G in interactions with hexaphosphorylated peptides (**2**–**5**) or unphosphorylated peptide (**6**) assessed in solution by competitive ELISA with immobilized phosphopeptide **35**. Conditions: immobilization: see caption to Figure 4; competitor: peptides **2**–**6** plated in dilution series; for subsequent steps see caption to Figure 4 and Supporting Information. The 100% binding value refers to binding of antibody to immobilized **35** in the absence of competitor.

For validation of the assay results, we performed competition assays with CTD peptides in solution, which were added in increasing concentrations to antibodies prior to incubation with the immobilized reference peptide **35**. As expected, the unphosphorylated peptide **6** was not able to compete against the immobilized peptide for antibody binding and neither were hexaphosphorylated CTD peptides **3**, **4**, and **5** containing pThr4, pSer5, or pSer7 residues (Figure 5). This behavior was evident for all three antibodies, demonstrating their specificity for pSer2. The hexa-pSer2 peptide **2** was a very efficient binder of E1Z3G, preventing its binding to the immobilized peptide **35** with an IC_50_ = 4 nM. Of note, with IC_50_ = 140 nM and 210 nM binding of EPR18855 and 2G1 to **35** was weaker, validating the results obtained with the immobilized hexa-pSer2 peptide **7**.

**Figure 6:**
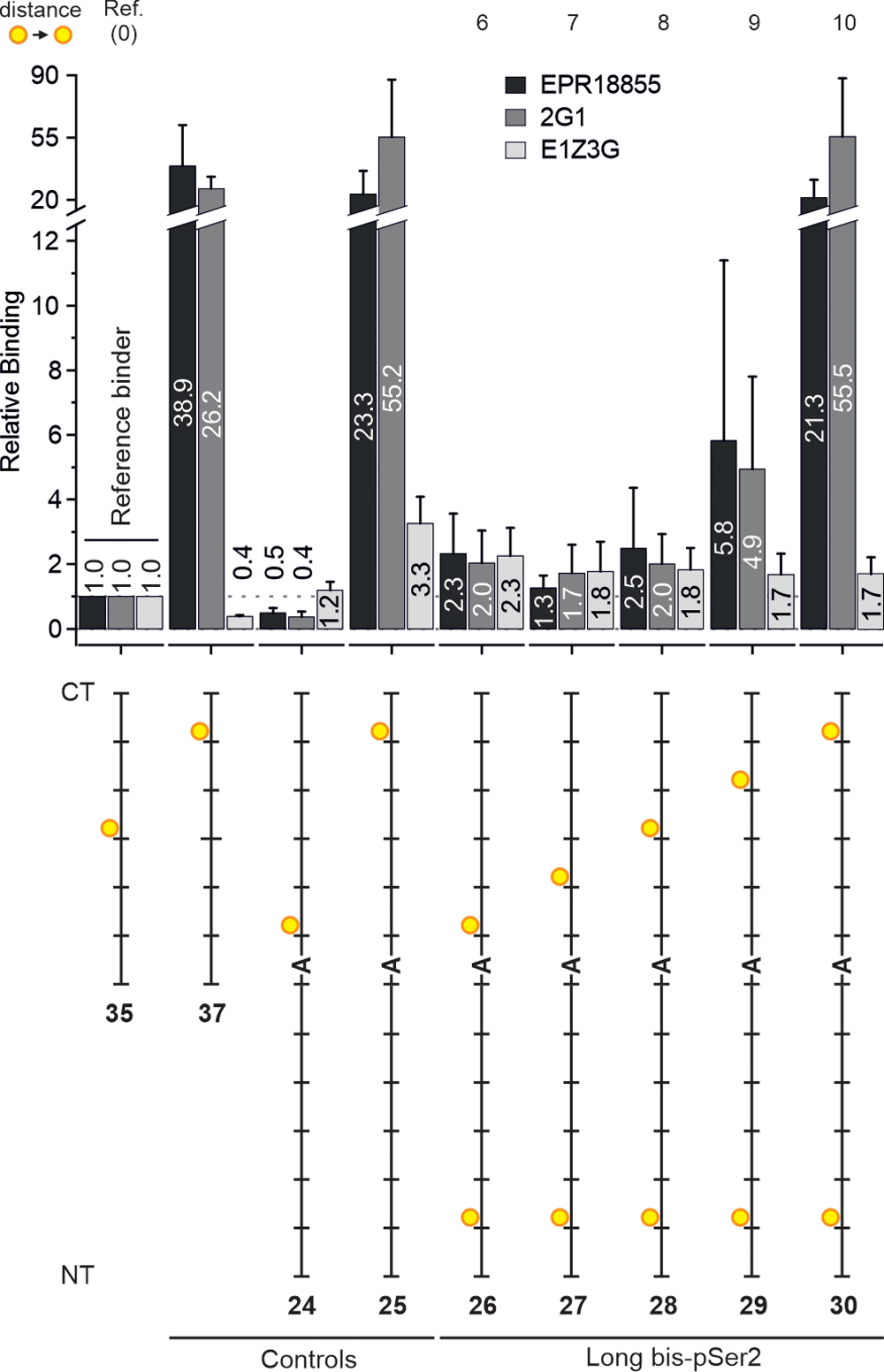
Probing chelate binding of mAbs EPR18855, 2G1, and E1Z3G to CTD dodecaheptad peptides arranging two pSer2 residues in up to 70 amino acid distance. Binding is characterized relative to peptide **35**. Conditions: see the caption to Figure 4.

The five-heptad distance provided by peptide **42** might not be long enough for chelating the antibodies. The spatial screening for bivalency was therefore continued by using CTD peptides with a length of 12 heptad repeats (Figure 6). For synthesis reasons, these peptides harbored a Ser → Ala mutation in heptad 7. Comparison of antibody binding to CTD peptides bearing pSer2 in heptad 4 (**35**) or 6 (**37**) of hexaheptads or heptad 8 (**24**) and 12 (**25**) of dodecaheptads revealed that this mutation was tolerated by all three antibodies when pSer2 was in a distal heptad. However, for antibodies EPR18855 and 2G1, recognition was impaired when pSer2 was in heptad 8 proximal to the mutation site (compare **24** with **35**). On the contrary, E1Z3G also tolerated this proximal mutation. In principle, therefore, the binding of E1Z3G could benefit from the simultaneous placement of pSer2 in heptad 2 and heptad 8 (in **26**). Again, however, the binding enhancement did not exceed a factor of 2 for bivalent pSer2 presentation, at either 42 amino acids (**26**) or 70 amino acids (**30**) distance. Similarly, the binding of EPR18855 and 2G1 for bivalent CTD phosphopeptides (**26**, **27**, **28**) remained at the level of the monovalent phosphopeptide (**35**) until the second pSer2 residue approached the C-terminal end (**29**, **30**), which is when an up to 40-fold clear increase in binding was observed. The measurements of these two antibodies with the hexaheptad set **32**–**37** (Figure 4B) had already shown evidence for a sudden increase of affinity for pSer2 at a C-terminal heptad, and therefore, we attributed the high affinity for biphosphorylated peptide **30** to the preferential binding to the C-terminal heptad. We conclude that within the set of biphosphorylated peptides spanning pSer2–pSer2 distances from 7 to 70 amino acids, there is no evidence for chelate-enhanced binding of E1Z3G, EPR18855, and 2G1 antibodies

### Influence of Phosphorylation on Thr4, Ser5, and Ser7 on Recognition of pSer2

**Figure 7:**
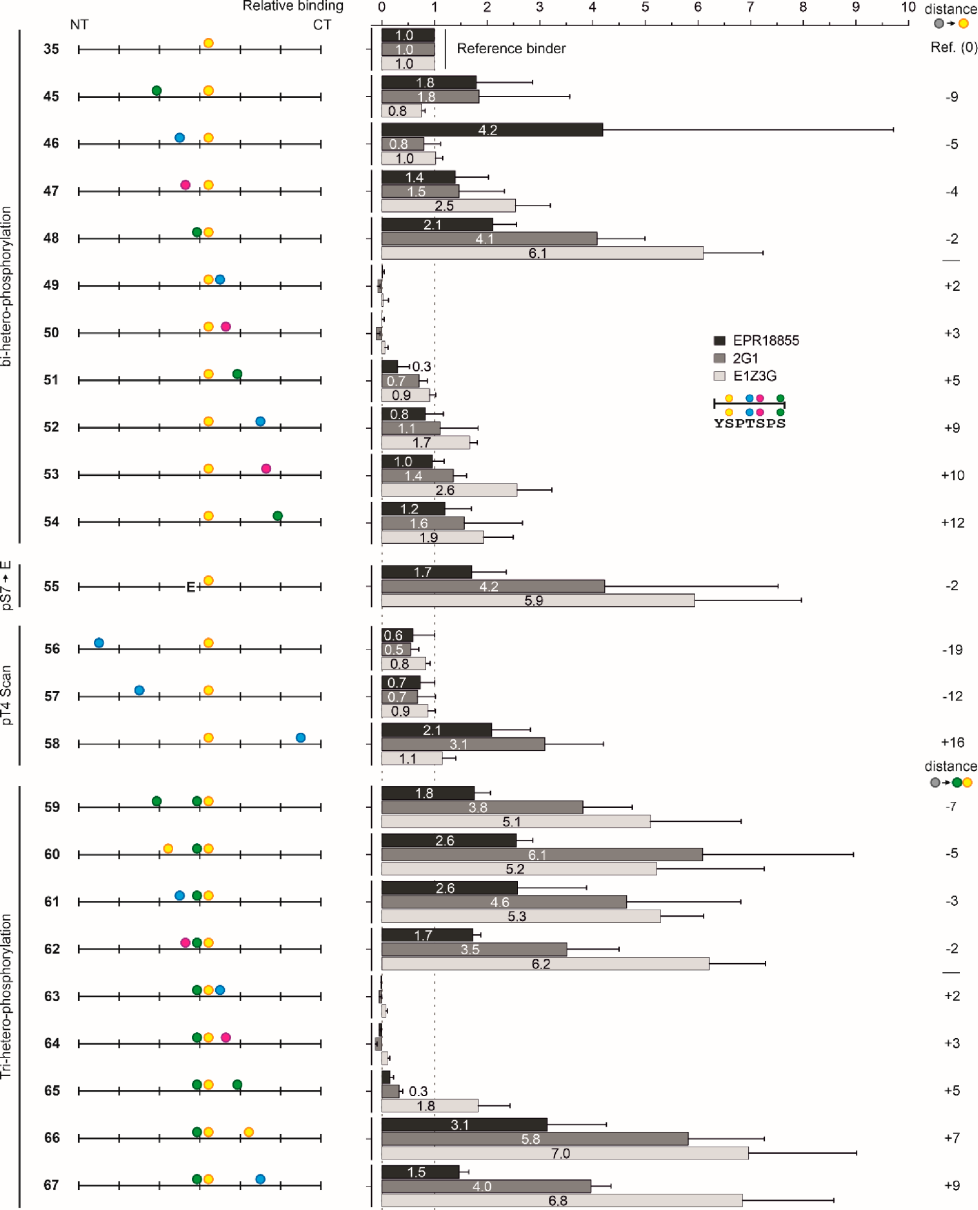
Probing the effect of second and third phosphorylations on binding of mAbs EPR18855, 2G1, and E1Z3G to pSer2-containing CTD peptides. Binding is characterized relative to peptide **35**. Values on the right side provide the distance between two phosphosites in number of amino acids (with negative and positive values indicating second phosphorylation in the N-terminal or C-terminal direction, respectively). The value for binding of EPR18855 with **46** is very likely an outlier. Conditions: see caption to Figure 4.

Dynamic CTD phosphorylation must include states in which different phosphoforms coexist. This raises the question of how other phosphorylation events influence the detection of pSer2 by antibodies. To address this question, we analyzed a set of peptides containing one phosphorylation fixed at Ser2 in heptad 4 and a second phosphorylation at threonine-4, serine-5, and serine-7 residues within adjacent heptads (Figure 7). For the 2G1 and E1Z3G antibodies, a surprising 4- and 6-fold enhancement of binding was observed when Ser7 in the downstream heptad was phosphorylated (peptide **48**, pSer7 in the −2-position to pSer2). The pSer7-induced binding enhancement was smaller for EPR18855. Control experiments showed that pSer7 peptides lacking a pSer2 site were recognized by neither antibody (peptide **77**, Figure S15). The enhancement effect was observed only when pSer7 was separated by one spacer amino acid in the N-terminal direction and was not observed for peptide **51** (+5-position) in which pSer2 and pSer7 were located in the same heptad. Of note, replacing pSer7 in heptad 3 with glutamic acid^62^ (peptide **55**) increases the affinity for the E1Z3G and 2G1 antibodies by 6- and 4-fold, respectively. This shows that glutamic acid can be a fairly good mimic of phosphorylated amino acids for some proteins. The enhanced antibody recognition of peptide **48** could be due to a high local concentration of negative charges facilitating the interaction with the positively charged amino acids of the antibody. This effect is, however, restricted to the next N-terminal neighbor of pSer2 and does not apply to upstream neighbors. On the contrary, we observed that a phosphorylation on Thr4 and Ser5 in +2- and +3-positions of the pSer2 (peptides **49**, +2 and **50**, +3) blocks the recognition by the anti-pS2 antibodies. The blocking effect by phosphothreonine was restricted to the +2-position and did not occur when pThr4 was positioned in other heptads (peptides **46**, **52**, and **56**–**58**). Remarkably, phosphorylation of Thr4 within the C-terminal heptad (peptide **58**) increased the binding of EPR18855 and 2G1 antibodies, indicating, once again, that these antibodies favor negative charges at the C-terminal end.

Scanning the effect of the second phosphorylations showed that a pSer7 in the −2-position (peptide **48**) increased antibody binding, whereas pThr4 and pSer5 in +2- and +3-positions (**49** and **50**, respectively) prevented recognition of pSer2. To investigate which factor is more important, we introduced additional phosphosites to the pSer7–pSer2 pair. A comparison of third phosphorylations in the N-terminal direction with those at Thr4 and Ser5 in peptides **63** and **64** showed that the negative effect exerted by phosphorylation at the +2- and +3-positions of pSer2 overrules the positive effect provided by phosphorylation in the −2-position.

With another set of phosphopeptides, we explored the influence of second and third phosphorylation when pSer2 was located in the C-terminal heptad, which provides an unusually high affinity for EPR18855 and 2G1 antibodies (Figure S16). The pSer7 residue in the −2-position only moderately increased the recognition of pSer2 within the C-terminal heptad. Again, pThr4 and pSer5 in the +2- and +3-positions completely abolished binding.

### Structural Studies

**Figure 8:**
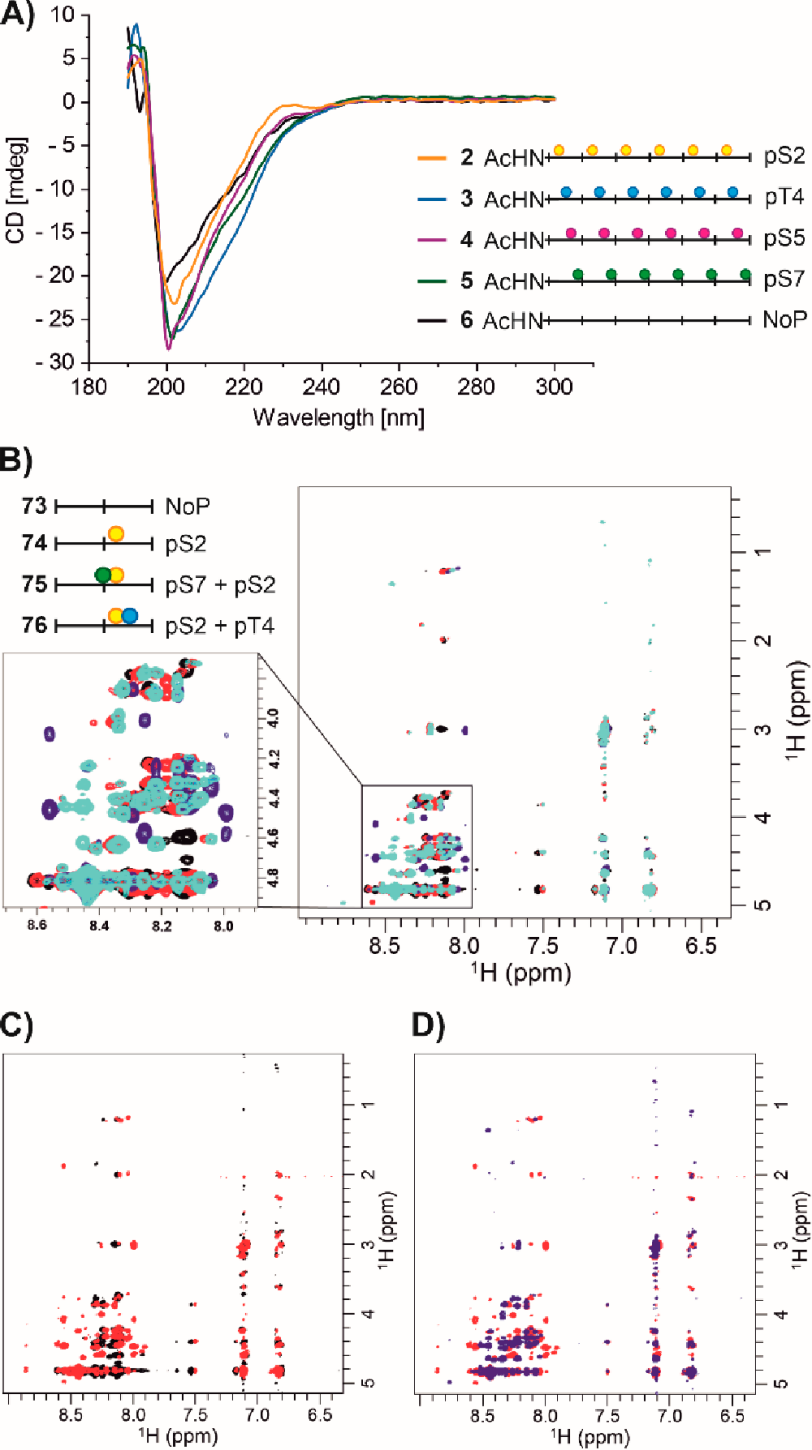
Structural studies of CTD peptides. (A) CD spectra of hexaphosphopeptides **2**–**5** and unphosphorylated peptide **6**. Conditions: 20 μM peptide, 100 mM NaF, 100 mM Tris-HCl, pH 7.2. ^1^H–^1^H-ROESY NMR spectra superimposing the spectra of (B) peptides **73** (black), **74** (red), **75** (blue), and **76** (cyan); (C) peptides **73** (black) and **75** (red); and (D) peptides **75** (red) and **76** (blue). Conditions: 0.5–1.0 mM peptide in H_2_O, 5% D_2_O, 0.1 mM 3-(trimethylsilyl)-1-propanesulfonic acid-*d*_6_ sodium salt (DSS), 4 mM NaN_3_, pH 5.5.

In its unphosphorylated form, the CTD is considered flexible. A rather compact structure is believed to be the result of a random coil conformation or, alternatively, a series of dynamically interconverting turns.^63^ Phosphorylation could induce more extended conformations due to charge repulsion or, on the contrary, induce turns by salt bridges. Such conformational equilibria could influence CTD recognition by antibodies. To obtain clues about phosphorylation-specific conformations, we used CD spectroscopy to search for preferred secondary structure motifs in CTD peptides. We hypothesized that if a specific amino acid in each of the six heptads of CTD peptides were phosphorylated, differences would be observed. However, the CD spectra of both unphosphorylated^64^ (**6**) and hexaphosphorylated (**2**–**5**) CTD peptides are consistent with a predominant random coil conformation (Figure 8A).

A notable detail is that among the phosphorylations studied the CD signature of hexa-pSer2 peptide **2** most closely resembled that of the unphosphorylated peptide **6**. Prediction of secondary structure elements using BestSel^65^ suggests that phosphorylation can increase the helix content from 6% for the unphosphorylated peptide **6** to up to 20% for hexa-pThr4 peptide **3** (Table S5). Although a large uncertainty in the prediction of secondary structural elements by fitted CD spectra should be noted, it seemed conceivable that altered conformational equilibria contribute to the reduction in antibody affinity caused by pThr4 in the +2-position.

The discrepancy between enhanced antibody binding when pSer2 is accompanied by pSer7 in the −2-position and abolished binding when pSer2 has a pThr4 in the +2-position occurred as a particularly noteworthy feature. To gain insight into the structural differences, a series of NMR experiments (1D-^1^H, ^1^H–^1^H-TOCSY, ^1^H–^1^H-ROESY, ^1^H–^13^C-HSQC) were performed. Anticipating a large overlap of resonances, we reduced the peptide length from six to two heptad repeats to facilitate NMR assignments. Four diheptad peptides were studied, containing only pSer2, pSer2 and pThr4 in the +2-position (decreased antibody affinity), pSer2 and pSer7 in the −2-position (increased antibody activity), or no phosphorylation (Figure 8B). In all cases, the NMR spectra are characteristic of largely disordered peptides. Furthermore, many signals are present in more than one set due to the *cis–trans* isomerization of proline, which significantly affects the resonance assignment. At pH 7.4, the amide resonances are completely broadened out (Figure S19). Therefore, a detailed analysis of homonuclear and heteronuclear 2D spectra was performed at pH 5.2. Similar patterns of NMR resonances were observed for all phosphorylated and nonphosphorylated peptides, indicating the absence of global structural changes upon phosphorylation. In the ^1^H–^13^C HSQC spectra, some phosphorylation-induced changes of chemical shifts were observed, e.g., for C^α^ (Figure S20). However, we did not observe a consistent trend that could be interpreted in terms of the presence/absence/formation of regular secondary structure in one or the other peptide. To obtain 3D structural information, ^1^H–^1^H-ROESY spectra were measured (Figure 8B–D). Again, signal overlap prevented a full resonance assignment. The ROESY signals did not change significantly when comparing mono-, di-, and nonphosphorylated variants of the CTD peptides. The peptides are very similarly disordered, and we conclude that differences in their binding to antibodies are most likely due to an induced fit mechanism rather than conformational selection.

## Discussion

The antibodies used in studies of the CTD code are powerful tools, but their binding properties are not fully known. Most binding studies focused on CTD peptides spanning two to three heptad repeats. However, controversial findings indicate that the size of the CTD peptides seems to matter.^28,30^ The optimized synthesis method presented by us facilitates screening and expands the range of CTD peptides by providing access to 48–90 amino acid long phosphoforms. Surprisingly, in the acidic environment, multiphosphorylated peptides form byproducts. The similarity to features previously reported for phosphotyrosine-containing peptides^43^ suggests the formation of depsipeptides. However, this side-reaction is reversible and, therefore, of no concern, when the phosphopeptides are applied at neutral or basic conditions.

Assaying >60 CTD phosphopeptides revealed that two (2G1 and EPR18855) of the three anti-pSer2 antibodies tested preferentially bound pSer2 at the C-terminal heptad. This pronounced positional selectivity may be due to the use of short phosphopeptide antigens in the generation of antibodies so that terminal amino acids may be involved in the recognition. In nature, the multiheptad repeats of the CTD do not extend to the extreme C-terminus. Therefore, a preferential binding of 2G1 and EPR18855 antibodies to pSer2 close to the C-terminus probably does not affect the functional analysis of CTD modifications in cell biological studies. However, it should be considered that the affinities of such antibodies are lower than expected from validation with short phosphopeptides. Of note, the antibody E1Z3G preferred pSer2 within internal heptads and has a 2 orders of magnitude higher target affinity than 2G1 and EPR18855.

With access to long phosphopeptides, we investigated whether the multivalent presentation of the phospho groups results in significantly increased antibody binding. Bridging of the two antigen binding sites by a bivalent binder can induce strong binding enhancements.^66,67^ Applied to CTD analysis in a cellular context, the degree of antibody binding could depend on both the number and distance between phosphosites. Recently, Shaw et al. reported a >1.500-fold increase in binding affinity for interactions of IgG antibodies with two ligands presented on rigid DNA-origami scaffolds in 36–160 Å distance.^68^ By contrast, our data showed that CTD peptides presenting two pSer2 residues in 14 to 70 amino acid distance afforded negligible (<2-fold) binding enhancements. Although desirable for applications in CTD analyses, the lack of bivalency-enhanced binding may seem counterintuitive. Given the random coil nature of serine- and threonine-phosphorylated hexaheptad CTD peptides confirmed by CD and NMR measurements, the phospho residues in the tested bisphosphorylated peptides are separated by up 70 Å distance, according to the Flory model.^69^ This should be within the 36–160 Å distance range accepted by IgG antibodies. However, in stark contrast to the DNA-origami structures studied by Shaw et al., the CTD is a very flexible scaffold. Since the magnitude of affinity enhancements provided by bivalent binding critically depends on the rigidity of the scaffold,^70^ the flexibility of the long CTDs probably prevents bivalency-enhanced recognition.

Although the three anti-pSer2-antibodies showed different binding affinities and positional selectivity, they were all equally affected by phosphorylation at Thr4 and Ser5 in the +2- and +3-position, respectively. It should be noted that phosphorylation at Ser2 occurs simultaneously with phosphorylation of Thr4 during the late phase of transcription.^19^ Therefore, if antibodies 2G1, EPR18855, and E1Z3G were used in combination with a pThr4-specific antibody, a double-positive result would indicate that pSer2 and pThr4 are located in different heptad repeats. Remarkably, our data show that binding of 2G1, EPR18855, and E1Z3G was improved by an additional pSer7 in the −2-position, though to different extent. This enhancement by the presence of a “noncanonical” phospho residue has previously only been observed for the H5 antibody, which shows enhanced binding when pSer2 is accompanied by a pSer5 in the −4- and +3-positions.^29,30^ Phosphorylation at Ser7 is an early event in transcription.^19^ During elongation, pSer7 levels decrease while pSer2 levels increase. If pSer7 residues were present in the vicinity of pSer2 during the transition from initiation to elongation, experiments with 2G1, EPR18855, or E1Z3G could lead to an overestimation of pSer2 levels.

Recognition patterns shared by all antibodies involved in a study could also indicate insufficient purity of the phosphopeptides tested or differences in the amount of immobilized material in the ELISA. However, UPLC-MS analysis of compounds containing pSer7, pThr4, or pSer5 in addition to pSer2 indicated similar purities (80–90%). In addition, the amount of phosphopeptide used during microtiter plate immobilization was carefully adjusted to equal levels, and immobilization was performed for 1 h, to allow quantitative loading.

## Conclusions

Different from previous studies on CTD antibody specificity, we used longer CTD peptides in which the phosphorylated residues were further away from the terminal ends of the peptide. This is closer to the naturally occurring situation. For this purpose, we optimized the challenging synthesis of multiphosphorylated peptides (up to six modifications) and prepared a library of 80 peptides with different modification patterns. HPLC-free purification and immobilization enabled rapid screening of the recognition profile of commercially available anti-pS2 mAbs. Our studies suggest that the binding of phospho-specific antibodies is only marginally enhanced by multivalent interaction, although it remains possible that multiphosphorylated CTDs may bind more antibodies than monophosphorylated ones. But synergistic affinity enhancements seem unlikely, given the high flexibility of the CTD peptides. The effect of clustered phosphorylation is more complex. The data show that the recognition of pSer2 can be both prevented and enhanced by additional phosphorylation in the vicinity. A striking observation is that additional phosphorylation at the +2- and +3-positions overrides the positive effect of pSer7 at −2 from that of pSer2. To the best of our knowledge, this is the first time that the effects of clustered triphosphorylation on anti-phospho-CTD antibody recognition have been systematically evaluated, and we conclude that if clustered phosphorylation occurs in native CTDs, antibody tools can lead to erroneous conclusions.

In conclusion, many techniques are now available to study the CTD code—mass spectrometry in particular^71^—but chemical synthesis, coupled with biological assays (ELISA, FP, and others), will be central to further discoveries due to their unrivaled precision. The CTD code is complicated and still not fully understood, but knowing more about the limitations of the main tools for detecting and mapping phosphorylations provides powerful insight in the quest to crack the code.

## Supporting Information Available

Materials and methods, synthetic protocols, ELISA protocols, additional data, and UPLC-MS data of the synthesized material (PDF)
