# Supplementary material for: Multiphosphorylation-Dependent Recognition of Anti-pS2 Antibodies against RNA Polymerase II C-Terminal Domain Revealed by Chemical Synthesis
Source: J Am Chem Soc. 2024 Apr 19;146(17):12074–86. doi: 10.1021/jacs.4c01902 (PMC11066871; doi:10.1021/jacs.4c01902)
Supplement: Supplementary file 1 — ja4c01902_si_001.pdf [file ja4c01902_si_001.pdf]

Supporting Information to:

Multiphosphorylation-dependent recognition of anti-pS2 antibodies against RNA polymerase II C-terminal domain revealed by chemical synthesis.

Emanuele Piemontese, Alina Herfort, Yulia Perevedentseva, Heiko M. Möller, Oliver Seitz

Institut für Chemie, Humboldt-Universität zu Berlin, 12489 Berlin, Deutschland

Institut für Chemie, Universität Potsdam, 14476 Golm, Deutschland

Table of Contents

1. Abbreviations **3**

2. Materials **4**

2.1 Antibodies *5*

3. Instruments **6**

4. Sequence of peptides **7**

5. Methods and Protocols **10**

5.1 Solid-phase peptide synthesis *10*

5.1.1 Optimization of the synthesis strategy 10

5.1.2 Optimized protocol for SPS of multi-phosphorylated peptides by Fmoc strategy 11

5.1.3 Synthesis of hexaphosphorylated peptides 2, 3, 4, 5 and unphosphorylated peptide 6 12

5.2 Synthesis of phosphopeptides with affinity purification *15*

5.3 Synthesis of dodecaheptad CTD peptides *16*

5.3.1 Fmoc-Tyr(tBu)-hydrazide resin 17

5.3.2 Synthesis of the N-terminal fragment thioesters 18

5.3.3 Synthesis of C-terminal fragments 20

5.3.4 Native Chemical Ligation (NCL) 22

5.3.5 Desulfurization 26

5.4 ELISAs *32*

5.4.1 Sample preparation 32

5.4.2 ELISA protocols 32

5.4.3 Additional material ELISA 33

5.5 Structural studies *37*

5.5.1 Circular dichroism measurements 37

5.5.2 Synthesis of peptides samples for structural studies by NMR spectroscopy 38

5.5.3 NMR measurements 40

6. Characterization of peptides tested in ELISA for antibody binding **42**

7. References **65**

1. Abbreviations

- **ACN**: acetonitile
- **Bzl**: Benzyl-
- **Calcd.**: calculated
- **CD**: circular dichroism
- **DBU**: 1,8-Diazabicyclo(5.4.0)undec-7-ene
- **DCM**: dichloromethane
- **DIC**: N,N'-Diisopropylcarbodiimide
- **DiPEA**: N,N-Diisopropylethylamine
- **DMF**: N,N-Dimethylmethanamide
- **DSS**: 3-(Trimethylsilyl)-1-propanesulfonic acid-d6 sodium salt
- **EDT**: Ethane-1,2-dithiol
- **FA**: formic acid
- **Gdn-HCl**: guanidinium hydrochloride
- **HATU**: 1-[Bis(dimethylamino)methylene]-1H-1,2,3-triazolo[4,5-b]pyridinium 3-oxide hexafluorophosphate
- **HBTU**: [benzotriazol-1-yloxy(dimethylamino)methylidene]-dimethylazanium;hexafluorophosphate
- **HCTU**: O-(1H-6-Chlorobenzotriazole-1-yl)-1,1,3,3-tetramethyluronium hexafluorophosphate
- **HOBt**: 1H-1,2,3-Benzotriazol-1-ol
- **HPLC**: high-performance liquid chromatography
- **mAb**: mono-clonal antibody
- **MeTHF**: 2-Methyltetrahydrofuran
- **MPA**: 3-mercaptoproanoic acid
- **MPAA**: 4-Mercaptophenylacetic acid
- **MS**: mass spectrometry
- **MW**: microwave
- **NCL**: Native Chemical Ligation
- **NMM**: N-Methylmorpholine
- **NMP**: N-methyl-2-pyrrolidone
- **NMR**: nuclear magnetic resonance
- **NTA**: 2,2',2''-Nitrilotriacetic acid
- **PEG**: polyethylenglycol
- **PES**: polyethersulfone
- **PS**: polystyrene
- **RP-HPLC**: reverse phase high-performance liquid chromatography
- **SPPS**: solid-phase peptide synthesis
- **TBAF**: N,N,N-Tributylbutan-1-aminium fluoride
- **TFA**: trifluoroacetic acid
- **TIS**: tri(propan-2-yl)silane
- **TMB**: 3,3',5,5'-Tetramethylbenzidine
- **Tris**: tris(hydroxymethyl)aminomethane
- **Trt**: trityl
- **UPLC**: ultra-performance liquid chromatography

2. Materials

- Fmoc-protected standard amino acids building block, HATU, HBTU, HCTU, DIC, Oxyma and DBU were purchased from *Carbolution Chemicals* (St. Ingbert, Germany).
- Fmoc-Ser(PO_3BzlH)-OH, Fmoc-Thr(PO_3BzlH)-OH and Fmoc-HN-(CH_2)$_2$-PEG$_6$-COOH were purchased from *BLDPharm* (Reinbeck, Germany).
- Hydrazine monohydrate, MPAA, NaH_2PO_4 anhydrous, TIS, Ac_2O, TBAF trihydrated, 1-octanethiol, acetylacetone, DSS-d6 (98 atom %D), $SOCl_2$ and Tween-20 for molecular biology were purchased from *Sigma-Aldrich* (St. Louis, MO, USA).
- Phenol was purchased from *TCI Deutschland* (Eschborn, Germany).
- MPA, formic acid and acetic acid were purchased from *Alfa Aesar* (Ward Hill, MA, USA).
- EDT was purchased from Acros (Fair Lawn, NJ, USA).
- Pierce™ TMB Substrate Kit (H_2O_2 and TMB solutions) and Pierce™ Nickel Coated Plates were purchased from *Thermo Fisher Scientific* (Waltham, MA, USA).
- Sulfuric acid, DMF for synthesis and acetonitrile (HPLC grade) were purchased from *VWR* (Darmstadt, Germany).
- HOBt monohydrate, Fmoc-Cl, 2,6-lutidine, 2-MeTHF and $NaBEt_4$ were purchased from *abcr* (Karlsruhe, Germany)
- TCEP HCl, Tris HCl, NaF, TFA for peptide synthesis, DiPEA, Na-citrate, piperidine for synthesis, NMM for synthesis, NMP for synthesis, Albumin fraction V (NZ-origin) for biochemistry and molecular biology and Gdn-HCl were purchased from *Carl Roth* (Karlsruhe, Germany).
- NaN_3 was purchased from *Riedel-de Haen* (Seelze, Deutschland).
- D_2O was purchased from *Eurisotop* (Saint-Aubin, France)
- Ni-NTA Agarose resin was purchased from *SERVA* (Heidelberg, Germany).
- Fmoc Ser(tBu)-TentaGel® R Trt resin (resin loading 0.16 mmol/g), TentaGel® R RAM (resin loading 0.22 mmol/g) and TentaGel® R Trt-Cl were purchased from *Rapp Polymere* (Tübingen, Germany).
- NAP-5 Columns Sephadex G-25 DNA grade were purchased from *GE Healthcare* (Chicago, IL, USA).
- Non-binding 96-well black microplate, PS, F-bottom µClear®, were purchased from *Greiner Bio-One* (Kremsmünster, Austria).
- Before usage, prepared buffers were filtered through Rapid-Flow™ Sterile filters with PES-membrane from *Nalgene* (Rochester, New York, USA)
- Ultrapure water for HPLC eluents, buffers and dissolution of peptides was prepared with an Astacus system (*membraPure*, Henningsdorf, Germany).
- Commercially available compounds were used without further purification. Dry solvents were taken from a Solvent Purification System SPS 800 (*MBraun*, Garching, Germany).

2.1 Antibodies

The antibodies tested and used in the manuscript are:

Primary (recombinant rabbit IgG, monoclonal, anti-Phospho-RNA pol II CTD (Ser2))

- *Cell Signaling Technology* (Danvers, MA, USA)

  Product code: 13499

  Clone: E1Z3G

  Lot 1 concentration: 56 µg/ml (0,37 µM)

  Lot 3 concentration: 56 µg/ml (0,37 µM)

- *Abcam* (Cambridge, United Kingdom)

  Product code: ab193468

  Clone: EPR18855

  Lot 1001234-1 concentration: 0.777 mg/ml (5.18 µM)

- *Thermo Fisher Scientific (Invitrogen)* (Waltham, MA, USA)

  Product code: MA5-33187

  Clone: 2G1

  Lot YA3819041 concentration: 1.25 mg/ml (8.34 µM)

  Lot YE3916981 concentration: 1 mg/ml (6.67 µM)

Secondary (Anti-rabbit IgG, HRP-linked Antibody)

- *Cell Signaling Technology*

  Product code: 7074

  Lot 26 concentration: 66 µg/ml

  Lot 32 concentration: 60 µg/ml

3. Instruments

Automated SPPS was carried out on a MultiPep RS peptide synthesizer (*Intavis*, Cologne, Germany) in 10 µmol fritted columns purchased from *Carl Roth* (Karlsruhe, Germany). Microwave-assisted SPPS was performed using an Initiator+ Alstra synthesizer from *Biotage* (Uppsala, Sweden).

Post-SPPS yield monitoring was performed on *BioRad* (Feldkirchen, Germany) SmartSpec Plus spectrophotometer.

Semi-preparative RP-HPLC purifications were carried out by using an *Agilent* 1260 Infinity II Series (Santa Clara, CA, USA) system and a Polaris 5 C18-A column (250 x 10.0 mm) from *Varian* (Lake Forest, CA, USA) with a binary mixture of A1 (98.9 % H_2O, 1 % ACN, 0.1 % TFA) and B1 (98.9 % ACN, 1 % H_2O, 0.1 % TFA) as a mobile phase (flow = 6 mL/min) in a linear gradient as described.

Analytical ultra-high-performance liquid chromatography (UPLC) was performed on an Acquity UPLC on an Acquity H-Class system (*Waters*, Milford, MA, USA) equipped with a PDA (λ = 210 nm) and QDa detector using an Acquity UPLC CSH C18 (2.1 x 50 mm; 1.7 µm; 130Å). Analyses were performed using either buffer system A1/B1 (same as semi-preparative HPLC) or A2/B2 (A2: 98.9 % H_2O, 1 % ACN, 0.1 % FA, B2: 98.9 % ACN, 1 % H_2O, 0.1 % FA) as mobile phase. Samples were eluted at 50°C.

High-resolution ESI-MS spectra were recorded on a Waters H-class instrument system. Samples were eluted with a flow rate of 0.3 mL/min at 40 °C. The following gradient was used: A: 0.01% FA in H2O; B: 0.01% FA in ACN. 5% B: 0-0.5 min; 5 to 95% B: 0.5-3.5 min; 95% B: 3.5-4 min. Mass analysis was conducted with a Waters XEVO G2-XS QTof analyzer and the recorded data was subsequently analyzed using the provided built-in software. These measurements were performed at the facility of Leibniz-Forschungsinstitut für Molekulare Pharmakologie (Berlin, Germany). We are thankful to Dr. Christian Stieger and Prof. Christian Hackenberger for the support.

Peptide solution concentration was determined by measuring the optical density at λ = 274 nm on a NanoDrop ND-1000 (*PeqLab*, Erlangen, Germany) spectrophotometer against a buffer blank. The extinction coefficient of tyrosine at this wavelength is 1400 $M^{-1}cm^{-1}$ and the coefficient for each peptide has been calculated by multiplying this value by the number of tyrosines in the sequence.

Microtiter plates were washed on a *Tecan* (Männedorf, Switzerland) Hydrospeed plate washer.

Optical density after the biological assays in microplates was measured on a *PerkinElmer* Victor X5 Multilabel plate reader (Waltham, MA, USA), equipped with P450 nm CW-lamp filters.

4. Sequence of peptides

Code	*HisTag*	*PEG*	*1*	*2*	*3*	*4*	*5*	*6*	*7*	*8*	*9*	*10*	*11*	*12*	*Figure Ref.*
**1**	HHHHHH	-	Y**S**PTSPS	Y**S**PTSPS	Y**S**PTSPS	Y**S**PTSPS	Y**S**PTSPS	Y**S**PTSPS	-OH						1, S1
**2**	-	Ac-	Y**S**PTSPS	Y**S**PTSPS	Y**S**PTSPS	Y**S**PTSPS	Y**S**PTSPS	Y**S**PTSPS	-NH_2						1, 7, S2
**3**	-	Ac-	YSP**T**SPS	YSP**T**SPS	YSP**T**SPS	YSP**T**SPS	YSP**T**SPS	YSP**T**SPS	-NH_2						1, 7, S2
**4**	-	Ac-	YSPT**S**PS	YSPT**S**PS	YSPT**S**PS	YSPT**S**PS	YSPT**S**PS	YSPT**S**PS	-NH_2						1, 7, S2
**5**	-	Ac-	YSPTSP**S**	YSPTSP**S**	YSPTSP**S**	YSPTSP**S**	YSPTSP**S**	YSPTSP**S**	-NH_2						1, 7, S2
**6**	-	Ac-	YSPTSPS	YSPTSPS	YSPTSPS	YSPTSPS	YSPTSPS	YSPTSPS	-NH_2						1, 7, S2
**7**	HHHHHH	PEG6	Y**S**PTSPS	Y**S**PTSPS	Y**S**PTSPS	Y**S**PTSPS	Y**S**PTSPS	Y**S**PTSPS	-NH_2						2, 6B, S4
**8**	HHHHHH	PEG6	YSPTSPS	YSPTSPS	YSPTSPS	YSPTSPS	YSPTSPS	YSPTSPS	Y-NHNH2						S6
**9**	HHHHHH	PEG6	YSPTSPS	Y**S**PTSPS	YSPTSPS	YSPTSPS	YSPTSPS	YSPTSPS	Y-NHNH2						3, S6
**10**	HHHHHH	PEG6	YSPTSPS	YSPTSPS	YSPTSPS	YSPTSPS	YSPTSPS	YSPTSPS	Y-MPA						S6, S7, S9
**11**	HHHHHH	PEG6	YSPTSPS	Y**S**PTSPS	YSPTSPS	YSPTSPS	YSPTSPS	YSPTSPS	Y-MPA						3, S6, S7, S9
**12**	-	-	CPTSPS	Y**S**PTSPS	YSPTSPS	YSPTSPS	YSPTSPS	YSPTSPS	-NH_2						3, S8, S9
**13**	-	-	CPTSPS	YSPTSPS	Y**S**PTSPS	YSPTSPS	YSPTSPS	YSPTSPS	-NH_2						S8, S9
**14**	-	-	CPTSPS	YSPTSPS	YSPTSPS	Y**S**PTSPS	YSPTSPS	YSPTSPS	-NH_2						S8, S9
**15**	-	-	CPTSPS	YSPTSPS	YSPTSPS	YSPTSPS	Y**S**PTSPS	YSPTSPS	-NH_2						S8, S9
**16**	-	-	CPTSPS	YSPTSPS	YSPTSPS	YSPTSPS	YSPTSPS	Y**S**PTSPS	-NH_2						S8, S9
**17**	HHHHHH	PEG6	YSPTSPS	YSPTSPS	YSPTSPS	YSPTSPS	YSPTSPS	YSPTSPS	Y**C**PTSPS	Y**S**PTSPS	YSPTSPS	YSPTSPS	YSPTSPS	YSPTSPS-NH_2	S9, S10, S11
**18**	HHHHHH	PEG6	YSPTSPS	YSPTSPS	YSPTSPS	YSPTSPS	YSPTSPS	YSPTSPS	Y**C**PTSPS	YSPTSPS	YSPTSPS	YSPTSPS	YSPTSPS	Y**S**PTSPS-NH_2	S9, S10, S11
**19**	HHHHHH	PEG6	YSPTSPS	Y**S**PTSPS	YSPTSPS	YSPTSPS	YSPTSPS	YSPTSPS	Y**C**PTSPS	Y**S**PTSPS	YSPTSPS	YSPTSPS	YSPTSPS	YSPTSPS-NH_2	3, S9, S10, S11
**20**	HHHHHH	PEG6	YSPTSPS	Y**S**PTSPS	YSPTSPS	YSPTSPS	YSPTSPS	YSPTSPS	Y**C**PTSPS	YSPTSPS	Y**S**PTSPS	YSPTSPS	YSPTSPS	YSPTSPS-NH_2	S9, S10, S11
**21**	HHHHHH	PEG6	YSPTSPS	Y**S**PTSPS	YSPTSPS	YSPTSPS	YSPTSPS	YSPTSPS	Y**C**PTSPS	YSPTSPS	YSPTSPS	Y**S**PTSPS	YSPTSPS	YSPTSPS-NH_2	S9, S10, S11
**22**	HHHHHH	PEG6	YSPTSPS	Y**S**PTSPS	YSPTSPS	YSPTSPS	YSPTSPS	YSPTSPS	Y**C**PTSPS	YSPTSPS	YSPTSPS	YSPTSPS	Y**S**PTSPS	YSPTSPS-NH_2	S9, S10, S11
**23**	HHHHHH	PEG6	YSPTSPS	Y**S**PTSPS	YSPTSPS	YSPTSPS	YSPTSPS	YSPTSPS	Y**C**PTSPS	YSPTSPS	YSPTSPS	YSPTSPS	YSPTSPS	Y**S**PTSPS-NH_2	S9, S10, S11
**24**	HHHHHH	PEG6	YSPTSPS	YSPTSPS	YSPTSPS	YSPTSPS	YSPTSPS	YSPTSPS	Y**A**PTSPS	Y**S**PTSPS	YSPTSPS	YSPTSPS	YSPTSPS	YSPTSPS-NH_2	8, S11, S12
**25**	HHHHHH	PEG6	YSPTSPS	YSPTSPS	YSPTSPS	YSPTSPS	YSPTSPS	YSPTSPS	Y**A**PTSPS	YSPTSPS	YSPTSPS	YSPTSPS	YSPTSPS	Y**S**PTSPS-NH_2	8, S11, S12
**26**	HHHHHH	PEG6	YSPTSPS	Y**S**PTSPS	YSPTSPS	YSPTSPS	YSPTSPS	YSPTSPS	Y**A**PTSPS	Y**S**PTSPS	YSPTSPS	YSPTSPS	YSPTSPS	YSPTSPS-NH_2	3, 8, S11, S12
**27**	HHHHHH	PEG6	YSPTSPS	Y**S**PTSPS	YSPTSPS	YSPTSPS	YSPTSPS	YSPTSPS	Y**A**PTSPS	YSPTSPS	Y**S**PTSPS	YSPTSPS	YSPTSPS	YSPTSPS-NH_2	8, S11, S12
**28**	HHHHHH	PEG6	YSPTSPS	Y**S**PTSPS	YSPTSPS	YSPTSPS	YSPTSPS	YSPTSPS	Y**A**PTSPS	YSPTSPS	YSPTSPS	Y**S**PTSPS	YSPTSPS	YSPTSPS-NH_2	8, S11, S12

Code	*HisTag*	*PEG*	*1*	*2*	*3*	*4*	*5*	*6*	*7*	*8*	*9*	*10*	*11*	*12*	*Figure Ref.*
**29**	HHHHHH	PEG6	YSPTSPS	YSPTSPS	YSPTSPS	YSPTSPS	YSPTSPS	YSPTSPS	YAPTSPS	YSPTSPS	YSPTSPS	YSPTSPS	YSPTSPS	YSPTSPS-NH_2	8, S11, S12
**30**	HHHHHH	PEG6	YSPTSPS	YSPTSPS	YSPTSPS	YSPTSPS	YSPTSPS	YSPTSPS	YAPTSPS	YSPTSPS	YSPTSPS	YSPTSPS	YSPTSPS	YSPTSPS-NH_2	8, S11, S12
**31**	HHHHHH	PEG6	YSPTSPS	YSPTSPS	YSPTSPS	YSPTSPS	YSPTSPS	YSPTSPS	-NH_2						4, 5
**32**	HHHHHH	PEG6	YSPTSPS	YSPTSPS	YSPTSPS	YSPTSPS	YSPTSPS	YSPTSPS	-NH_2						4, 5
**33**	HHHHHH	PEG6	YSPTSPS	YSPTSPS	YSPTSPS	YSPTSPS	YSPTSPS	YSPTSPS	-NH_2						4, 5
**34**	HHHHHH	PEG6	YSPTSPS	YSPTSPS	YSPTSPS	YSPTSPS	YSPTSPS	YSPTSPS	-NH_2						4, 5
**35**	HHHHHH	PEG6	YSPTSPS	YSPTSPS	YSPTSPS	YSPTSPS	YSPTSPS	YSPTSPS	-NH_2						4, 5, 6B, 7, 8, 9, S13, S14
**36**	HHHHHH	PEG6	YSPTSPS	YSPTSPS	YSPTSPS	YSPTSPS	YSPTSPS	YSPTSPS	-NH_2						4, 5
**37**	HHHHHH	PEG6	YSPTSPS	YSPTSPS	YSPTSPS	YSPTSPS	YSPTSPS	YSPTSPS	-NH_2						4, 5, 6A-B, 8, S13
**38**	HHHHHH	PEG6	YSPTSPS	YSPTSPS	YSPTSPS	YSPTSPS	YSPTSPS	YSPTSPS	-NH_2						6A
**39**	HHHHHH	PEG6	YSPTSPS	YSPTSPS	YSPTSPS	YSPTSPS	YSPTSPS	YSPTSPS	-NH_2						6A
**40**	HHHHHH	PEG6	YSPTSPS	YSPTSPS	YSPTSPS	YSPTSPS	YSPTSPS	YSPTSPS	-NH_2						6A
**41**	HHHHHH	PEG6	YSPTSPS	YSPTSPS	YSPTSPS	YSPTSPS	YSPTSPS	YSPTSPS	-NH_2						6A
**42**	HHHHHH	PEG6	YSPTSPS	YSPTSPS	YSPTSPS	YSPTSPS	YSPTSPS	YSPTSPS	-NH_2						6A
**43**	HHHHHH	PEG6	YSPTSPS	YSPTSPS	YSPTSPS	YSPTSPS	YSPTSPS	YSPTSPS	-NH_2						6B
**44**	HHHHHH	PEG6	YSPTSPS	YSPTSPS	YSPTSPS	YSPTSPS	YSPTSPS	YSPTSPS	-NH_2						6B
**45**	HHHHHH	PEG6	YSPTSPS	YSPTSPS	YSPTSPS	YSPTSPS	YSPTSPS	YSPTSPS	-NH_2						9
**46**	HHHHHH	PEG6	YSPTSPS	YSPTSPS	YSPTSPS	YSPTSPS	YSPTSPS	YSPTSPS	-NH_2						9
**47**	HHHHHH	PEG6	YSPTSPS	YSPTSPS	YSPTSPS	YSPTSPS	YSPTSPS	YSPTSPS	-NH_2						9
**48**	HHHHHH	PEG6	YSPTSPS	YSPTSPS	YSPTSPS	YSPTSPS	YSPTSPS	YSPTSPS	-NH_2						9, S13
**49**	HHHHHH	PEG6	YSPTSPS	YSPTSPS	YSPTSPS	YSPTSPS	YSPTSPS	YSPTSPS	-NH_2						9
**50**	HHHHHH	PEG6	YSPTSPS	YSPTSPS	YSPTSPS	YSPTSPS	YSPTSPS	YSPTSPS	-NH_2						9
**51**	HHHHHH	PEG6	YSPTSPS	YSPTSPS	YSPTSPS	YSPTSPS	YSPTSPS	YSPTSPS	-NH_2						9
**52**	HHHHHH	PEG6	YSPTSPS	YSPTSPS	YSPTSPS	YSPTSPS	YSPTSPS	YSPTSPS	-NH_2						9
**53**	HHHHHH	PEG6	YSPTSPS	YSPTSPS	YSPTSPS	YSPTSPS	YSPTSPS	YSPTSPS	-NH_2						9
**54**	HHHHHH	PEG6	YSPTSPS	YSPTSPS	YSPTSPS	YSPTSPS	YSPTSPS	YSPTSPS	-NH_2						9
**55**	HHHHHH	PEG6	YSPTSPS	YSPTSPS	YSPTSPE	YSPTSPS	YSPTSPS	YSPTSPS	-NH_2						9
**56**	HHHHHH	PEG6	YSPTSPS	YSPTSPS	YSPTSPS	YSPTSPS	YSPTSPS	YSPTSPS	-NH_2						9
**57**	HHHHHH	PEG6	YSPTSPS	YSPTSPS	YSPTSPS	YSPTSPS	YSPTSPS	YSPTSPS	-NH_2						9
**58**	HHHHHH	PEG6	YSPTSPS	YSPTSPS	YSPTSPS	YSPTSPS	YSPTSPS	YSPTSPS	-NH_2						9
**59**	HHHHHH	PEG6	YSPTSPS	YSPTSPS	YSPTSPS	YSPTSPS	YSPTSPS	YSPTSPS	-NH_2						9

Code	*HisTag*	*PEG*	*1*	*2*	*3*	*4*	*5*	*6*	*7*	*8*	*9*	*10*	*11*	*12*	*Figure Ref.*
60	HHHHHH	PEG6	YSPTSPS	YSPTSPS	Y**S**PTSP**S**	Y**S**PTSPS	YSPTSPS	YSPTSPS	$-NH_2$						9
61	HHHHHH	PEG6	YSPTSPS	YSPTSPS	YSP**T**SP**S**	Y**S**PTSPS	YSPTSPS	YSPTSPS	$-NH_2$						9
62	HHHHHH	PEG6	YSPTSPS	YSPTSPS	YSPT**S**P**S**	Y**S**PTSPS	YSPTSPS	YSPTSPS	$-NH_2$						9
63	HHHHHH	PEG6	YSPTSPS	YSPTSPS	YSPTSP**S**	Y**S**P**T**SPS	YSPTSPS	YSPTSPS	$-NH_2$						9
64	HHHHHH	PEG6	YSPTSPS	YSPTSPS	YSPTSP**S**	Y**S**PT**S**PS	YSPTSPS	YSPTSPS	$-NH_2$						9
65	HHHHHH	PEG6	YSPTSPS	YSPTSPS	YSPTSP**S**	Y**S**PTSP**S**	YSPTSPS	YSPTSPS	$-NH_2$						9
66	HHHHHH	PEG6	YSPTSPS	YSPTSPS	YSPTSP**S**	Y**S**PTSPS	Y**S**PTSPS	YSPTSPS	$-NH_2$						9
67	HHHHHH	PEG6	YSPTSPS	YSPTSPS	YSPTSP**S**	Y**S**PTSPS	YSP**T**SPS	YSPTSPS	$-NH_2$						9
68	HHHHHH	PEG6	YSPTSPS	YSPTSPS	YSPTSPS	YSPTSPS	YSPTSP**S**	Y**S**PTSPS	$-NH_2$						S13
69	HHHHHH	PEG6	YSPTSPS	YSPTSPS	YSPTSPS	YSPTSPS	YSPTSPS	Y**S**P**T**SPS	$-NH_2$						S13
70	HHHHHH	PEG6	YSPTSPS	YSPTSPS	YSPTSPS	YSPTSPS	YSPTSPS	Y**S**PT**S**PS	$-NH_2$						S13
71	HHHHHH	PEG6	YSPTSPS	YSPTSPS	YSPTSPS	YSPTSPS	YSPTSPS	Y**S**PTSP**S**	$-NH_2$						S13
72	HHHHHH	PEG6	YSPTSPS	YSPTSPS	YSPTSPS	YSPTSPS	YSPTSP**S**	Y**S**P**T**SPS	$-NH_2$						S13
73	-	-	YSPTSPS	YSPTSPS	$-NH_2$										
74	-	-	YSPTSPS	Y**S**PTSPS	$-NH_2$										
75	-	-	YSPTSP**S**	Y**S**PTSPS	$-NH_2$										
76	-	-	YSPTSPS	Y**S**P**T**SPS	$-NH_2$										
77	HHHHHH	PEG6	YSPTSPS	YSPTSPS	YSPTSP**S**	YSPTSPS	YSPTSPS	YSPTSPS	$-NH_2$						S14
78	HHHHHH	PEG6	YSPTSPS	YSPTSPS	YSPTSPS	YSPTSPS	YSPTSPS	YSPTSP**S**	$-NH_2$						S14
79	HHHHHH	PEG6	YSPTSPS	YSPTSPS	YSPTSPS	Y**SA**TSPS	YSPTSPS	YSPTSPS	$-NH_2$						S14
80	HHHHHH	PEG6	YSPTSPS	YSPTSPS	YSPTSPS	Y**SAT**SPS	YSPTSPS	YSPTSPS	$-NH_2$						S14

Table S1 – **Overview of the peptides synthesized** and reference to sections of the manuscript with characterization data. Letters in red mark the position of phosphorylation.

5. Methods and Protocols

5.1 Solid-phase peptide synthesis

5.1.1 Optimization of the synthesis strategy

The optimization of the synthesis of multi-phosphorylated peptides was done using hexaphosphopeptide **1** as the model. All the tests were performed on the Trt-TentaGel resin in a 25 µmol scale.

- *Deprotection*: removal of the Fmoc-group by treatment of the resin with the deprotection solution (800 µl, 2x5 min for the first 20 residues, 2x10 min for the next ones). Subsequently, the resin was washed with DMF (4x, 1000 µl).
- *Coupling*: Fmoc-protected amino acids, dissolved in a solution of NMP containing the additives (220 µl for 5.25 eq. and 345 µl for 8.25 eq.), were activated with HCTU (210 µl for 5 eq. and 335 µl for 8 eq.) and the base (95 µl of NMM 4M in DMF for 15 eq., 150 µl for 24 eq., or 105 µl of DiPEA) and transferred to the resin (coupling time 30 min for the first 20 residues, 60 min for subsequent couplings). Subsequently, the resin was washed with DMF (3x, 1000 µl).
- *Capping*: the resin was treated with DMF:Ac_2O:lutidine (89:5:6 v/v/v, 800 µl) for 5 min to block unreacted amino groups. Subsequently, the resin was washed with DMF (3x, 1000 µl).
- *Cleavage*: the resin was washed with DCM (3x) and dried under vacuum overnight. Then the acid cleavage solution (7.5 ml) was added to the dried resin. After 2 hours the syringe reactor was drained.

The model peptide was also synthesized employing the microwave synthesizer, following the protocol published by Harris et al.,[1] on RinkAmide resin with a scale of 30 µmol. Fmoc-deprotection was performed at room temperature (twice, 5 + 15 min.). During the coupling step, the reactor was irradiated with MW heating at 72°C for the phosphorylated residues (15 min., 25 W) and the residues after the phosphorylated ones (Fmoc-Tyr(tBu)-OH), and at 75°C for the other building blocks (5 min., 25 W). Fmoc-His(Trt)-OH was coupled at room temperature for 10 min followed by a 5 min time at 25 W with the maximum temperature set at 47°C. The coupling mixture contained 5 eq. of amino acid (0.2 M in DMF) with activation by 4.5 eq HBTU (0.45 M in DMF) and 10 eq. DiPEA (2 M in NMP). The peptide was cleaved for 2 hours using the cocktail TFA:TIS:H_2O:EDT (94:1:2.5:2.5 v/v/v/v).

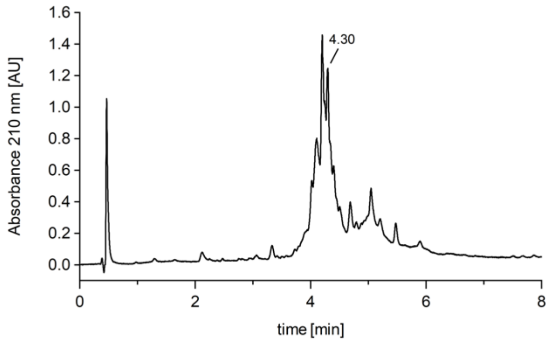

Figure S1 - UPLC analysis of the MW synthesis crude. The desired hexa-phosphorylated peptide 1 elutes at 4.30 minutes. Gradient: 3 → 40 % B1 in 8 min.

5.1.2 **Optimized protocol for SPS of multi-phosphorylated peptides by Fmoc strategy**

All the syntheses were performed in a 10 µmol scale. C-terminal acid peptides were synthesized on the Trt linker (peptide **1**), while C-terminal amide peptides were synthesized on the Rink Amide linker. Before the synthesis, the resin was transferred into a syringe equipped with a filter frit to be weighted and swollen in DMF (30 min). Automated SPPS was carried out by using the following protocol of repeating steps:

- *Deprotection*: removal of the Fmoc-group by treatment of the resin with a solution of 20 % piperidine in DMF (2x5 min for the first 20 residues, 2x10 min for the next ones). Subsequently, the resin was washed with DMF (4x).

- *Coupling*: Fmoc-protected amino acids (8.25 eq.), dissolved in a solution of NMP containing OxymaPure (8 eq.) were activated with HCTU (8 eq. each, c = 0.6 M) and NMM (24 eq., 4 M solution) and transferred to the resin (coupling time 30 min for the first 20 residues, 60 min for subsequent couplings). Phosphorylated building blocks were activated with HOBt and DiPEA, instead of Oxyma and NMM, with the same conditions except that DiPEA was used as pure compound. Every coupling was repeated once. Subsequently, the resin was washed with DMF (3x).

- *Capping*: The resin was treated with DMF:Ac_2O:lutidine (89:5:6 v/v/v) for 5 min to block unreacted amino-groups. Subsequently, the resin was washed with DMF (3x).

- *TFA Cleavage*: The resin was washed with DCM and dried under a vacuum. Then a mixture of TFA:phenol:H_2O:EDT (87.5:5:5:2.5 v/v/v/v, 3 mL/10 µmol peptide) was added to the resin. After 2 h the cleavage cocktail was collected by filtration, the resin was washed with TFA (3x 0.5 mL) and the combined filtrates were concentrated (~ 1 mL) under argon flow. Et_2O (~ 8-10-fold volume) was added to the remaining residue, the suspension was cooled (in dry ice for ~30 min) and centrifuged (4000 rpm, 15 min). Afterwards, the ether phase was decanted. The remaining peptide pellet was dissolved in H_2O:ACN:TFA (2:1:0.001 v/v/v) and freeze-dried.

HHHHHH-Y**pS**PTSPS-Y**pS**PTSPS-Y**pS**PTSPS-Y**pS**PTSPS-Y**pS**PTSPS-Y**pS**PTSPS-COOH, **1**

Yield: 4.46 µmol, 18%.
MW: 5638.0444 g·mol^{-1} ($C_{228}H_{320}N_{60}O_{97}P_6$).
UPLC-MS: t_R = 5.43 min (3 → 40 % B1 in 8 min); m/z = 1880.5889 ($C_{228}H_{323}N_{60}O_{97}P_6$ $(M+3H)^{3+}$, calcd.: 1880.3560), 1410.7828 ($C_{228}H_{324}N_{60}O_{97}P_6$ $(M+4H)^{4+}$, calcd.: 1419.5189), 1128.8268 ($C_{228}H_{325}N_{60}O_{97}P_6$ $(M+5H)^{5+}$, calcd.: 1128.6167).

5.1.3 Synthesis of hexaphosphorylated peptides 2, 3, 4, 5 and unphosphorylated peptide 6

The hexaphosphopeptides were prepared according to the optimized method (4.1.2). Prior to detachment, the peptides on the resin were acetylated on the N-terminus using the reported capping protocol. After TFA cleavage, the crude peptides were purified with RP-HPLC (gradient: 3 → 40 % B1 in 30 min) and the pure fractions were collected for analyses. The UPLC analyses showed the presence of side peaks with m/z-ratios of the main products. This occurred for all the phosphorylated peptides of the series. The side peaks were non-separable, even with an additional RP-HPLC purification. The area under side peaks was reduced upon incubation of the mixture in buffer (NaH_2PO_4 200 mM, pH 7.4).

Ac-Y**pS**PTSPS-Y**pS**PTSPS-Y**pS**PTSPS-Y**pS**PTSPS-Y**pS**PTSPS-Y**pS**PTSPS-NH_2, **2**

Yield: 0.88 µmol, 9%.
MW: 4857.4 g·mol^{-1} ($C_{194}H_{281}N_{43}O_{91}P_6$).
UPLC-MS: t_R = 5.28 min (3 → 40 % B1 in 8 min); m/z = 1215.4 ($C_{194}H_{285}N_{43}O_{91}P_6$ $(M+4H)^{4+}$, calcd.: 1215.4), 972.6 ($C_{194}H_{286}N_{43}O_{91}P_6$ $(M+5H)^{5+}$, calcd.: 972.5).

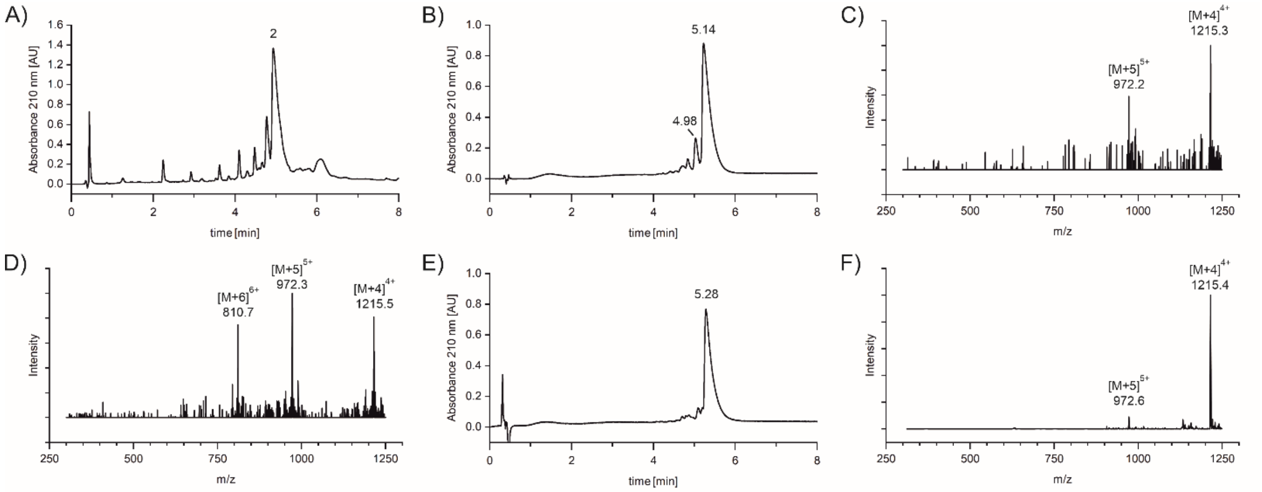

Figure S2 – UPLC-MS analysis of hexaphosphopeptide 2. A) Crude form, B) after HPLC purification, C) mass spectrum of the peak at t_R = 4.98 min, D) mass spectrum of the peak at t_R = 5.14 min, E) after incubation in 200 mM NaH_2PO_4 buffer at pH 7.4., F) mass spectrum of the main peak after pH 7.4 incubation.

Ac-YSP**pT**SPS-YSP**pT**SPS-YSP**pT**SPS-YSP**pT**SPS-YSP**pT**SPS-YSP**pT**SPS-NH_2, **3**

Yield: 0.88 µmol, 9%.
MW: 4857.4 g·mol^{-1} ($C_{194}H_{281}N_{43}O_{91}P_6$).
UPLC-MS: t_R = 5.15 min (3 → 40 % B1 in 8 min); *m/z* = 1215.3 ($C_{194}H_{285}N_{43}O_{91}P_6$ $(M+4H)^{4+}$, calcd.: 1215.4), 972.6 ($C_{194}H_{286}N_{43}O_{91}P_6$ $(M+5H)^{5+}$, calcd.: 972.5).

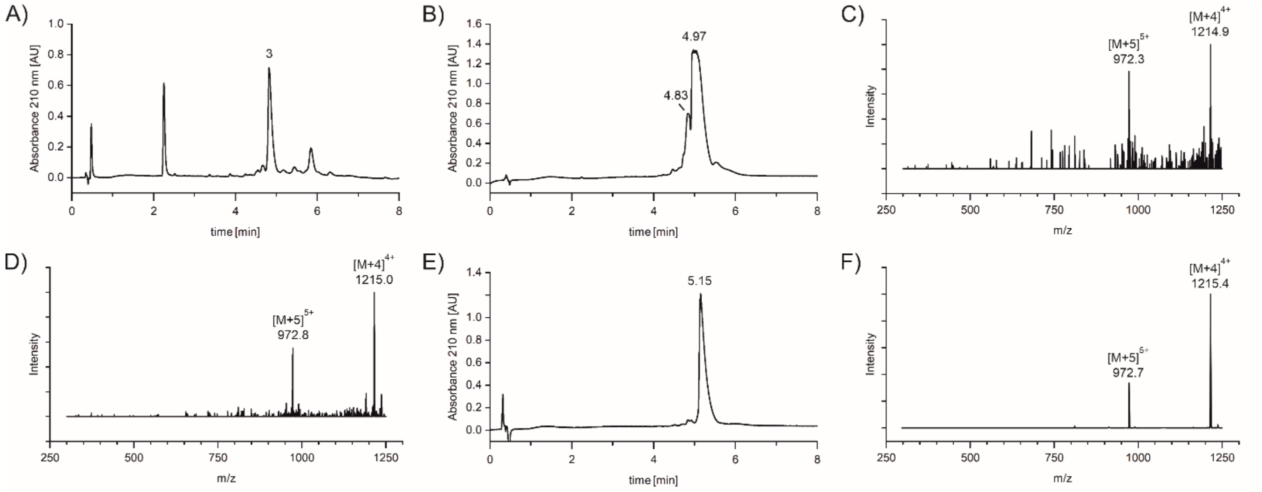

Figure S3 - UPLC-MS analysis of hexaphosphopeptide 2. A) Crude form, B) after HPLC purification, C) mass spectrum of the peak at t_R = 4.83 min, D) mass spectrum of the peak t_R = 4.97 min, E) after incubation in 200 mM NaH_2PO_4 buffer at pH 7.4., F) mass spectrum of the main peak after pH 7.4 incubation.

Ac-YSPT**pS**PS-YSPT**pS**PS-YSPT**pS**PS-YSPT**pS**PS-YSPT**pS**PS-YSPT**pS**PS-NH_2, **4**

Yield: 0.87 µmol, 9%.
MW: 4857.4 g·mol^{-1} ($C_{194}H_{281}N_{43}O_{91}P_6$).
UPLC-MS: t_R = 5.43 min (3 → 40 % B1 in 8 min); *m/z* = 1215.3 ($C_{194}H_{285}N_{43}O_{91}P_6$ $(M+4H)^{4+}$, calcd.: 1215.4), 972.6 ($C_{194}H_{286}N_{43}O_{91}P_6$ $(M+5H)^{5+}$, calcd.: 972.5).

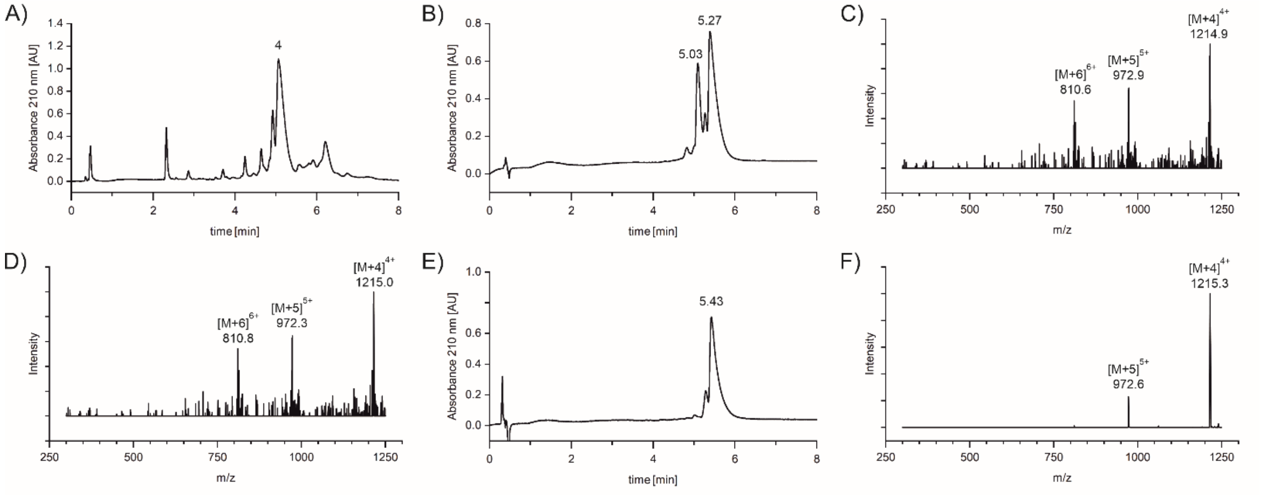

Figure S4 - UPLC-MS analysis of hexaphosphopeptide 2. A) Crude form, B) after HPLC purification, C) mass spectrum of the peak at t_R = 5.03 min, D) mass spectrum of the peak t_R = 5.27 min, E) after incubation in 200 mM NaH_2PO_4 buffer at pH 7.4., F) mass spectrum of the main peak after pH 7.4 incubation.

Ac-YSPTSP**pS**-YSPTSP**pS**-YSPTSP**pS**-YSPTSP**pS**-YSPTSP**pS**-YSPTSP**pS**-NH_2, **5**

Yield: 1.23 µmol, 12%.
MW: 4857.4 g·mol^{-1} ($C_{194}H_{281}N_{43}O_{91}P_6$).
UPLC-MS: t_R = 5.30 min (3 → 40 % B1 in 8 min); *m/z* = 1215.3 ($C_{194}H_{285}N_{43}O_{91}P_6$ $(M+4H)^{4+}$, calcd.: 1215.4), 972.6 ($C_{194}H_{286}N_{43}O_{91}P_6$ $(M+5H)^{5+}$, calcd.: 972.7).

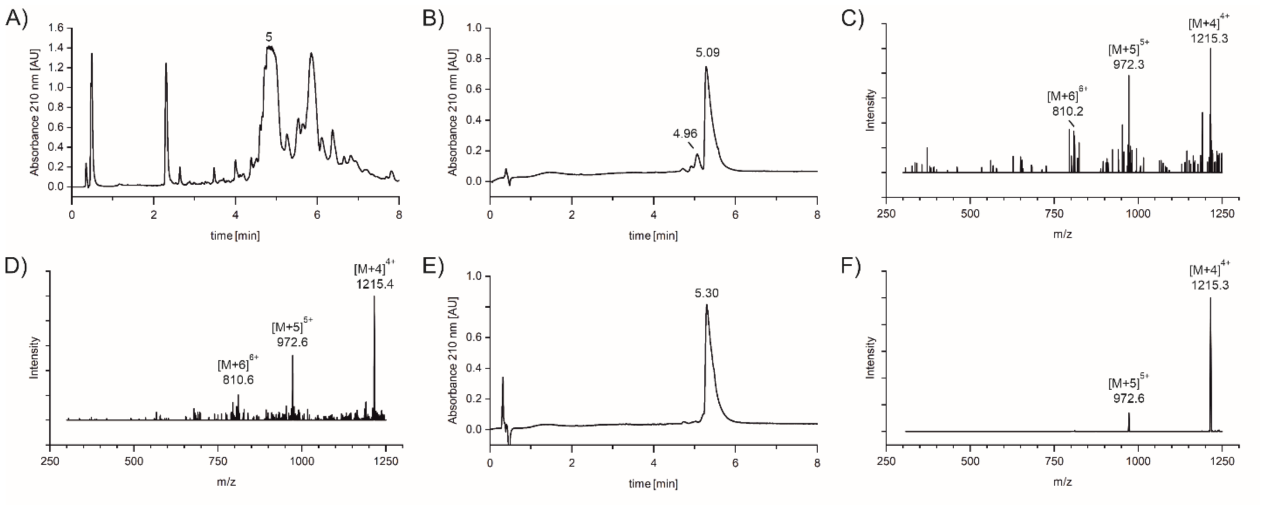

Figure S5 - UPLC-MS analysis of hexaphosphopeptide 2. A) Crude form, B) after HPLC purification, C) mass spectrum of the peak at t_R = 4.96 min, D) mass spectrum of the peak t_R = 5.09 min, E) after incubation in 200 mM NaH_2PO_4 buffer at pH 7.4., F) mass spectrum of the main peak after pH 7.4 incubation.

Ac-YSPTSPS-YSPTSPS-YSPTSPS-YSPTSPS-YSPTSPS-YSPTSPS-NH_2, **6**

Yield: 0.39 µmol, 4%.
MW: 4377.5 g·mol^{-1} ($C_{194}H_{275}N_{43}O_{73}$).
UPLC-MS: t_R = 4.87 min (3 → 40 % B1 in 8 min); *m/z* = 1095.5 ($C_{194}H_{279}N_{43}O_{73}$ $(M+4H)^{4+}$, calcd.: 1095.4), 876.8 ($C_{194}H_{280}N_{43}O_{73}$ $(M+5H)^{5+}$, calcd.: 876.5).

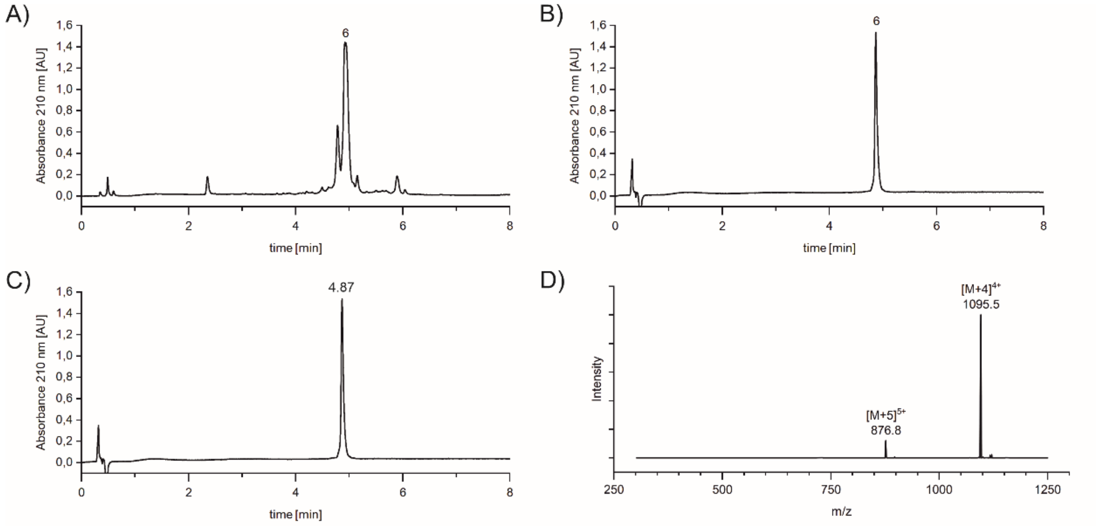

Figure S6 - UPLC-MS analysis of hexaphosphopeptide 2. A) Crude form, B) after HPLC purification, C) after incubation in 200 mM NaH_2PO_4 buffer at pH 7.4., D) mass spectrum of the main peak after pH 7.4 incubation.

5.2 Synthesis of phosphopeptides with affinity purification

The assembly of the CTD core phosphopeptides was carried out according to the optimized method (4.1.2). Subsequently, the N-terminal Fmoc group was removed with piperidine in 20% DMF. Next, the PEG spacer Fmoc-HN-PEG_6-COOH (5.25 eq.) was coupled manually with pre-activation for 5 minutes with HATU (5 eq., 0.5M) and DiPEA (15 eq.). Coupling was performed overnight. For the PEGylation of hexaphosphorylated peptide 8.25 eq. of Fmoc-HN-PEG_6-COOH, 8 eq. of HATU and 24 Eq. of DiPEA were used. The resin was washed (3x) with DMF. Elongation with the coupling of 6 histidines (HisTag) and detachment was performed according to the procedure described in 4.1.2.

The agarose Ni^{2+}-NTA resin was placed in a fritted syringe (2 ml of slurry, 1 ml of resin). The resin was washed 5 times with immobilization buffer (NaH_2PO_4 200 mM, pH 7.4) and allowed to swell for 30 minutes. The lyophilized peptide crude was dissolved in 2 ml of immobilization buffer and transferred to the resin. After one hour of incubation, the syringe was drained and the resin was washed (10x, 2 ml) with immobilization buffer. The His_6-tagged peptide was eluted upon incubating the resin with a solution of 0.25 M of acetic acid (10x, 2 ml for 5 minutes). Product-containing fractions were collected and lyophilized. Figure S7 shows UPLC analyses of samples withdrawn at different stages of the affinity purification process.

HHHHHH-NH-$(CH_2)_2$-$(OCH_2CH_2)_6$-CO-Y**pS**PTSPS-Y**pS**PTSPS-Y**pS**PTSPS-Y**pS**PTSPS-Y**pS**PTSPS-Y**pS**PTSPS-NH_2, **7**

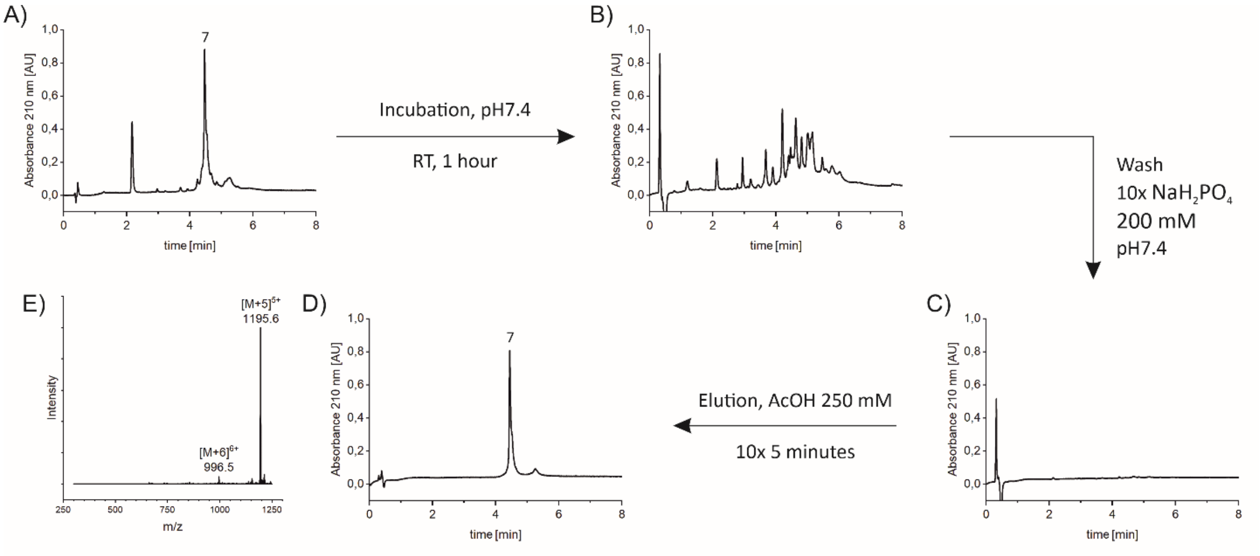

Figure S7 – Nickel-HisTag affinity purification. UPLC-MS analysis of A) crude hexaphosphopeptide **7** after TFA cleavage, B) the solution after incubation with Ni^{2+}-NTA agarose beads, C) filtrates obtained through washing of the peptide-loaded beads and D) **7** after elution with 0.25 M AcOH and lyophilization. E) mass spectrum of the main peak in the chromatogram in picture D.

5.3 Synthesis of dodecaheptad CTD peptides

(I). N-terminal fragments

Code	*Sequence*	*Notes*
8	HHHHHH-PEG$_6$-YSPTSPS-YSPTSPS-YSPTSPS-YSPTSPS-YSPTSPS-YSPTSPS-Y-NHNH$_2$	Hydrazides
9	HHHHHH-PEG$_6$-YSPTSPS-Y**S**PTSPS-YSPTSPS-YSPTSPS-YSPTSPS-YSPTSPS-Y-NHNH$_2$	
10	HHHHHH-PEG$_6$-YSPTSPS-YSPTSPS-YSPTSPS-YSPTSPS-YSPTSPS-YSPTSPS-Y-MPA	MPA Thioesters
11	HHHHHH-PEG$_6$-YSPTSPS-Y**S**PTSPS-YSPTSPS-YSPTSPS-YSPTSPS-YSPTSPS-Y-MPA	

(II). C-terminal fragments

Code	*Sequence*	*Notes*
12	CPTSPS-Y**S**PTSPS-YSPTSPS-YSPTSPS-YSPTSPS-YSPTSPS	CT amide
13	CPTSPS-YSPTSPS-Y**S**PTSPS-YSPTSPS-YSPTSPS-YSPTSPS	
14	CPTSPS-YSPTSPS-YSPTSPS-Y**S**PTSPS-YSPTSPS-YSPTSPS	
15	CPTSPS-YSPTSPS-YSPTSPS-YSPTSPS-Y**S**PTSPS-YSPTSPS	
16	CPTSPS-YSPTSPS-YSPTSPS-YSPTSPS-YSPTSPS-Y**S**PTSPS	

(III). Ligation products (cysteine peptides)

Code	*Sequence*	*Notes*
17	HHHHHH-PEG$_6$-(YSPTSPS)$_6$-Y**C**PTSPS-Y**S**PTSPS-(YSPTSPS)$_4$	10 + 12 , H8
18	HHHHHH-PEG$_6$-(YSPTSPS)$_6$-Y**C**PTSPS-(YSPTSPS)$_4$-Y**S**PTSPS	10 + 16, H12
19	HHHHHH-PEG$_6$-YSPTSPS-Y**S**PTSPS-(YSPTSPS)$_4$-Y**C**PTSPS-Y**S**PTSPS-(YSPTSPS)$_4$	11 + 12, H2/8
20	HHHHHH-PEG$_6$-YSPTSPS-Y**S**PTSPS-(YSPTSPS)$_4$-Y**C**PTSPS-(YSPTSPS)-Y**S**PTSPS-(YSPTSPS)$_3$	11 + 13, H2/9
21	HHHHHH-PEG$_6$-YSPTSPS-Y**S**PTSPS-(YSPTSPS)$_4$-Y**C**PTSPS-(YSPTSPS)$_2$-Y**S**PTSPS-(YSPTSPS)$_2$	11 + 14, H2/10
22	HHHHHH-PEG$_6$-YSPTSPS-Y**S**PTSPS-(YSPTSPS)$_4$-Y**C**PTSPS-(YSPTSPS)$_3$-Y**S**PTSPS-YSPTSPS	11 + 15, H2/11
23	HHHHHH-PEG$_6$-YSPTSPS-Y**S**PTSPS-(YSPTSPS)$_4$-Y**C**PTSPS-(YSPTSPS)$_4$-Y**S**PTSPS	11 + 16, H2/12

(IV). Desulfurization products (alanine peptides)

Code	*Sequence*	*Yield*
24	HHHHHH-PEG$_6$-(YSPTSPS)$_6$-Y**A**PTSPS-Y**S**PTSPS-(YSPTSPS)$_4$	10 %
25	HHHHHH-PEG$_6$-(YSPTSPS)$_6$-Y**A**PTSPS-(YSPTSPS)$_4$-Y**S**PTSPS	50 %
26	HHHHHH-PEG$_6$-YSPTSPS-Y**S**PTSPS-(YSPTSPS)$_4$-Y**A**PTSPS-Y**S**PTSPS-(YSPTSPS)$_4$	33 %
27	HHHHHH-PEG$_6$-YSPTSPS-Y**S**PTSPS-(YSPTSPS)$_4$-Y**A**PTSPS-(YSPTSPS)-Y**S**PTSPS-(YSPTSPS)$_3$	18 %
28	HHHHHH-PEG$_6$-YSPTSPS-Y**S**PTSPS-(YSPTSPS)$_4$-Y**A**PTSPS-(YSPTSPS)$_2$-Y**S**PTSPS-(YSPTSPS)$_2$	26 %
29	HHHHHH-PEG$_6$-YSPTSPS-Y**S**PTSPS-(YSPTSPS)$_4$-Y**A**PTSPS-(YSPTSPS)$_3$-Y**S**PTSPS-YSPTSPS	22 %
30	HHHHHH-PEG$_6$-YSPTSPS-Y**S**PTSPS-(YSPTSPS)$_4$-Y**A**PTSPS-(YSPTSPS)$_4$-Y**S**PTSPS	16 %

Table S2 – Overview of fragments, ligation and desulfurization products used for the synthesis of dodecaheptad peptides. In the third column are reported the yields of the full synthesis calculated on the nmol of MPA thioester used for the NCL step. Letters in red mark the position of phosphorylation, in blue the cysteine mutations and in green the alanine mutations. PEG$_6$ = NH-$CH_2CH_2(OCH_2CH_2)_6$-CO

5.3.1 Fmoc-Tyr(tBu)-hydrazide resin

9-H-Fluoren-9-ylmethyl carbazate (Fmoc-$NHNH_2$) was synthesized as described by Boeglin et al.[2] Trt-Cl resin was allowed to swell in dry DCM for 30 minutes at RT and then treated twice (30 min) with a solution of 5% $SOCl_2$ in dry DCM (3 ml/100 µmol of theoretical active sites) to regenerate possibly hydrolyzed reactive sites. The resin was washed 5 times with dry DCM and then treated with a suspension of the synthesized Fmoc-$NHNH_2$ (5 Eq.) in DMF:DCM 1:1 (4 ml/100 µmol of theoretical active sites), for 1 hour. The treatment was repeated and the resin washed 5 times with DCM and 5 times with DMF.[3] Capping of the remaining chloride active sites with DMF/MeOH/DiPEA (80:15:5, 3 ml/100 µmol of theoretical active sites), twice for 5 minutes. Washing 5 times with DMF. Swelling of the resin for 30 minutes in DMF. Deprotection with 20% piperidine in DMF, twice for 5 minutes. Washing 5 times with DMF and 5 times with 2-MeTHF. Swelling in 2-MeTHF for 30 minutes. Preparation of the coupling solution with Fmoc-Tyr(tBu)-OH (50 Eq.), Oxyma (50 Eq.) and DIC (50 Eq., 0,5 M) in 2-MeTHF. We found that loading hydrazide resins provides higher yields and is more reproducible when 2-MeTHF is used as a solvent instead of DMF.[4] After 5 minutes of activation, the precipitate was removed by centrifugation. The supernatant was transferred to the fritted syringe with the resin for the coupling (twice for 10 minutes). The resin was washed with DMF 5 times and then capped with the standard capping solution for 10 minutes. After both steps, the yield was estimated by monitoring the Fmoc cleavage. The loading of the first amino acid (Fmoc-Tyr(tBu)) proceeded with 80 % yield on average.

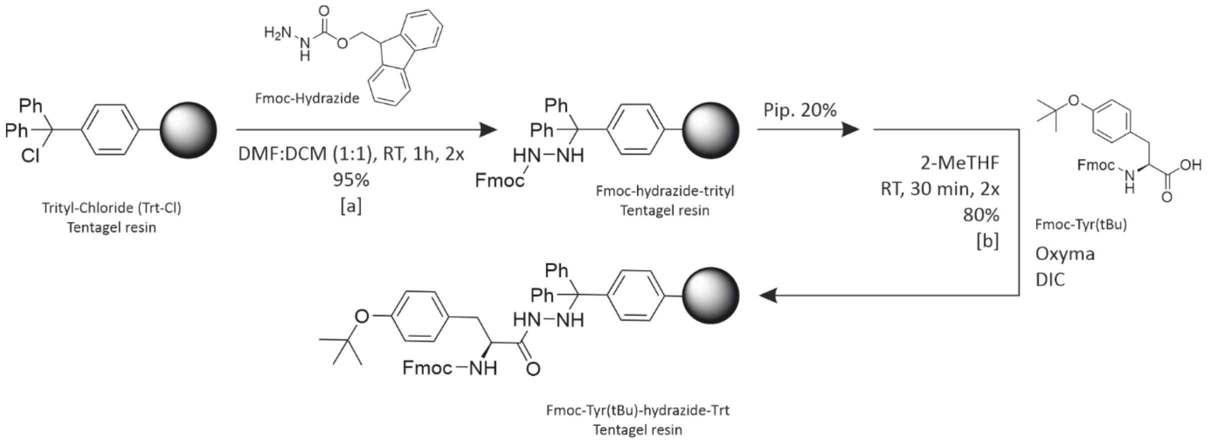

Figure S8 – Scheme of the Fmoc-Tyr(tBu)-hydrazide resin preparation.

5.3.2 Synthesis of the N-terminal fragment thioesters

The His-tagged N-terminal peptides (**8** and **9**, see Table S2) were prepared on the functionalized Fmoc-Tyr-hydrazide Trt resin according to the method described in 4.1.2. After cleavage, the hydrazide peptides were purified through RP-HPLC. The hydrazide fragment was dissolved at 1 mM in 6 M Gnd HCl at pH 3. To this solution, acetylacetone (AcAc, 15 Eq., as a solution 10% in water) and 3-mercaptopropanoic acid (MPA, 200 eq.) were added. After 30 minutes, the pH was carefully raised to 7.5 with 5 M NaOH. The reaction was monitored until formation of the MPA thioester was completed (generally after 1 hour). The resulting peptide thioesters (peptides **9** and **10**) were purified by RP-HPLC. Figure S9 shows UPLC analyses of samples obtained at different stages.

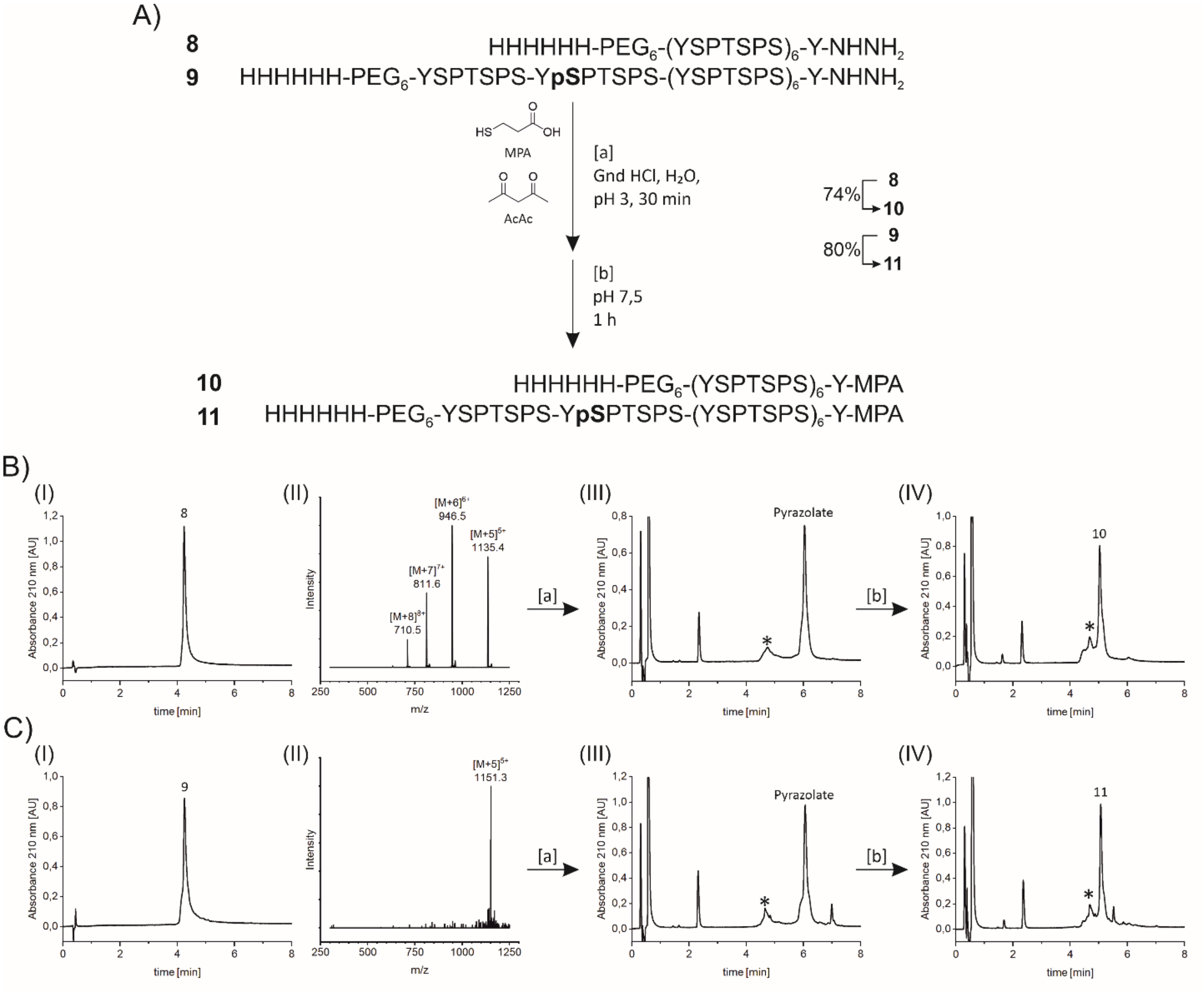

Figure S9 – Thioesterification of hydrazide peptides. (A) Reaction scheme, yields are calculated by integrating the area of the peaks in the chromatograms. In the sequences. UPLC analysis of products obtained at different stages of the synthesis for (B) conversion of peptide-hydrazide **8** to peptide thioester **10** and (C) conversion of phosphopeptide-hydrazide **9** to phosphopeptide thioester **11**. Asterisks mark peaks related to peptide acids formed upon the hydrolysis of peptide thioester and pyrazolates.

HHHHHH-PEG$_6$-YSPTSPS-YSPTSPS-YSPTSPS-YSPTSPS-YSPTSPS-YSPTSPS-Y-NHNH$_2$, **8**

Yield: 2.52 µmol, 25%.
MW: 5670.5 g·mol^{-1} ($C_{252}H_{354}N_{64}O_{87}$)
UPLC-MS: t_R = 4.24 min (10 → 30 % B1 in 8 min); *m/z* = 1135.4 ($C_{252}H_{359}N_{64}O_{87}$ $(M+5H)^{5+}$, calcd.: 1135.1), 946.5 ($C_{252}H_{360}N_{64}O_{87}$ $(M+6H)^{6+}$, calcd.: 946.1), 811.6 ($C_{252}H_{361}N_{64}O_{87}$ $(M+7H)^{7+}$, calcd.: 811.1), 710.5 ($C_{252}H_{362}N_{64}O_{87}$ $(M+8H)^{8+}$, calcd.: 709.8).

HHHHHH-PEG$_6$-YSPTSPS-Y**pS**PTSPS-YSPTSPS-YSPTSPS-YSPTSPS-YSPTSPS-Y-NHNH$_2$, **9**

Yield: 1.85 µmol, 19%.
MW: 5750.5 g·mol^{-1} ($C_{252}H_{355}N_{64}O_{90}P$)
UPLC-MS: t_R = 4.25 min (10 → 30 % B1 in 8 min); *m/z* = 1151.3 ($C_{252}H_{360}N_{64}O_{90}P$ $(M+5H)^{5+}$, calcd.: 1151.4).

HHHHHH-PEG$_6$-YSPTSPS-YSPTSPS-YSPTSPS-YSPTSPS-YSPTSPS-YSPTSPS-Y-MPA, **10**

Yield: 1.76 µmol, 18%.
MW: 5744.5 g·mol^{-1} ($C_{255}H_{356}N_{62}O_{89}S$)
UPLC-MS: t_R = 5.01 min (10 → 30 % B1 in 8 min); *m/z* = 1150.1 ($C_{255}H_{361}N_{62}O_{89}S$ $(M+5H)^{5+}$, calcd.: 1149.9)

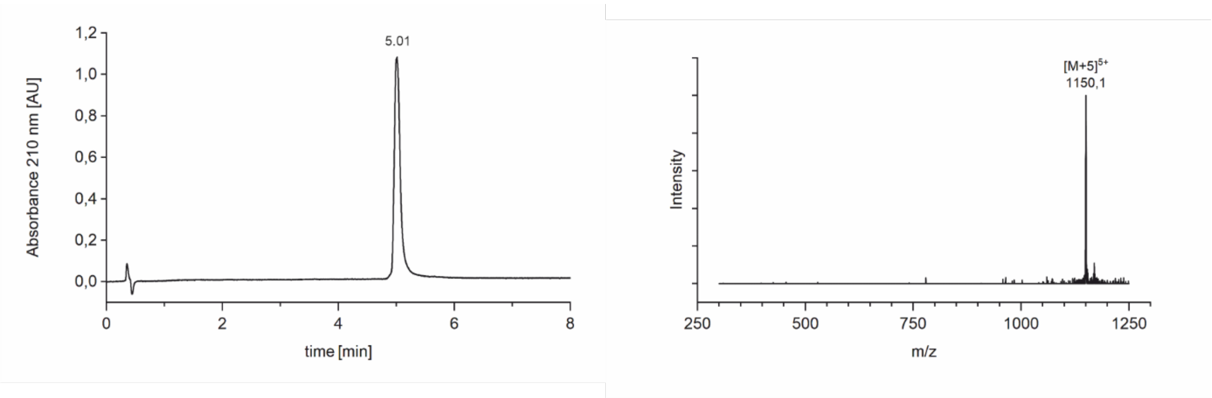

HHHHHH-PEG$_6$-YSPTSPS-Y**pS**PTSPS-YSPTSPS-YSPTSPS-YSPTSPS-YSPTSPS-Y-MPA, **11**

Yield: 1.22 µmol, 12%.
MW: 5824.5 g·mol^{-1} ($C_{255}H_{355}N_{62}O_{92}PS$)
UPLC-MS: t_R = 5.04 min (10 → 30 % B1 in 8 min); *m/z* = 1165.8 ($C_{255}H_{360}N_{62}O_{92}PS$ $(M+5H)^{5+}$, calcd.: 1165.9).

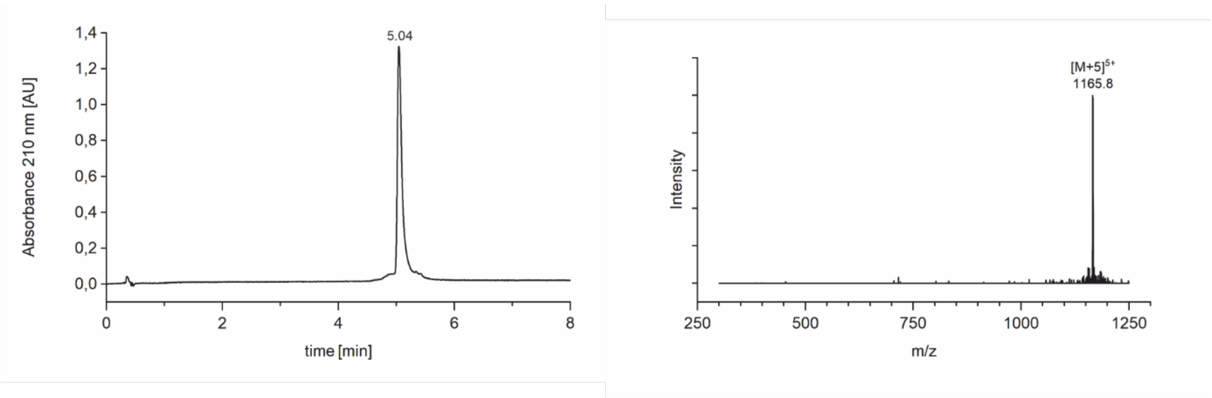

Figure S10 – Analysis and characterization of the MPA thioesters. The percentage yields of the pure thioester are calculated on the theoretical full scale of the synthesis.

5.3.3 Synthesis of C-terminal fragments

C-terminal fragments (**12**-**16**, see Table S2) for native chemical ligation were assembled on RinkAmide resin as described in section 4.1.2 and purified by RP-HPLC.

CPTSPS-Y**pS**PTSPS-YSPTSPS-YSPTSPS-YSPTSPS-YSPTSPS-YSPTSPS-NH_2, **12**

Yield: 4.80 µmol, 48%.
MW: 4268.3 g·mol^{-1} ($C_{183}H_{265}N_{42}O_{72}PS$)
UPLC-MS: t_R = 4.17 min (10 → 30 % B1 in 8 min); *m/z* = 1068.2 ($C_{183}H_{269}N_{42}O_{72}PS$ $(M+4H)^{4+}$, calcd.: 1068.1), 855.0 ($C_{183}H_{270}N_{42}O_{72}PS$ $(M+5H)^{5+}$, calcd.: 854.7).

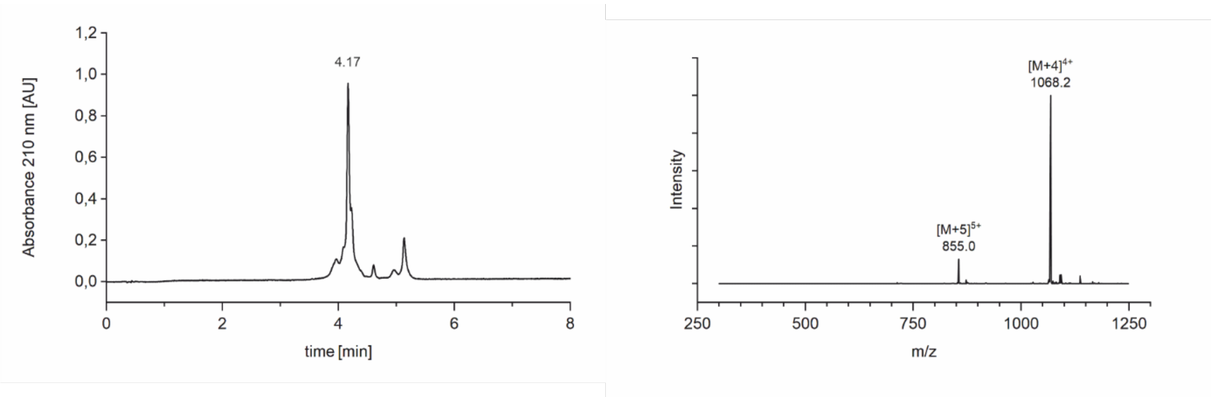

CPTSPS-YSPTSPS-Y**pS**PTSPS-YSPTSPS-YSPTSPS-YSPTSPS-NH_2, **13**
Yield: 4.44 µmol, 44%.
MW: 4268.3 g·mol^{-1} ($C_{183}H_{265}N_{42}O_{72}PS$)
UPLC-MS: t_R = 4.15 min (10 → 30 % B1 in 8 min); *m/z* = 1068.2 ($C_{183}H_{269}N_{42}O_{72}PS$ $(M+4H)^{4+}$, calcd.: 1068.1), 855.0 ($C_{183}H_{270}N_{42}O_{72}PS$ $(M+5H)^{5+}$, calcd.: 854.7).

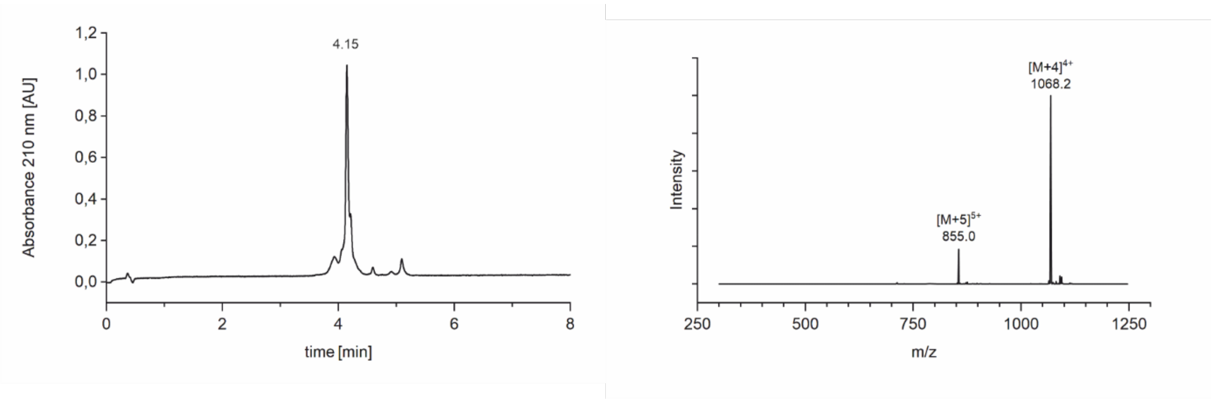

CPTSPS-YSPTSPS-YSPTSPS-Y**pS**PTSPS-YSPTSPS-YSPTSPS-NH_2, **14**

Yield: 3.68 µmol, 37%.
MW: 4268.3 g·mol^{-1} ($C_{183}H_{265}N_{42}O_{72}PS$)
UPLC-MS: t_R = 4.15 min (10 → 30 % B1 in 8 min); *m/z* = 1068.2 ($C_{183}H_{269}N_{42}O_{72}PS$ $(M+4H)^{4+}$, calcd.: 1068.1), 855.0 ($C_{183}H_{270}N_{42}O_{72}PS$ $(M+5H)^{5+}$, calcd.: 854.7).

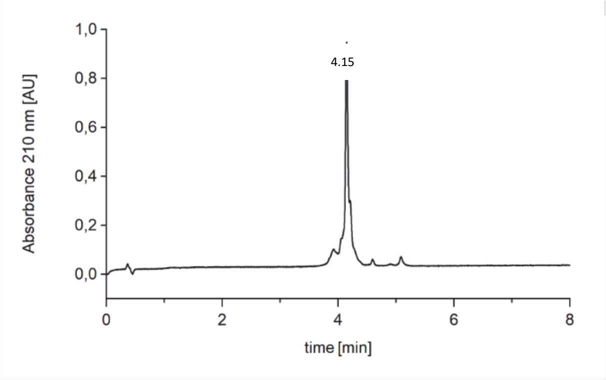

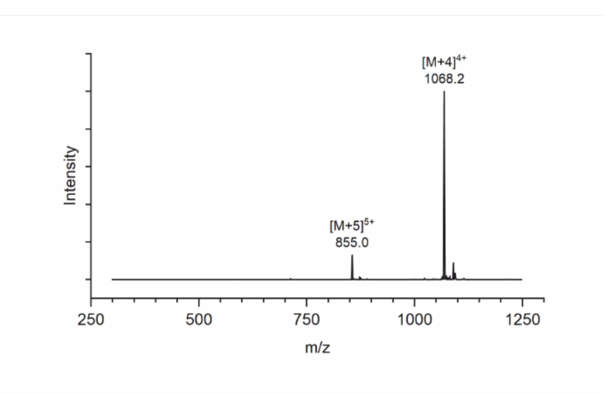

CPTSPS-YSPTSPS-YSPTSPS-YSPTSPS-Y**pS**PTSPS-YSPTSPS-NH_2, **15**

Yield: 4.44 µmol, 44%.
MW: 4268.3 g·mol^{-1} ($C_{183}H_{265}N_{42}O_{72}PS$)
UPLC-MS: t_R = 4.13 min (10 → 30 % B1 in 8 min); *m/z* = 1068.2 ($C_{183}H_{269}N_{42}O_{72}PS$ $(M+4H)^{4+}$, calcd.: 1068.1), 854.9 ($C_{183}H_{270}N_{42}O_{72}PS$ $(M+5H)^{5+}$, calcd.: 854.7).

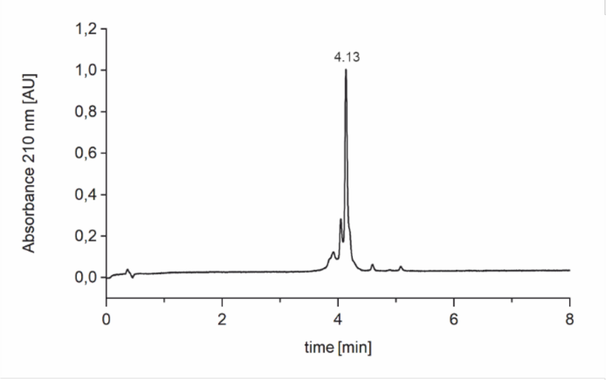

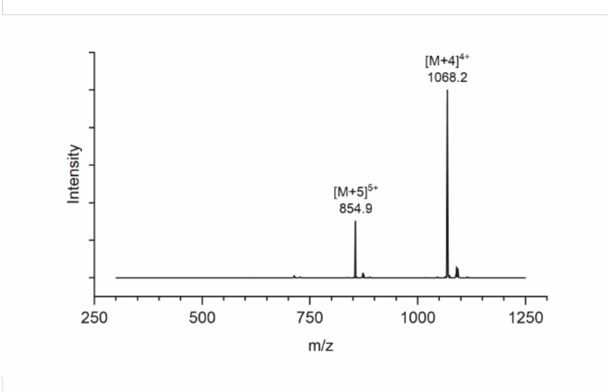

CPTSPS-YSPTSPS-YSPTSPS-YSPTSPS-YSPTSPS-Y**pS**PTSPS-NH_2, **16**
Yield: 3.63 µmol, 36%.
MW: 4268.3 g·mol^{-1} ($C_{183}H_{265}N_{42}O_{72}PS$)
UPLC-MS: t_R = 4.15 min (10 → 30 % B1 in 8 min); *m/z* = 1068.2 ($C_{183}H_{269}N_{42}O_{72}PS$ $(M+4H)^{4+}$, calcd.: 1068.1), 855.0 ($C_{183}H_{270}N_{42}O_{72}PS$ $(M+5H)^{5+}$, calcd.: 854.7).

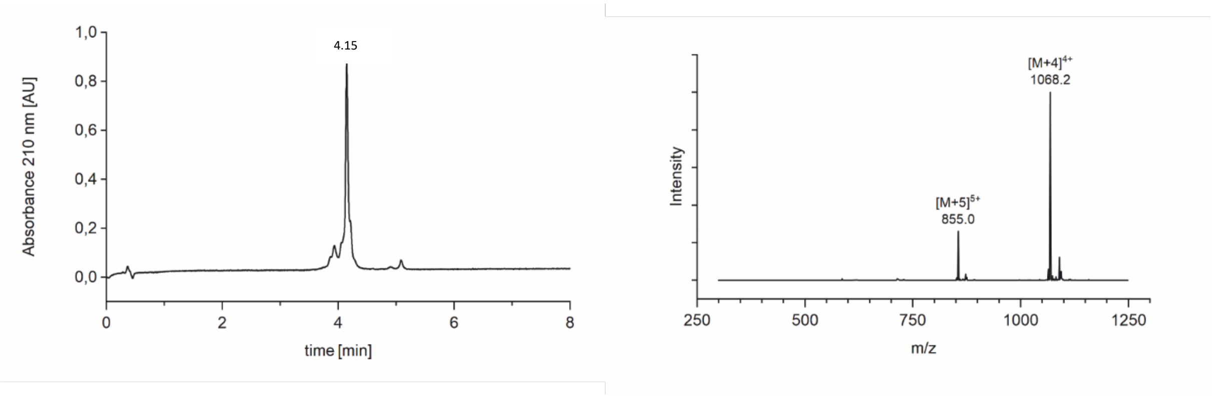

Figure S11 – Analysis and characterization of the C-terminal fragments. The percentage yields of the pure C-terminal fragments are calculated on the theoretical full scale of the synthesis.

5.3.4 Native Chemical Ligation (NCL)

The N-terminal fragment and the C-terminal fragment were co-lyophilized in a 1:1.25 molar ratio. The dried mixture was dissolved (10 mM) in the ligation buffer (6M Gnd HCl, 200 mM Na_2HPO_4, 200 mM MPAA, 50 mM TCEP HCl at pH 7). After 4 hours under argon atmosphere and room temperature, the mixture was diluted 1:10 with water. Small molecules (in particular MPAA) were separated by using size exclusion Sephadex G-25 columns (dry particle size: 20-80 µm, fractionation range for globular proteins: 1000-5000 Da) according to the protocol provided by the manufacturer. Product-containing fractions were collected and lyophilized.

A)

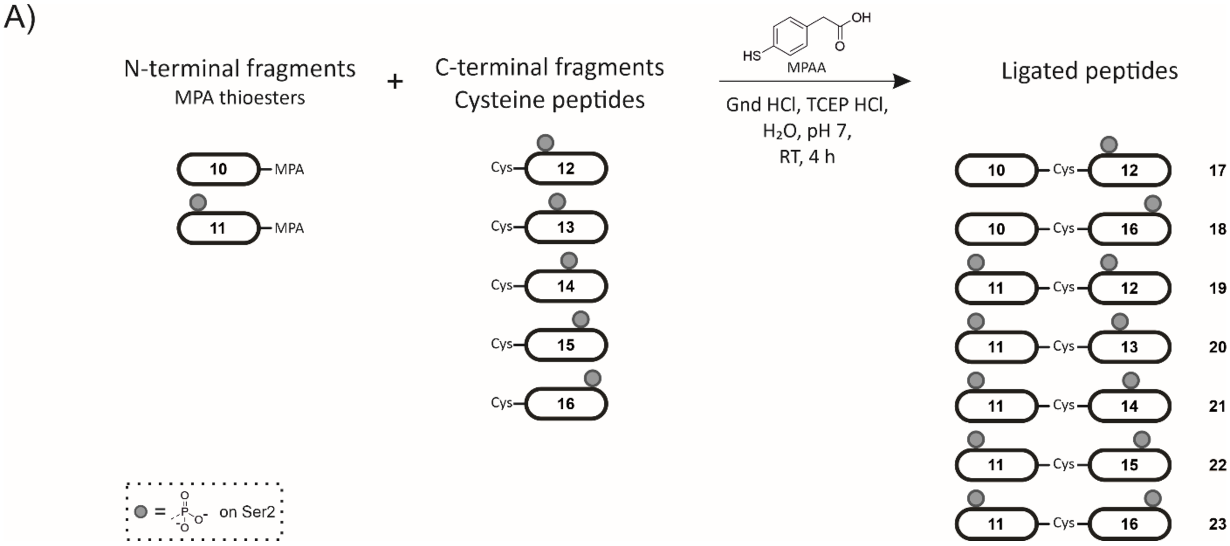

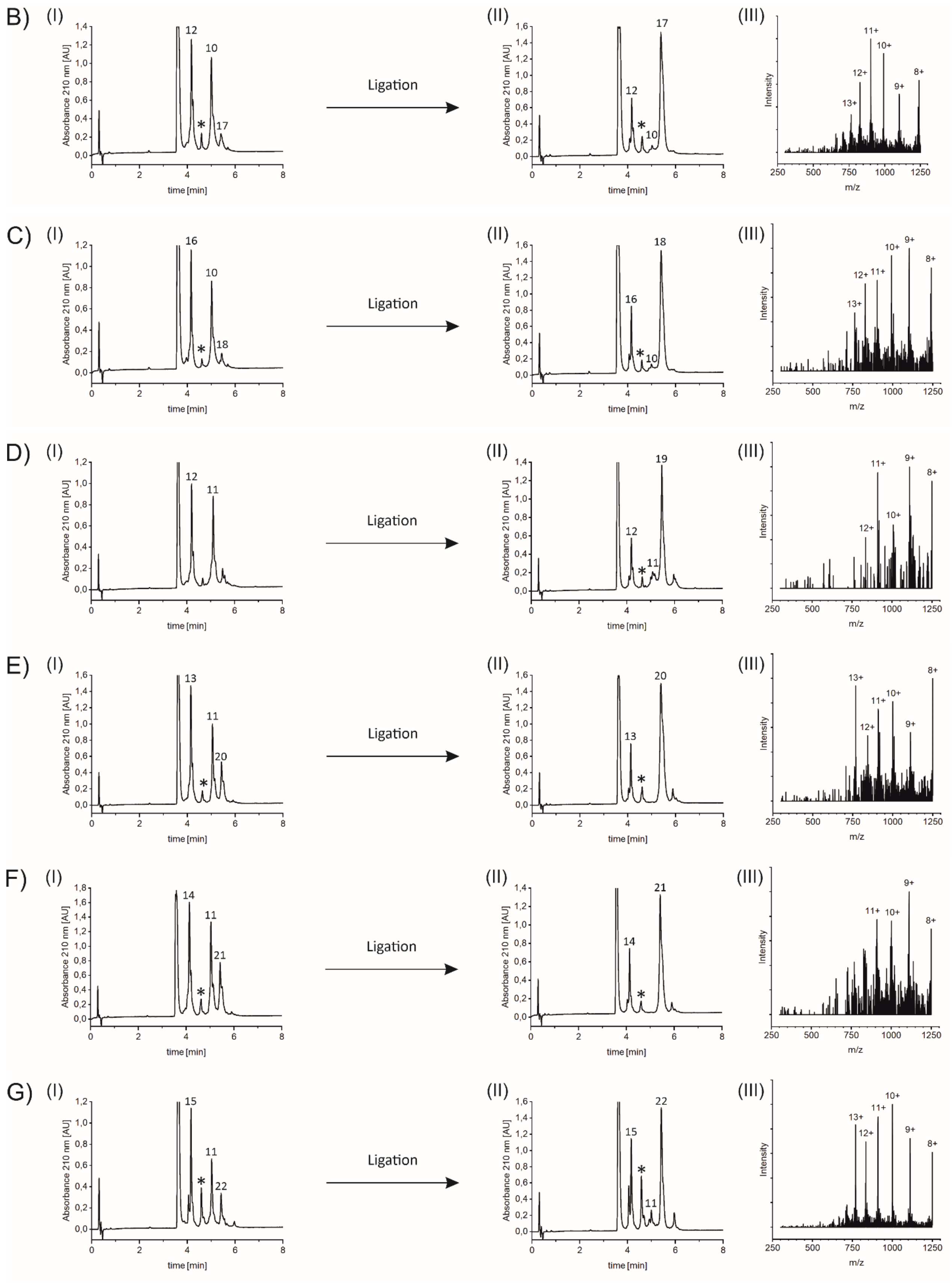
B) (I)
12
10
*
17
Ligation
(II)
17
12
*
10
(III)
11+
10+
12+
8+
9+
13+
C) (I)
16
10
*
18
Ligation
(II)
18
16
*
10
(III)
9+
10+
8+
12+ 11+
13+
D) (I)
12
11
Ligation
(II)
19
12
11
*
(III)
9+
11+
8+
10+
12+
E) (I)
13
11
20
*
Ligation
(II)
20
13
*
(III)
8+
13+
10+
11+
12+
9+
F) (I)
14
11
21
*
Ligation
(II)
21
14
*
(III)
9+
11+ 10+
8+
G) (I)
15
11
*
22
Ligation
(II)
22
15
*
11
(III)
10+
11+
13+
12+
9+
8+
Absorbance 210 nm [AU]
time [min]
Intensity
m/z

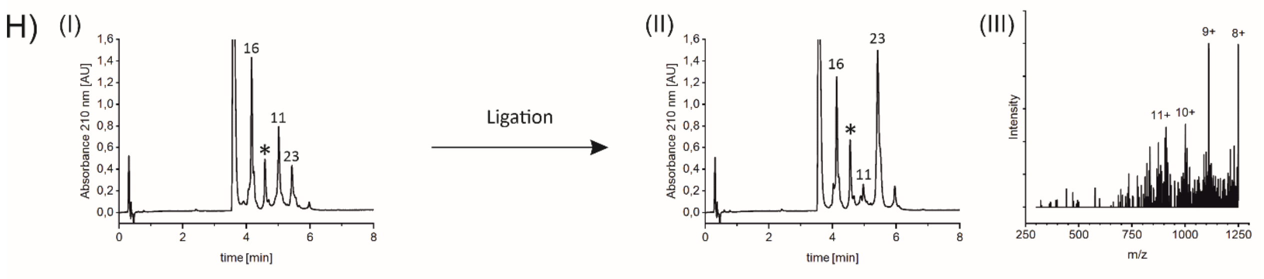

Figure S12 – Native chemical ligation. (A) Overview of native chemical ligation reactions. (B-H) UPLC-MS analysis of ligation mixtures at t=0 min (left) and t = 4 h (right). In all reactions, the thioester peptide (**10** or **11**) is almost fully consumed by the ligation or the hydrolysis. The C-terminal fragments are not fully consumed because they were added in excess. The peak eluting at 3.8 minutes represents the MPAA in the ligation mixtures. The peaks marked with the asterisks are related to the acid peptides due to hydrolysis of the thioesters. Please note, direct injection from ligation reactions resulted in noisy mass spectra.

HHHHHH-PEG6-YSPTSPS-YSPTSPS-YSPTSPS-YSPTSPS-YSPTSPS-YSPTSPS-Y<u>C</u>PTSPS-Y**pS**PTSPS-YSPTSPS-YSPTSPS-YSPTSPS-YSPTSPS-YSPTSPS-NH_2, **17**

MW: 9907.3 g·mol^{-1} ($C_{435}H_{615}N_{104}O_{159}PS$).
UPLC-MS: t_R = 5.38 min (10 → 30 % B1 in 8 min); m/z = 1239.7 ($C_{435}H_{623}N_{104}O_{159}PS$ $(M+8H)^{8+}$, calcd.: 1239.4), 1101.5 ($C_{435}H_{624}N_{104}O_{159}PS$ $(M+9H)^{9+}$, calcd.: 1101.8), 992.0 ($C_{435}H_{625}N_{104}O_{159}PS$ $(M+10H)^{10+}$, calcd.: 991.7), 902.0 ($C_{435}H_{626}N_{104}O_{159}PS$ $(M+11H)^{11+}$, calcd.: 901.7), 829.9 ($C_{435}H_{627}N_{104}O_{159}PS$ $(M+12H)^{12+}$, calcd.: 826.6), 763.5 ($C_{435}H_{628}N_{104}O_{159}PS$ $(M+13H)^{13+}$, calcd.: 763.1)

HHHHHH-PEG_6-YSPTSPS-YSPTSPS-YSPTSPS-YSPTSPS-YSPTSPS-YSPTSPS-Y<u>C</u>PTSPS-YSPTSPS-YSPTSPS-YSPTSPS-YSPTSPS-Y**pS**PTSPS-NH_2, **18**

MW: 9907.3 g·mol^{-1} ($C_{435}H_{615}N_{104}O_{159}PS$).
UPLC-MS: t_R = 5.39 min (10 → 30 % B1 in 8 min); m/z = 1239.5 ($C_{435}H_{623}N_{104}O_{159}PS$ $(M+8H)^{8+}$, calcd.: 1239.4), 1101.9 ($C_{435}H_{624}N_{104}O_{159}PS$ $(M+9H)^{9+}$, calcd.: 1101.8), 991.8 ($C_{435}H_{625}N_{104}O_{159}PS$ $(M+10H)^{10+}$, calcd.: 991.7), 901.7 ($C_{435}H_{626}N_{104}O_{159}PS$ $(M+11H)^{11+}$, calcd.: 901.7), 826.8 ($C_{435}H_{627}N_{104}O_{159}PS$ $(M+12H)^{12+}$, calcd.: 826.6), 761.8 ($C_{435}H_{628}N_{104}O_{159}PS$ $(M+13H)^{13+}$, calcd.: 763.1)

HHHHHH-PEG_6-YSPTSPS-Y**pS**PTSPS-YSPTSPS-YSPTSPS-YSPTSPS-YSPTSPS-Y<u>C</u>PTSPS-Y**pS**PTSPS-YSPTSPS-YSPTSPS-YSPTSPS-YSPTSPS-NH_2, **19**

MW: 9987.3 g·mol^{-1} ($C_{435}H_{616}N_{104}O_{162}P_2S$).
UPLC-MS: t_R = 5.46 min (10 → 30 % B1 in 8 min); m/z = 1249.3 ($C_{435}H_{624}N_{104}O_{162}P_2S$ $(M+8H)^{8+}$, calcd.: 1249.4), 1110.7 ($C_{435}H_{625}N_{104}O_{162}P_2S$ $(M+9H)^{9+}$, calcd.: 956.6), 1006.3 ($C_{435}H_{626}N_{104}O_{162}P_2S$ $(M+10H)^{10+}$, calcd.: 999.7), 910.0 ($C_{435}H_{627}N_{104}O_{162}P_2S$ $(M+11H)^{11+}$, calcd.: 908.9), 833.0 ($C_{435}H_{628}N_{104}O_{162}P_2S$ $(M+12H)^{12+}$, calcd.: 833.3).

HHHHHH-PEG$_6$-YSPTSPS-Y**pS**PTSPS-YSPTSPS-YSPTSPS-YSPTSPS-YSPTSPS-YC̲PTSPS-YSPTSPS-Y**pS**PTSPS-YSPTSPS-YSPTSPS-YSPTSPS-NH$_2$, **20**

MW: 9987.3 g·mol^{-1} ($C_{435}H_{616}N_{104}O_{162}P_2S$).
UPLC-MS: t_R = 5.38 min (10 → 30 % B1 in 8 min); m/z = 1249.4 ($C_{435}H_{624}N_{104}O_{162}P_2S$ $(M+8H)^{8+}$, calcd.: 1249.4), 1110.5 ($C_{435}H_{625}N_{104}O_{162}P_2S$ $(M+9H)^{9+}$, calcd.: 956.6), 1000.1 ($C_{435}H_{626}N_{104}O_{162}P_2S$ $(M+10H)^{10+}$, calcd.: 999.7), 908.7 ($C_{435}H_{627}N_{104}O_{162}P_2S$ $(M+11H)^{11+}$, calcd.: 908.9), 831.6 ($C_{435}H_{628}N_{104}O_{162}P_2S$ $(M+12H)^{12+}$, calcd.: 833.3), 768.0 ($C_{435}H_{629}N_{104}O_{162}P_2S$ $(M+13H)^{13+}$, calcd.: 769.3).

HHHHHH-PEG$_6$-YSPTSPS-Y**pS**PTSPS-YSPTSPS-YSPTSPS-YSPTSPS-YSPTSPS-YC̲PTSPS-YSPTSPS-YSPTSPS-Y**pS**PTSPS-YSPTSPS-YSPTSPS-NH$_2$, **21**

MW: 9987.3 g·mol^{-1} ($C_{435}H_{616}N_{104}O_{162}P_2S$).
UPLC-MS: t_R = 5.40 min (10 → 30 % B1 in 8 min); m/z = 1249.9 ($C_{435}H_{624}N_{104}O_{162}P_2S$ $(M+8H)^{8+}$, calcd.: 1249.4), 1110.9 ($C_{435}H_{625}N_{104}O_{162}P_2S$ $(M+9H)^{9+}$, calcd.: 956.6), 1000.2 ($C_{435}H_{626}N_{104}O_{162}P_2S$ $(M+10H)^{10+}$, calcd.: 999.7), 909.5 ($C_{435}H_{627}N_{104}O_{162}P_2S$ $(M+11H)^{11+}$, calcd.: 908.9).

HHHHHH-PEG$_6$-YSPTSPS-Y**pS**PTSPS-YSPTSPS-YSPTSPS-YSPTSPS-YSPTSPS-YC̲PTSPS-YSPTSPS-YSPTSPS-YSPTSPS-Y**pS**PTSPS-YSPTSPS-NH$_2$, **22**

MW: 9987.3 g·mol^{-1} ($C_{435}H_{616}N_{104}O_{162}P_2S$).
UPLC-MS: t_R = 5.41 min (10 → 30 % B1 in 8 min); m/z = 1249.6 ($C_{435}H_{624}N_{104}O_{162}P_2S$ $(M+8H)^{8+}$, calcd.: 1249.4), 1110.6 ($C_{435}H_{625}N_{104}O_{162}P_2S$ $(M+9H)^{9+}$, calcd.: 956.6), 999.9 ($C_{435}H_{626}N_{104}O_{162}P_2S$ $(M+10H)^{10+}$, calcd.: 999.7), 909.0 ($C_{435}H_{627}N_{104}O_{162}P_2S$ $(M+11H)^{11+}$, calcd.: 908.9), 833.7 ($C_{435}H_{628}N_{104}O_{162}P_2S$ $(M+12H)^{12+}$, calcd.: 833.3), 769.4 ($C_{435}H_{629}N_{104}O_{162}P_2S$ $(M+13H)^{13+}$, calcd.: 769.3).

HHHHHH-PEG$_6$-YSPTSPS-Y**pS**PTSPS-YSPTSPS-YSPTSPS-YSPTSPS-YSPTSPS-YC̲PTSPS-YSPTSPS-YSPTSPS-YSPTSPS-YSPTSPS-Y**pS**PTSPS-NH$_2$, **23**

MW: 9987.3 g·mol^{-1} ($C_{435}H_{616}N_{104}O_{162}P_2S$).
UPLC-MS: t_R = 5.42 min (10 → 30 % B1 in 8 min); m/z = 1249.5 ($C_{435}H_{624}N_{104}O_{162}P_2S$ $(M+8H)^{8+}$, calcd.: 1249.4), 1110.5 ($C_{435}H_{625}N_{104}O_{162}P_2S$ $(M+9H)^{9+}$, calcd.: 956.6), 999.5 ($C_{435}H_{626}N_{104}O_{162}P_2S$ $(M+10H)^{10+}$, calcd.: 999.7), 908.7 ($C_{435}H_{627}N_{104}O_{162}P_2S$ $(M+11H)^{11+}$, calcd.: 908.9).

5.3.5 Desulfurization

The crude ligation product was dissolved (1 mM) in the desulfurization buffer (6 M Gnd HCl, 0.2 M sodium citrate, pH 4.5). To the solution was added TCEP HCl (200 mM) and subsequently $NaBEt_4$ (100 eq., from a stock solution of 1 g/ml). After 5 minutes, the reaction was completed. The compounds were purified by RP-HPLC.

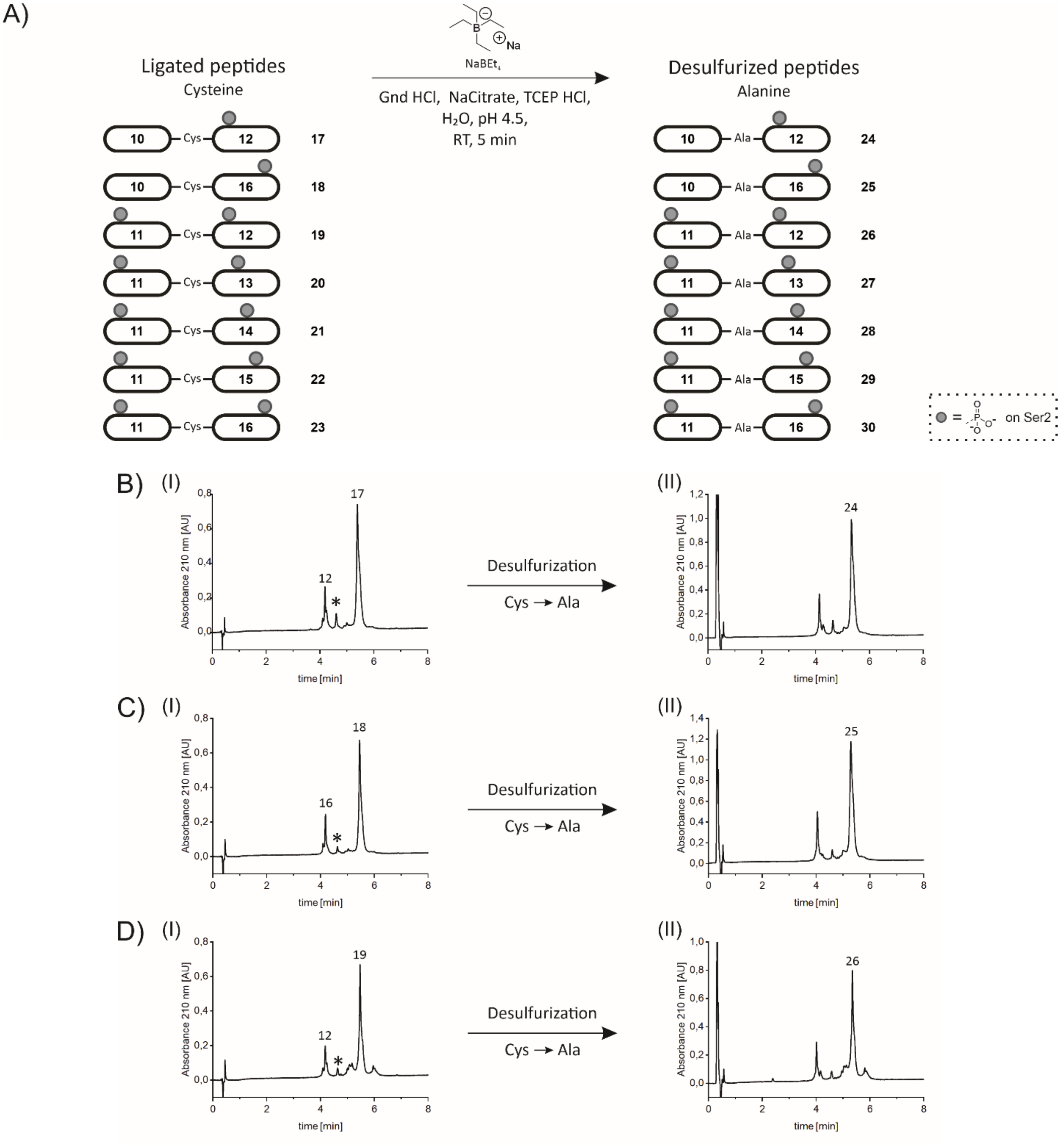

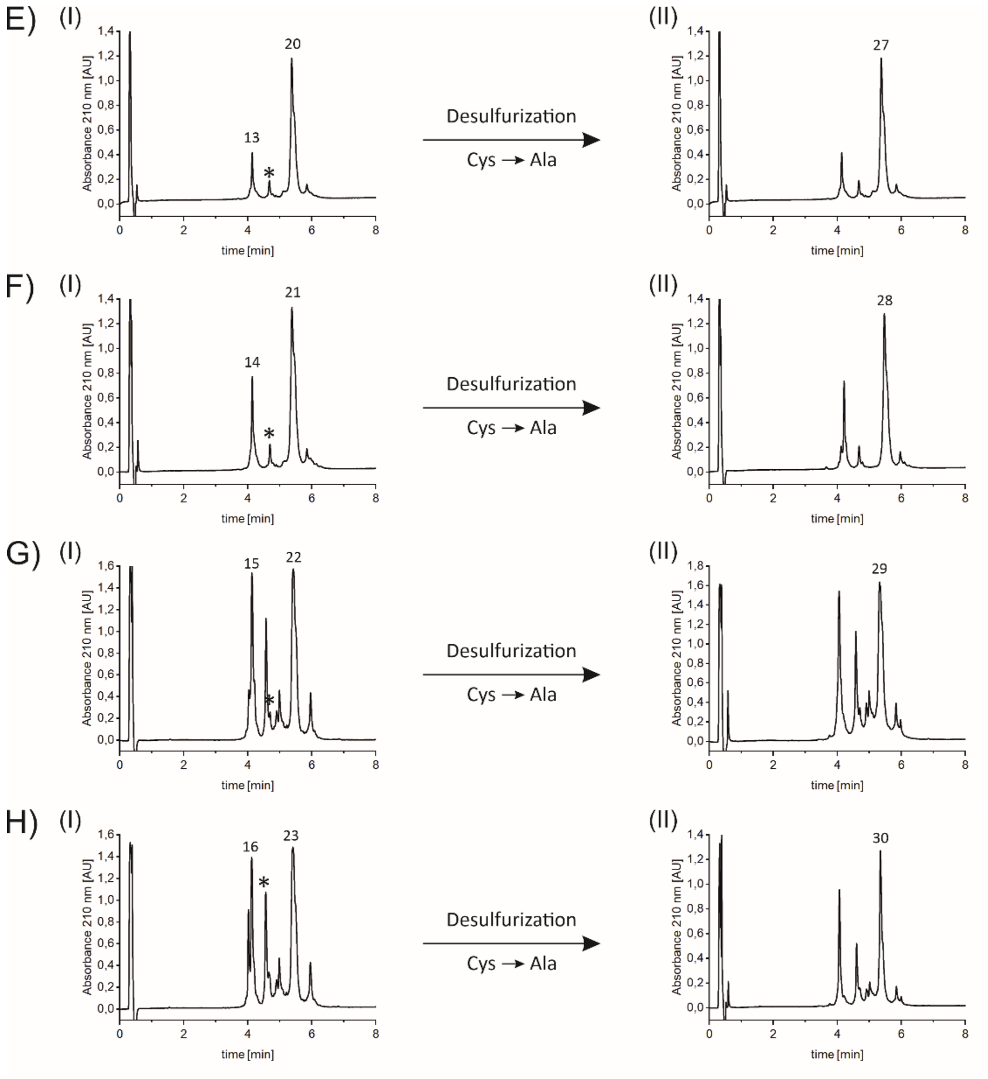

Figure S13 – Desulfurization. (A) Overview of native chemical ligation reactions. (B-H) UPLC-MS analysis of desulfurization reactions at t=0 min (left) and t = 5 min (right). Samples B-D were analyzed before the addition of the desulfurization reagents, and the others after.

HHHHHH-PEG$_6$-YSPTSPS-YSPTSPS-YSPTSPS-YSPTSPS-YSPTSPS-YSPTSPS-Y<u>A</u>PTSPS-Y**pS**PTSPS-YSPTSPS-YSPTSPS-YSPTSPS-YSPTSPS-NH$_2$, **24**

Yield: 95 nmol, 10%.
MW: 9875.3141 g·mol^{-1} ($C_{435}H_{615}N_{104}O_{159}P$).
UPLC-MS: t_R = 5.35 min (10 → 30 % B1 in 8 min); m/z = 1975.9208 ($C_{435}H_{620}N_{104}O_{159}P$ $(M+5H)^{5+}$, calcd.: 1976.0706), 1647.0259 ($C_{435}H_{621}N_{104}O_{159}P$ $(M+6H)^{6+}$, calcd.: 1646.8935), 1411.7666 ($C_{435}H_{622}N_{104}O_{159}P$ $(M+7H)^{7+}$, calcd.: 1411.7670), 1235.5592 ($C_{435}H_{623}N_{104}O_{159}P$ $(M+8H)^{8+}$, calcd.: 1235.4221), 1098.1633 ($C_{435}H_{624}N_{104}O_{159}P$ $(M+9H)^{9+}$, calcd.: 1098.2649).

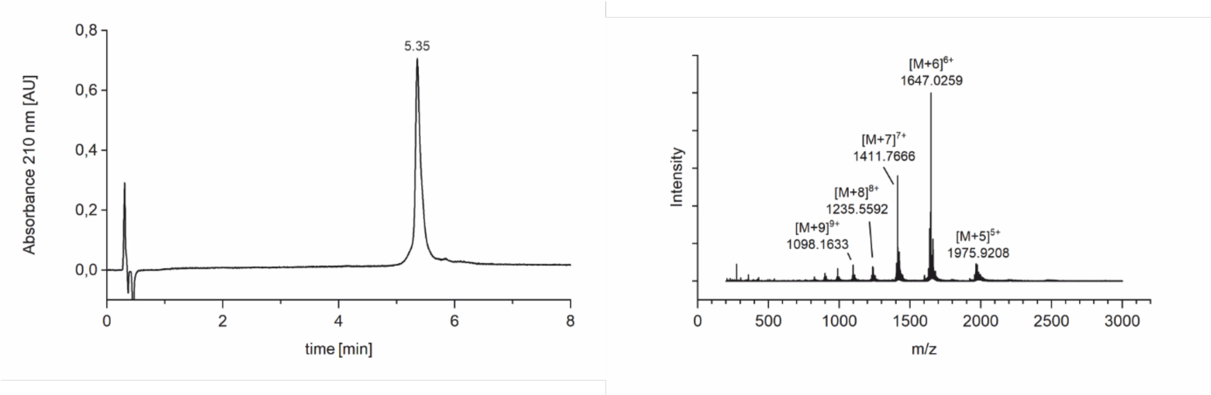

HHHHHH-PEG$_6$-YSPTSPS-YSPTSPS-YSPTSPS-YSPTSPS-YSPTSPS-YSPTSPS-Y<u>A</u>PTSPS-YSPTSPS-YSPTSPS-YSPTSPS-YSPTSPS-Y**pS**PTSPS-NH$_2$, **25**

Yield: 225 nmol, 50%.
MW: 9875.3141 g·mol^{-1} ($C_{435}H_{615}N_{104}O_{159}P$).
UPLC-MS: t_R = 5.39 min (10 → 30 % B1 in 8 min); m/z = 1975.9292 ($C_{435}H_{620}N_{104}O_{159}P$ $(M+5H)^{5+}$, calcd.: 1976.0706), 1647.0259 ($C_{435}H_{621}N_{104}O_{159}P$ $(M+6H)^{6+}$, calcd.: 1646.8935), 1411.7666 ($C_{435}H_{622}N_{104}O_{159}P$ $(M+7H)^{7+}$, calcd.: 1411.7670), 1235.4297 ($C_{435}H_{623}N_{104}O_{159}P$ $(M+8H)^{8+}$, calcd.: 1235.4221), 1098.4888 ($C_{435}H_{624}N_{104}O_{159}P$ $(M+9H)^{9+}$, calcd.: 1098.2649)

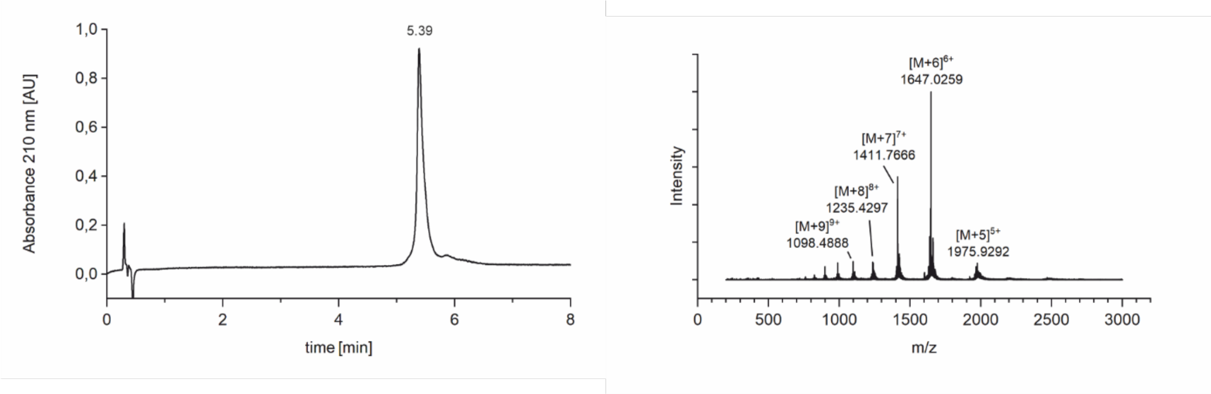

HHHHHH-PEG$_6$-YSPTSPS-Y**pS**PTSPS-YSPTSPS-YSPTSPS-YSPTSPS-YSPTSPS-Y<u>A</u>PTSPS-Y**pS**PTSPS-YSPTSPS-YSPTSPS-YSPTSPS-YSPTSPS-NH$_2$, **26**

Yield: 83 nmol, 33%.
MW: 9955.2804 g·mol^{-1} ($C_{435}H_{616}N_{104}O_{162}P_2$).
UPLC-MS: t_R = 5.45 min (10 → 30 % B1 in 8 min); m/z = 1991.9297 ($C_{435}H_{621}N_{104}O_{162}P_2$ $(M+5H)^{5+}$, calcd.: 1992.0639), 1660.2196 ($C_{435}H_{622}N_{104}O_{162}P_2$ $(M+6H)^{6+}$, calcd.: 1660.2212), 1423.2118 ($C_{435}H_{623}N_{104}O_{162}P_2$ $(M+7H)^{7+}$, calcd.: 1423.1907), 1245.5747 ($C_{435}H_{624}N_{104}O_{162}P_2$ $(M+8H)^{8+}$, calcd.: 1245.4179), 1107.1710 ($C_{435}H_{625}N_{104}O_{162}P_2$ $(M+9H)^{9+}$, calcd.: 1107.1501).

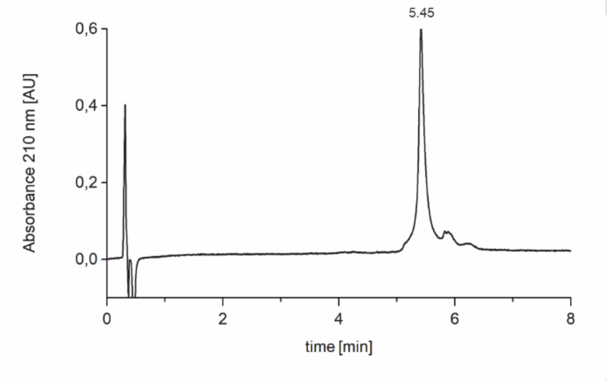

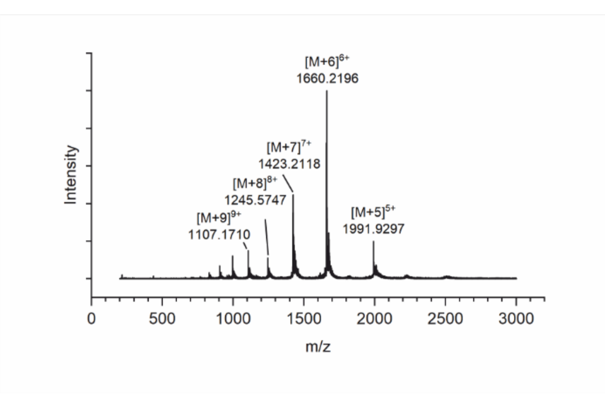

HHHHHH-PEG$_6$-YSPTSPS-Y**pS**PTSPS-YSPTSPS-YSPTSPS-YSPTSPS-YSPTSPS-Y<u>A</u>PTSPS-YSPTSPS-Y**pS**PTSPS-YSPTSPS-YSPTSPS-YSPTSPS-NH$_2$, **27**

Yield: 82 nmol, 18%.
MW: 9955.2804 g·mol^{-1} ($C_{435}H_{616}N_{104}O_{162}P_2$).
UPLC-MS: t_R = 5.45 min (10 → 30 % B1 in 8 min); m/z = 1992.1306 ($C_{435}H_{621}N_{104}O_{162}P_2$ $(M+5H)^{5+}$, calcd.: 1992.0639), 1660.2196 ($C_{435}H_{622}N_{104}O_{162}P_2$ $(M+6H)^{6+}$, calcd.: 1660.2212), 1423.3507 ($C_{435}H_{623}N_{104}O_{162}P_2$ $(M+7H)^{7+}$, calcd.: 1423.1907), 1245.5602 ($C_{435}H_{624}N_{104}O_{162}P_2$ $(M+8H)^{8+}$, calcd.: 1245.4179), 1107.1710 ($C_{435}H_{625}N_{104}O_{162}P_2$ $(M+9H)^{9+}$, calcd.: 1107.1501).

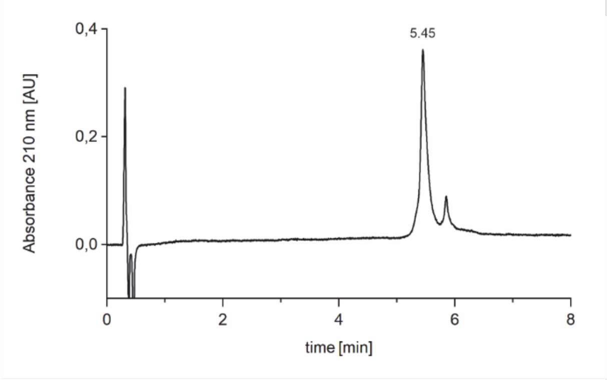

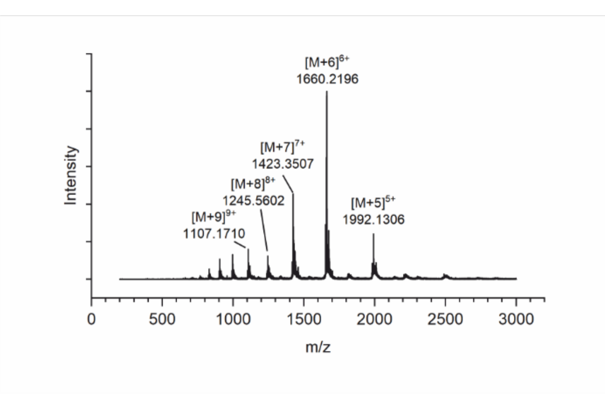

HHHHHH-PEG$_6$-YSPTSPS-Y**pS**PTSPS-YSPTSPS-YSPTSPS-YSPTSPS-YSPTSPS-Y$\underline{A}$PTSPS-YSPTSPS-YSPTSPS-Y**pS**PTSPS-YSPTSPS-YSPTSPS-NH$_2$, **28**

Yield: 117 nmol, 26%.
MW: 9955.2804 g·mol^{-1} ($C_{435}H_{616}N_{104}O_{162}P_2$).
UPLC-MS: t_R = 5.40 min (10 → 30 % B1 in 8 min); m/z = 1991.9115 ($C_{435}H_{621}N_{104}O_{162}P_2$ $(M+5H)^{5+}$, calcd.: 1992.0639), 1660.3696 ($C_{435}H_{622}N_{104}O_{162}P_2$ $(M+6H)^{6+}$, calcd.: 1660.2212), 1423.1694 ($C_{435}H_{623}N_{104}O_{162}P_2$ $(M+7H)^{7+}$, calcd.: 1423.1907), 1245.4303 ($C_{435}H_{624}N_{104}O_{162}P_2$ $(M+8H)^{8+}$, calcd.: 1245.4179), 1107.1710 ($C_{435}H_{625}N_{104}O_{162}P_2$ $(M+9H)^{9+}$, calcd.: 1107.1501).

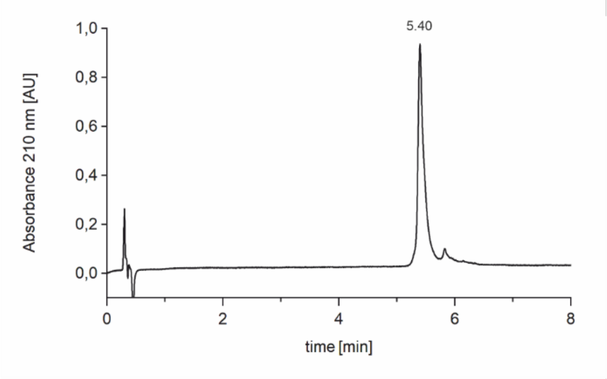

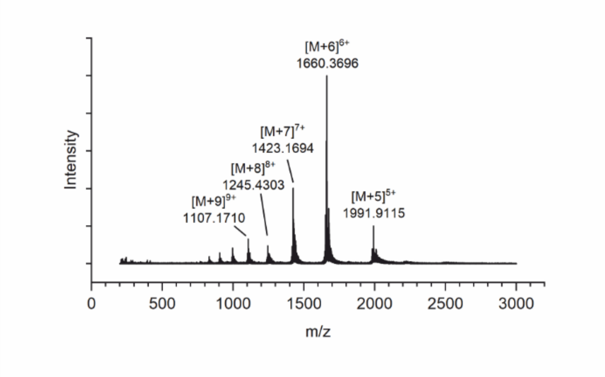

HHHHHH-PEG$_6$-YSPTSPS-Y**pS**PTSPS-YSPTSPS-YSPTSPS-YSPTSPS-YSPTSPS-Y$\underline{A}$PTSPS-YSPTSPS-YSPTSPS-YSPTSPS-Y**pS**PTSPS-YSPTSPS-NH$_2$, **29**

Yield: 89 nmol, 22%.
MW: 9955.2804 g·mol^{-1} ($C_{435}H_{616}N_{104}O_{162}P_2$).
UPLC-MS: t_R = 5.43 min (10 → 30 % B1 in 8 min); m/z = 1991.9480 ($C_{435}H_{621}N_{104}O_{162}P_2$ $(M+5H)^{5+}$, calcd.: 1992.0639), 1660.2196 ($C_{435}H_{622}N_{104}O_{162}P_2$ $(M+6H)^{6+}$, calcd.: 1660.2212), 1423.2118 ($C_{435}H_{623}N_{104}O_{162}P_2$ $(M+7H)^{7+}$, calcd.: 1423.1907), 1245.4303 ($C_{435}H_{624}N_{104}O_{162}P_2$ $(M+8H)^{8+}$, calcd.: 1245.4179), 1107.1710 ($C_{435}H_{625}N_{104}O_{162}P_2$ $(M+9H)^{9+}$, calcd.: 1107.1501).

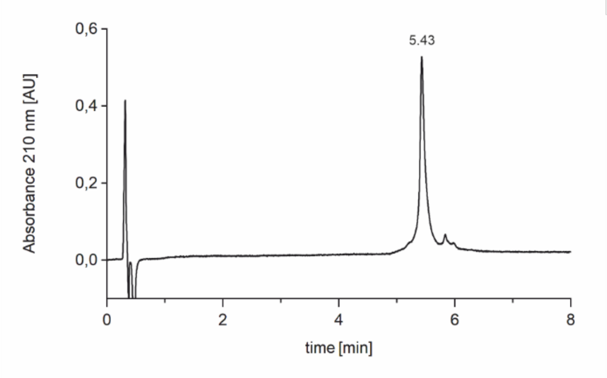

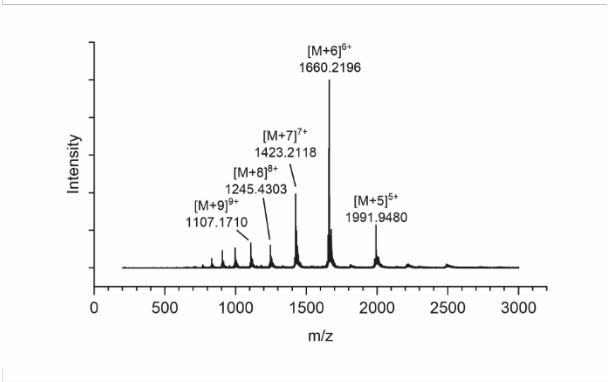

HHHHHH-PEG$_6$-YSPTSPS-Y**pS**PTSPS-YSPTSPS-YSPTSPS-YSPTSPS-YSPTSPS-Y<u>A</u>PTSPS-YSPTSPS-YSPTSPS-YSPTSPS-YSPTSPS-YSPTSPS-Y**pS**PTSPS-NH_2, **30**

Yield: 64 nmol, 16%.
MW: 9955.2804 g·mol^{-1} ($C_{435}H_{616}N_{104}O_{162}P_2$).
UPLC-MS: t_R = 5.41 min (10 → 30 % B1 in 8 min); m/z = 1991.9297 ($C_{435}H_{621}N_{104}O_{162}P_2$ $(M+5H)^{5+}$, calcd.: 1992.0639), 1660.2196 ($C_{435}H_{622}N_{104}O_{162}P_2$ $(M+6H)^{6+}$, calcd.: 1660.2212), 1423.2118 ($C_{435}H_{623}N_{104}O_{162}P_2$ $(M+7H)^{7+}$, calcd.: 1423.1907), 1245.4447 ($C_{435}H_{624}N_{104}O_{162}P_2$ $(M+8H)^{8+}$, calcd.: 1245.4179), 1107.2729 ($C_{435}H_{625}N_{104}O_{162}P_2$ $(M+9H)^{9+}$, calcd.: 1107.1501).

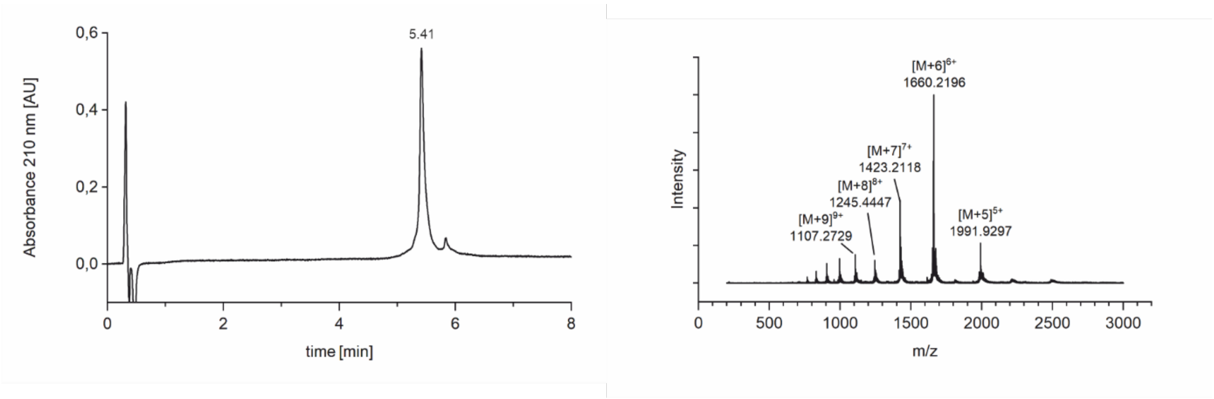

Figure S14 – Analysis and characterization of desulfurized peptides. After desulfurization, the peptides have been purified by RP-HPLC (gradient 10% to 30% of B1) and analysed by UPLC-MS. The yield was determined by OD measurement on the NanoDrop device at 274 nm. The percentage yields of the pure represents the total yield from the NCL to the desulfurization and it is calculated based on the amount of thioester peptide added in the ligation.

5.4 ELISAs

5.4.1 Sample preparation

A stock solution in immobilization buffer (NaH_2PO_4 200 mM, pH 7.4) was prepared for every test peptide. The concentration of the stock was measured with the NanoDrop at 274 nm (Tyr ε_{274} = 1400 $M^{-1}cm^{-1}$). Aliquots were withdrawn and diluted stepwise with immobilization buffer from high µM range to 300 nM. Stock solutions and samples were stored at -80°C.

5.4.2 ELISA protocols

- Thawing of all the samples at room temperature for 30 minutes. The samples were quickly centrifuged and carefully mixed before being used.
- Plating of the samples (100 µl) into a black non-binding 96-well plate that was previously conditioned overnight with wash buffer (NaH_2PO_4 200 mM, BSA 1% w/v, Tween-20 0.05%, pH 7.4) and washed 3 times with the immobilization buffer.
- Samples were transferred with a multi-channel pipette to the clear Ni^{2+}-coated 96-well plate. This plate-to-plate transfer provided full control of the timing of the Ni^{2+}-His_6 interaction.
- Incubation for one hour at 25°C.
- Preparation of the primary antibody dilutions from the stock and then subsequent serial dilutions using wash buffer.
- Washing the plate 5 times for one minute with wash buffer (200 µl).
- Plating of the primary antibody (100 µl) and incubation of the primary antibody for 1 hour at 37°C.
- Washing the plate 5 times for one minute with wash buffer (200 µl).
- Preparation of the dilution and plating (100 µl) of the secondary antibody.
- Incubation for 30 minutes at 37°C.
- Washing the plate 5 times for one minute with wash buffer (200 µl).
- Plating of the freshly prepared TMB-H_2O_2 solution (1:1, 100 µl).
- TMB solution incubation for 20 minutes at 25°C.
- Plating of the Stop solution (2 M H_2SO_4, 100 µL).
- Incubation at 25°C for 1 minute.
- Plate reading at 450 nm, normal aperture, 1 second as measurement time.

All the described ELISAs were performed on fresh plates which had wells dedicated to blanks, and negative controls for the primary and secondary antibodies. The average value of the optical density of the blanks was subtracted from the OD of the analytes in the same plate before data analysis. All the experiments have been performed as biological triplicate unless otherwise stated. The position of the probes, the antibodies and/or the analyte has been changed in each triplicate to provide randomization on the plate.

- Titration curve: 5 dilutions of the primary antibody were used, from 1:250 to 1:4000 of the stock. The OD values were plotted on GraphPad Prism 8 and analysed by fitting a curve with non-linear regression using the model "One site – Specific binding" (Table S3).
- Relative affinity measurements: 3 serial dilutions of the primary mAbs were used in the experiment from 1:1000 to 1:4000 of the stock (E1Z3G: 0.37, 0.19, 0.09 nM, EPR18855: 5.2, 2.6, 1.3 nM and 2G1: 8.3, 4.2, 2.1 nM). The relative binding values were generated by dividing the OD value for each probe by the OD value of the reference peptide tested in the same plate, with the same conditions. Figures 4B, 4C, 6, 7 in the main text show values for the lowest concentrations.
- Competitive ELISA: the reference peptide (**35**) was plated as described. After the incubation time and the wash, the series of dilutions (300, 100, 50, 25, 12.5, 6.25, 2.1 and 0.7 nM) of the competitors was plated, all

the samples in half the volume (50 µL) and double the concentration. Incubation for 5 minutes at 25°C. Plating of the primary antibody in half the volume (50 µL) and double the concentration. The final volume in each well was 100 µL, and the final concentration of the primary antibody was 1:1000. On the same plate, a highly concentrated sample of each competitor (1.2 µM) was also plated to evaluate possible unspecific binding of the competitors to the wells. On the same plate, three wells were excluded from the addition of competitor. The average OD value of these wells was set as 100% binding value. The percentage of the relative binding was plotted on GraphPad Prism 8 and analysed by fitting a curve with non-linear regression using the model "One site -Fit $logIC_{50}$" (Table S4). To use this model, which gives the best fitting, the concentrations of the binders were converted to the log_{10} corresponding value.

- Strong binders (Fig. S16): Two serial dilutions of the primary mAbs (only Abcam and ThermoFisher) were used in the experiment from 1:16000 and 1:64000 of the stock (Ab: 325, 81 pM and TF: 519, 130 pM). The secondary antibody was diluted 1:64000 from the original stock. The dilution series from the stock was done with an intermediate step. The relative binding was generated as described previously. Figure S16 shows values for the highest concentrations.

5.4.3 Additional material ELISA

	One site - Specific binding						
Peptides	***31***	***32***	***33***	***34***	***35***	***36***	***37***
Best-fit values							
Bmax	-0.00512	3.547	3.713	3.729	3.741	3.741	4.659
K_d	-1.769	3256	864.3	935.8	502.3	2106	2070
Std. Error							
K_d	0.03479	0.4975	0.1284	0.1390	0.09727	0.5101	0.3768
Goodness of Fit							
Degrees of Freedom	13	13	13	13	13	13	13
R squared	0.000234	0.9949	0.9771	0.9788	0.9413	0.9769	0.9864
# of points							
# of X values	15	15	15	15	15	15	15
# Y values	15	15	15	15	15	15	15

Table S3 – Titration curve fitting. Data was fit to the model "One site – Specific binding" on GraphPad Prism V8.0.2.

EPR18855			One site - Fit $logIC_{50}$		
Peptides	**2**	**3**	**4**	**5**	**6**
Best-fit values		*Interrupted*		*Interrupted*	
Top	86.89	95.44	92.8	861446	95,98
Bottom	-13.34	-2090150	94.53	94.32	96.63
$LogIC_{50}$	2.323	8.303	1.287	-5.744	2.1
IC_{50}	210.4	2.01E+08	19.38	1.8E-06	125.8
Std. Error					
$LogIC_{50}$	0.1048		2.323		15.38
Goodness of Fit					
Degrees of Freedom	5		5		5
R squared	0.9921		0.1103		0.004039
Number of points					
# of X values	8	8	8	8	8
# Y values analyzed	8	8	8	8	8

2G1			One site - Fit $logIC_{50}$		
Peptides	**2**	**3**	**4**	**5**	**6**
Best-fit values		Ambiguous	Interrupted		
Top	99.68	104.8	102	103.5	104.6
Bottom	-8.142	~ -742461	-220981	94.81	98.33
$LogIC_{50}$	2.145	~ 8.143	7.562	2.609	2.397
IC_{50}	139.5	~ 138916483	36510074	406.1	249.2
Std. Error					
$LogIC_{50}$	0.1712	~ 1802756		2.745	2.981
Goodness of Fit					
Degrees of Freedom	5	5		5	5
R squared	0.972	0.04317		0.2716	0.1535
Number of points					
# of X values	8	8	8	8	8
# Y values analyzed	8	8	8	8	8

E1Z3G	One site - Fit $logIC_{50}$				
Peptides	***2***	***3***	***4***	***5***	***6***
Best-fit values		Interrupted	Ambiguous		
Top	91.89	-3130257	~ -20158	105.8	101
Bottom	0.811	102.2	105.8	85.6	26.57
$LogIC_{50}$	0.5607	-5.866	~ -3.629	2.738	3.338
IC_{50}	3.637	1.36E-06	~ 0.0002351	546.9	2177
Std. Error					
$LogIC_{50}$	0.07684		~ 1406	1.46	6.516
Goodness of Fit					
Degrees of Freedom	5		5	5	5
R squared	0.993		0.7758	0.6639	0.5004
Number of points					
# of X values	8	8	8	8	8
# Y values analyzed	8	8	8	8	8

Table S4 – Competitive ELISA curve fitting. Data was fit to the model “One site – Fit $logIC_{50}$” on GraphPad Prism V8.0.2.

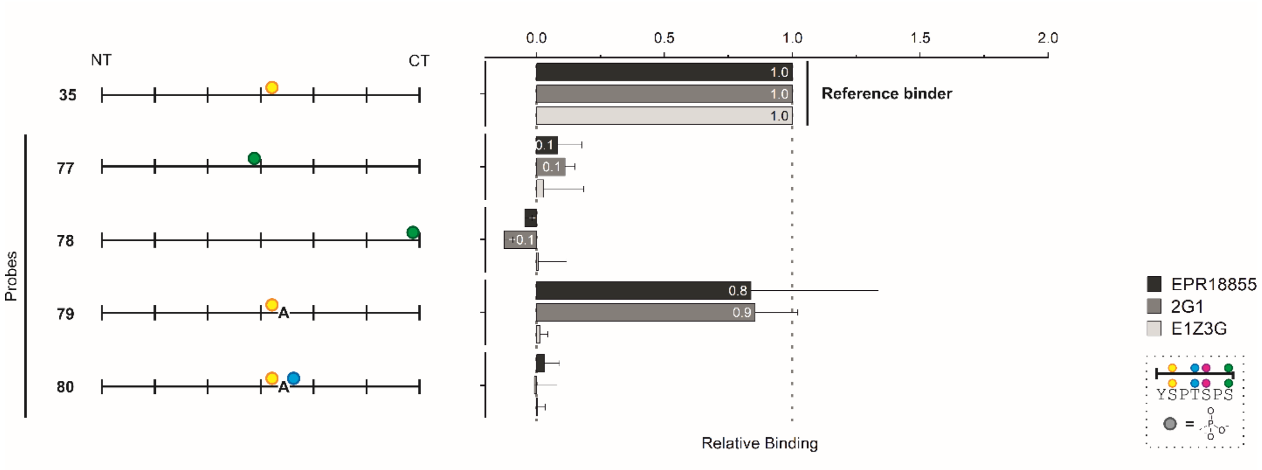

Figure S15 – Additional ELISA data. Assaying peptides **77** and **78** confirmed that in absence of pS2 there is no unspecific binding of the mAbs to pS7. Assaying peptide **79** revealed that mAbs EP18855 and 2G1 tolerate a P3→A3 mutation adjacent to pS2. This mutation is not tolerated by the high affinity antibody E1Z3G. Regardless of the P3→A3 mutation, phosphorylation of T4 in the +2-position of pS2 prevents recognition by EP18855 and 2G1.

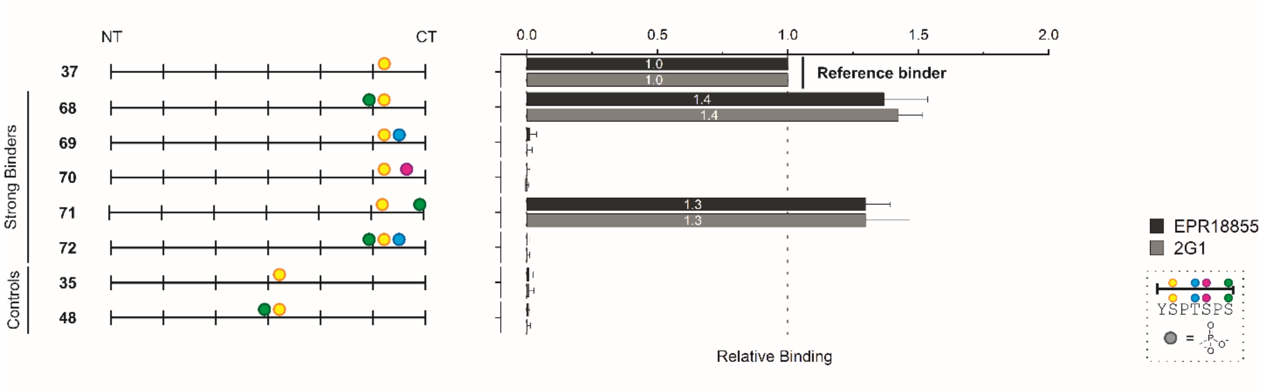

Figure S16 – Strong binders. ELISA with selected peptides bearing the phosphorylations on S2 of the C-terminal heptad was performed by applying conditions described on page 32. Strong binding peptide **37** was used as the reference probe.

5.5 Structural studies

5.5.1 Circular dichroism measurements

CD measurements of the peptide (**2**-**6**) were performed using a Jasco-1500 circular dichroism spectropolarimeter from *Jasco Corporation* (Tokyo, Japan) with a quartz cuvette of 0.2 cm path length at controlled temperature (25 °C). Temperature control was achieved by a Julabo F250 system. The analyte was dissolved at 20 µM in 100 mM NaF, 100 mM Tris-HCl, at pH 7.2. Secondary structure predictions were done with the website BeStSel.[5]

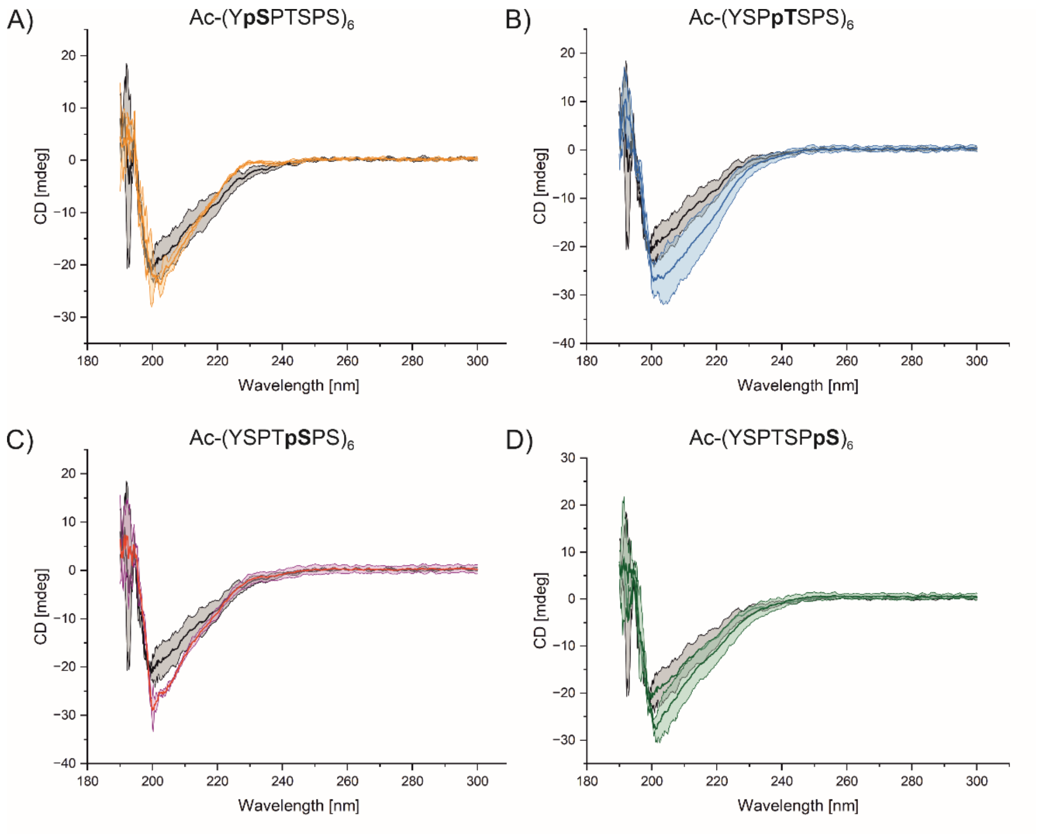

Figure S17 – CD spectra. The following spectra show the result of measurement for the phosphorylated peptides (A) **2**, (B) **3**, (C) **4** and (D) **5**, overlapped with the spectrum of the unphosphorylated peptide **6** (black line). The areas around the curves represent summation of the error bars based on triplicated measurements of each sample.

Peptides	**2**	**3**	**4**	**5**	**6**
Helix	4.3	19.9	15.0	12.3	6.1
β-strand	11.2	6.5	5.8	13.2	7.4
Turn	14.5	11.9	12.8	13.1	18.1
Others	69.9	61.7	66.4	61.4	68.4
NRMSD	0.03016	0.2717	0.03417	0.02762	0.02759

Table S5 – Secondary structure prediction by BeStSel. The values indicate the percentage of certain secondary structures adopted by phosphorylated (**2**-**5**) and unphosphorylated (**6**) peptides.

5.5.2 Synthesis of peptides samples for structural studies by NMR spectroscopy

Peptide probes for the NMR experiments (**73**-**76**) for native chemical ligation were assembled on RinkAmide resin as described in section 4.1.2 and purified by RP-HPLC.

YSPTSPS-YSPTSPS-NH_2, **73**

Yield: 2.63 µmol, 26%.
MW: 1455.5 g·mol^{-1} ($C_{64}H_{93}N_{15}O_{24}$).
UPLC-MS: t_R = 4.84 min (3 → 40 % B1 in 8 min); m/z = 728.8 ($C_{64}H_{95}N_{15}O_{24}$ $(M+2H)^{2+}$, calcd.: 728.8).

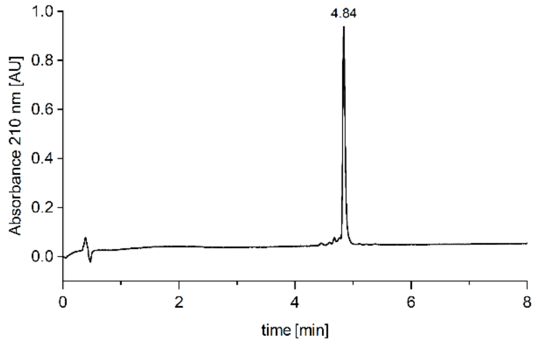

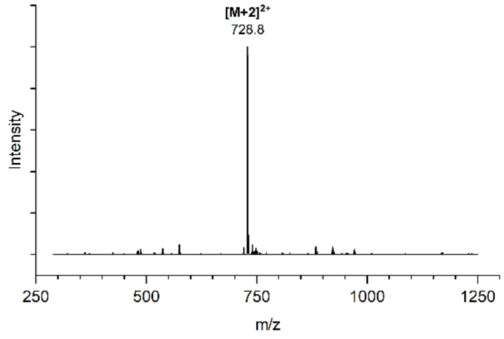

YSPTSPS-Y**pS**PTSPS-NH_2, **74**

Yield: 4.00 µmol, 40%.
MW: 1536.5 g·mol^{-1} ($C_{64}H_{94}N_{15}O_{27}P$).
UPLC-MS: t_R = 4.67 min (3 → 40 % B1 in 8 min); m/z = 769.0 ($C_{64}H_{96}N_{15}O_{27}P$ $(M+2H)^{2+}$, calcd.: 769.3).

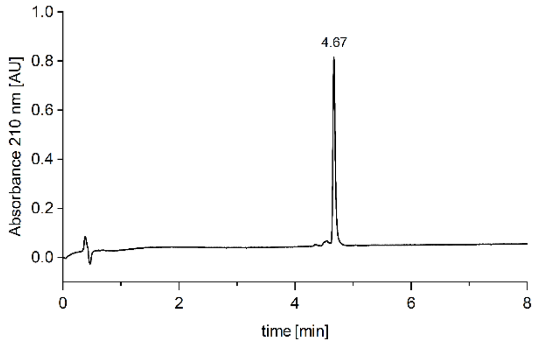

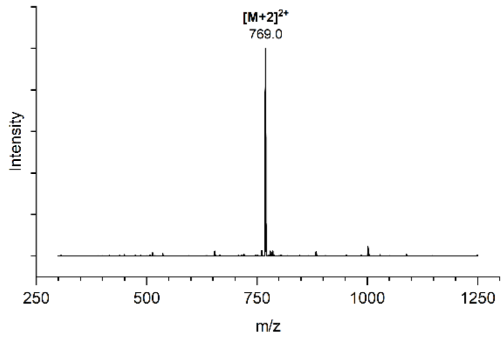

YSPTSP**pS**-Y**pS**PTSPS-NH_2, **75**

Yield: 2.73 µmol, 27%.
MW: 1616.5 g·mol^{-1} ($C_{65}H_{95}N_{15}O_{30}P_2$).
UPLC-MS: t_R = 4.67 min (3 → 40 % B1 in 8 min); m/z = 809.0 ($C_{65}H_{97}N_{15}O_{30}P_2$ $(M+2H)^{2+}$, calcd.: 809.3).

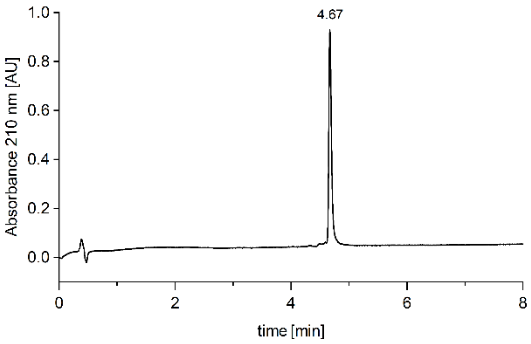

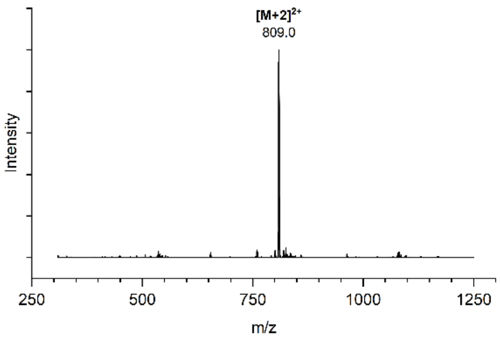

YSPTSPS-Y**pS**P**pT**SPS-NH_2, **76**

Yield: 2.69 µmol, 27%.
MW: 1616.5 g·mol^{-1} ($C_{65}H_{95}N_{15}O_{30}P_2$).
UPLC-MS: t_R = 4.66 min (3 → 40 % B1 in 8 min); m/z = 809.0 ($C_{65}H_{97}N_{15}O_{30}P_2$ $(M+2H)^{2+}$, calcd.: 809.3).

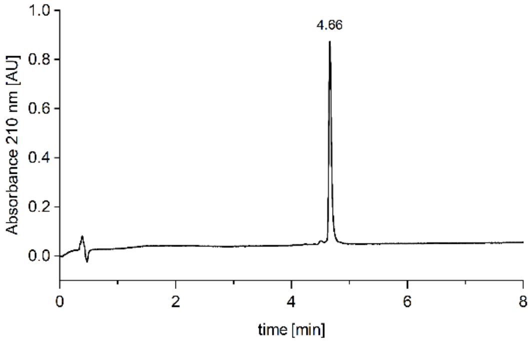

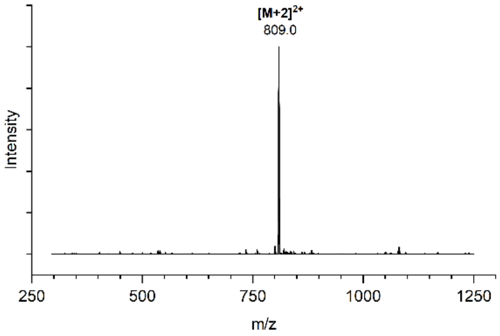

Figure S18 – Analysis and characterization of the probes for NMR. The percentage yields of the pure C-terminal fragments are calculated on the theoretical full scale of the synthesis.

5.5.3 NMR measurements

The NMR measurements were performed on a Avance NEO 500 MHz equipped with a H/C/N TCI CryoProbe Prodigy from *Bruker Biospin GmbH* (Rheinstetten, Germany). For processing and analyzing the spectra TopSpin 4.09 also from *Bruker Biospin GmbH* was used. The di-peptides were dissolved in water with 5 % D_2O, 4mM NaN_3, 0.1 mM DSS, pH 5.5. The concentration of the peptides varied from 0.5 mM to 1 mM. The value of pH was adjusted with NaOH. 3-(Trimethylsilyl)-1-propanesulfonic acid-d6 sodium salt was used as internal reference with chemical shift at 0.00 ppm. All measurements were carried out with a sample volume of 600µL in a 5 mm NMR tube (Boroeco-5-7) at 298K. Both one-dimensional ^1H spectra (pulse program: zgesgp) as well as two-dimensional ^1H-^1H-TOCSY spectra (pulse program: dipsi2esgpph), ^1H-^1H-ROESY spectra (pulse program: roesyesgpph), ^1H-^{15}N-HSQC spectra (pulse program: hsqcetf3gp) and ^1H-^{13}C-HSQC spectra (pulse program: hsqcedetgp) were recorded. The number of recorded data points was 2048 in the ^1H dimension, 512 in the second ^1H dimension and 256 in the ^{13}C dimension and 64 in ^{15}N dimension. DQD (digital quadrature detection) was used as the recording mode and a forward linear prediction of complex data points was carried out for the Fourier transformation in the indirect dimension.

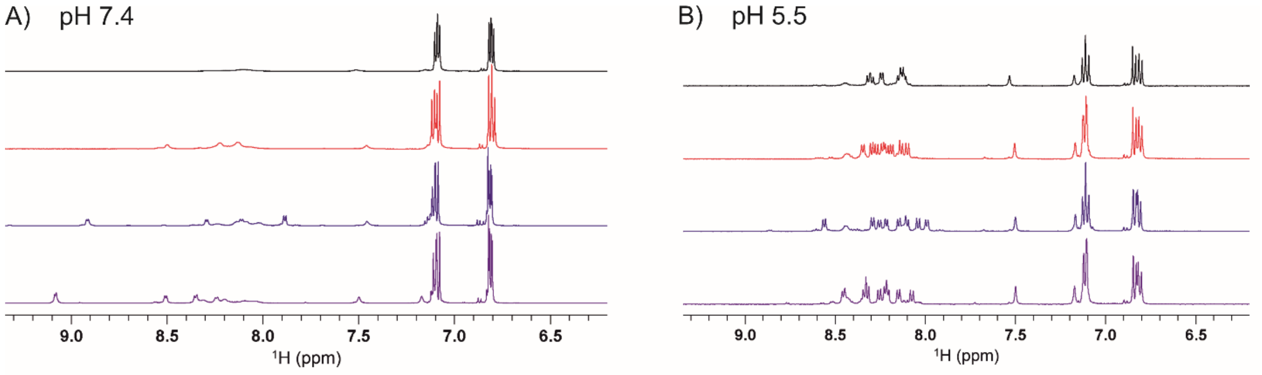

Figure S19 – ^1H NMR spectra of the amide proton region of peptides **73** (black), **74** (red), **75** (blue) and **76** (violet) at pH 7.4 (left) and 5.5 (right).

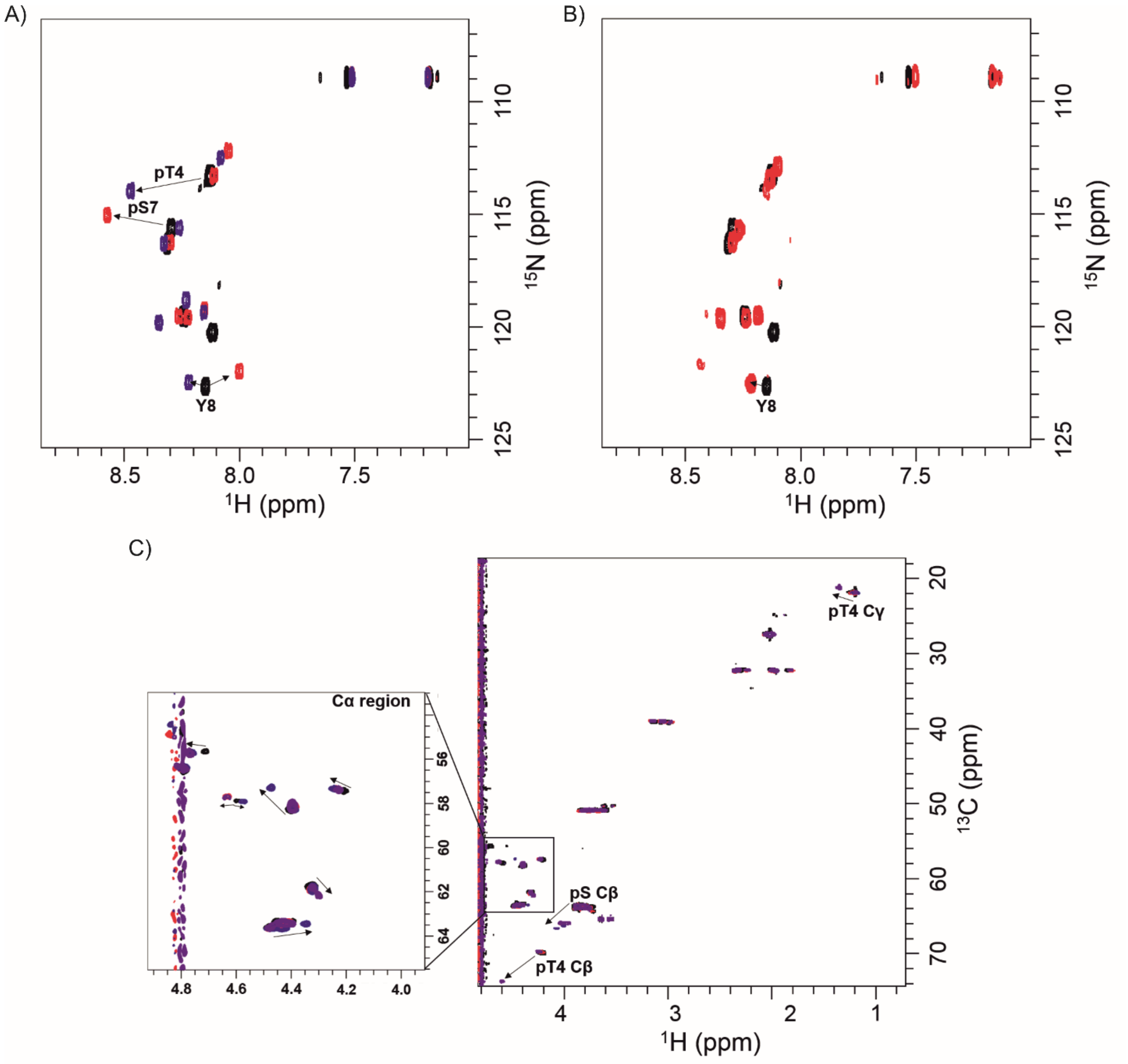

Figure S20 – A) Superposition of ^{1}H-^{15}N-HSQC spectra of non-phosphorylated CTD (**73**, black), phosphorylated pSer7 and pSer2 (**75**, red), phosphorylated pSer2 and pThr4 (**76**, blue), B) superposition of ^{1}H-^{15}N-HSQC spectra of non-phosphorylated CTD (**73**, black) and phosphorylated pSer2 (**74**, red), C) superposition of ^{1}H-^{13}C-HSQC spectra of overlapping of peptides **73** (black), **74** (red), **75** (blue) and **76** (violet).

6. Characterization of peptides tested in ELISA for antibody binding

In UPLC, the detector was set to 210 nm, to enable detection of impurities that do not contain Trp. Mass spectrometry was done by injecting samples with buffers containing formic acid, in order to enhance the ionization and detection of the analytes. Remaining impurities mostly are comprised of benzylated and *tert*-butylated products deriving from TFA cleavage. The yield was determined by OD measurement on the NanoDrop machine at 274 nm.

HHHHHH-PEG$_6$-Y**pS**PTSPS-Y**pS**PTSPS-Y**pS**PTSPS-Y**pS**PTSPS-Y**pS**PTSPS-Y**pS**PTSPS-NH$_2$, **7**

Yield: 0.79 µmol, 16%.
MW: 5973.6 g·mol^{-1} ($C_{243}H_{350}N_{62}O_{103}P_6$).
UPLC-MS: t_R = 4.36 min (3 → 40 % B1 in 8 min); m/z = 1195.6 ($C_{243}H_{354}N_{62}O_{103}P_6$ (M+5H)$^{5+}$, calcd.: 1195.7).

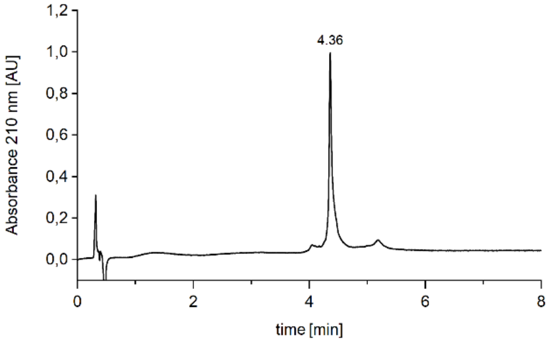

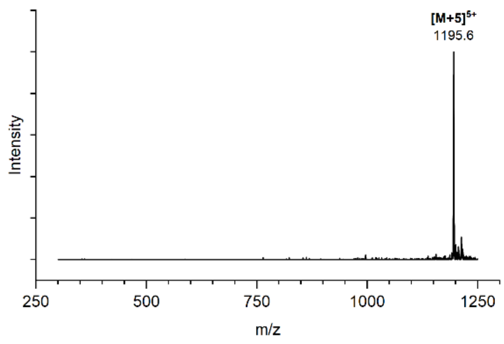

HHHHHH-PEG$_6$-YSPTSPS-YSPTSPS-YSPTSPS-YSPTSPS-YSPTSPS-YSPTSPS-NH$_2$, **31**

Yield: 2.17 µmol, 43%.
MW: 5493.8 g·mol^{-1} ($C_{243}H_{344}N_{62}O_{85}$).
UPLC-MS: t_R = 4.53 min (3 → 40 % B1 in 8 min); m/z = 1099.6 ($C_{243}H_{349}N_{62}O_{85}$ (M+5H)$^{5+}$, calcd.: 1099.8), 916.5 ($C_{243}H_{350}N_{62}O_{85}$ (M+6H)$^{6+}$, calcd.: 916.6), 785.8 ($C_{243}H_{351}N_{62}O_{85}$ (M+7H)$^{7+}$, calcd.: 785.8).

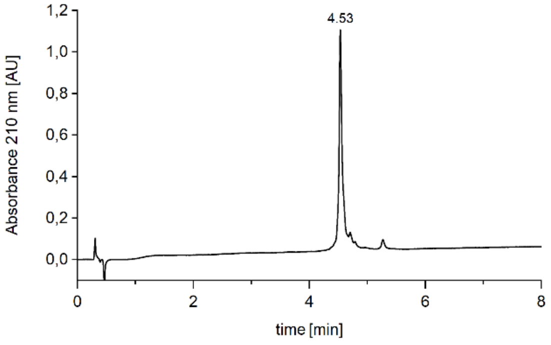

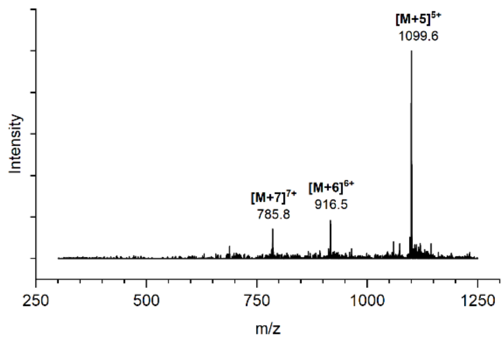

HHHHHH-PEG$_6$-Y**pS**PTSPS-YSPTSPS-YSPTSPS-YSPTSPS-YSPTSPS-YSPTSPS-NH$_2$, **32**

Yield: 0.51 µmol, 10%.
MW: 5573.8 g·mol^{-1} ($C_{243}H_{345}N_{62}O_{88}P$).
UPLC-MS: t_R = 4.54 min (3 → 40 % B1 in 8 min); m/z = 1115.6 ($C_{243}H_{350}N_{62}O_{88}P$ (M+5H)$^{5+}$, calcd.: 1115.8).

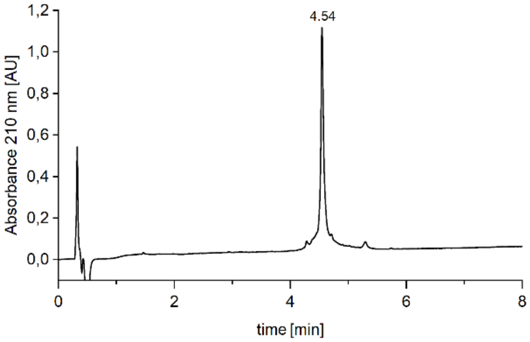

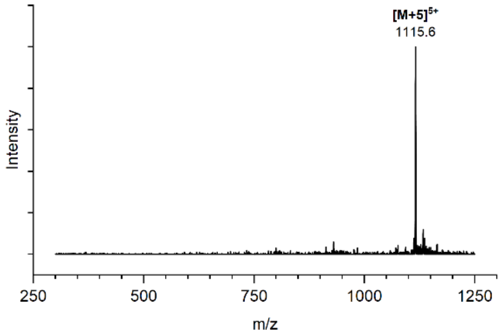

HHHHHH-PEG$_6$-YSPTSPS-Y**pS**PTSPS-YSPTSPS-YSPTSPS-YSPTSPS-YSPTSPS-NH$_2$, **33**

Yield: 1.98 µmol, 40%.
MW: 5573.8 g·mol^{-1} ($C_{243}H_{345}N_{62}O_{88}P$).
UPLC-MS: t_R = 4.52 min (3 → 40 % B1 in 8 min); m/z = 1115.8 ($C_{243}H_{350}N_{62}O_{88}P$ (M+5H)$^{5+}$.

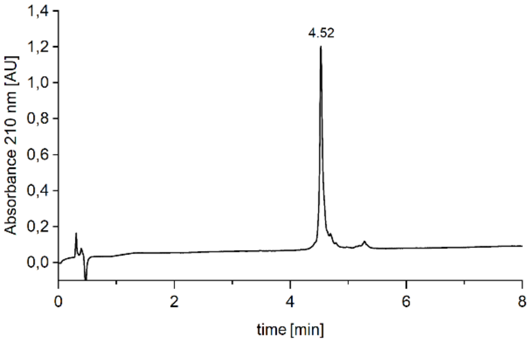

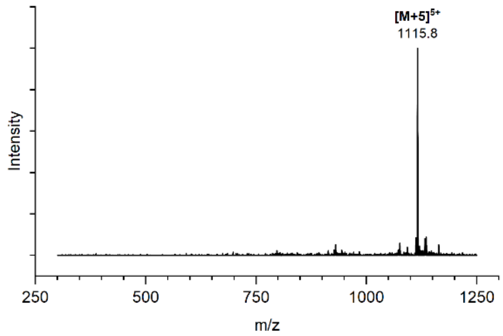

HHHHHH-PEG$_6$-YSPTSPS-YSPTSPS-Y**pS**PTSPS-YSPTSPS-YSPTSPS-YSPTSPS-NH$_2$, **34**

Yield: 1.93 µmol, 39%.
MW: 5573.8 g·mol^{-1} ($C_{243}H_{345}N_{62}O_{88}P$).
UPLC-MS: t_R = 4.50 min (3 → 40 % B1 in 8 min); m/z = 1115.7 ($C_{243}H_{350}N_{62}O_{88}P$ $(M+5H)^{5+}$, calcd.: 1115.8), 930.0 ($C_{243}H_{351}N_{62}O_{88}P$ $(M+6H)^{6+}$, calcd.: 930.0).

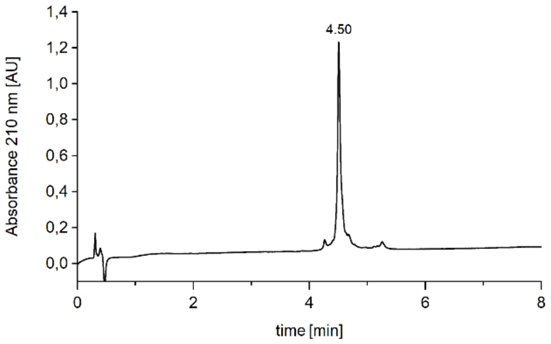

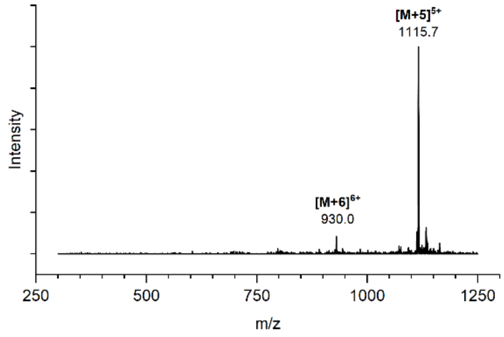

HHHHHH-PEG$_6$-YSPTSPS-YSPTSPS-YSPTSPS-Y**pS**PTSPS-YSPTSPS-YSPTSPS-NH$_2$, **35**

Yield: 2.04 µmol, 41%.
MW: 5573.8 g·mol^{-1} ($C_{243}H_{345}N_{62}O_{88}P$).
UPLC-MS: t_R = 4.50 min (3 → 40 % B1 in 8 min); m/z = 1115.8 ($C_{243}H_{350}N_{62}O_{88}P$ $(M+5H)^{5+}$, calcd.: 1115.8).

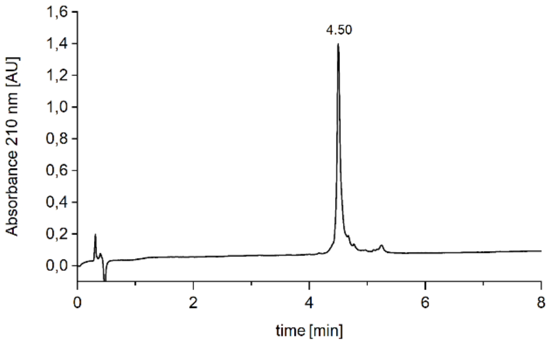

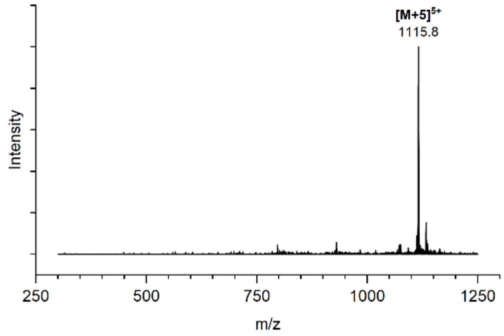

HHHHHH-PEG$_6$-YSPTSPS-YSPTSPS-YSPTSPS-YSPTSPS-Y**pS**PTSPS-YSPTSPS-NH$_2$, **36**

Yield: 1.62 µmol, 32%.
MW: 5573.8 g·mol^{-1} ($C_{243}H_{345}N_{62}O_{88}P$).
UPLC-MS: t_R = 4.49 min (3 → 40 % B1 in 8 min); m/z = 1115.8 ($C_{243}H_{350}N_{62}O_{88}P$ $(M+5H)^{5+}$, calcd.: 1115.8).

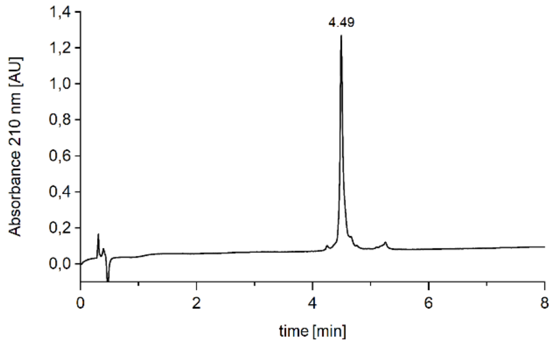

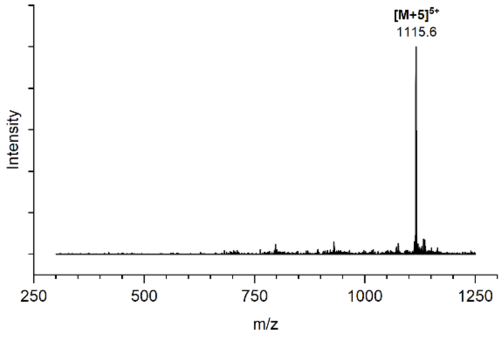

HHHHHH-PEG$_6$-YSPTSPS-YSPTSPS-YSPTSPS-YSPTSPS-YSPTSPS-Y**pS**PTSPS-NH$_2$, **37**

Yield: 1.57 µmol, 31%.
MW: 5573.8 g·mol^{-1} ($C_{243}H_{345}N_{62}O_{88}P$).
UPLC-MS: t_R = 4.51 min (3 → 40 % B1 in 8 min); m/z = 1115.6 ($C_{243}H_{350}N_{62}O_{88}P$ $(M+5H)^{5+}$, calcd.: 1115.8).

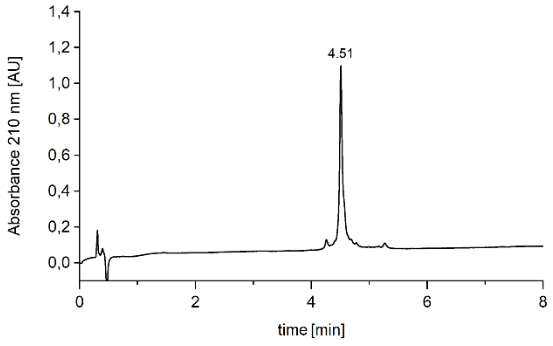

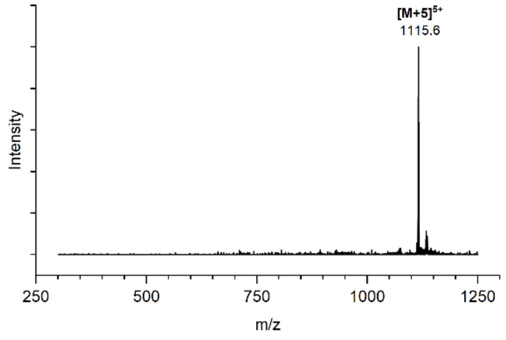

HHHHHH-PEG$_6$-YSPTSPS-YSPTSPS-YSPTSPS-YSPTSPS-Y**pS**PTSPS-Y**pS**PTSPS-NH$_2$, **38**

Yield: 2.37 µmol, 47%.
MW: 5653.7 g·mol^{-1} ($C_{243}H_{346}N_{62}O_{91}P_2$).
UPLC-MS: t_R = 4.53 min (3 → 40 % B1 in 8 min); m/z = 1131.4 ($C_{243}H_{351}N_{62}O_{91}P_2$ (M+5H)$^{5+}$, calcd.: 1131.8).

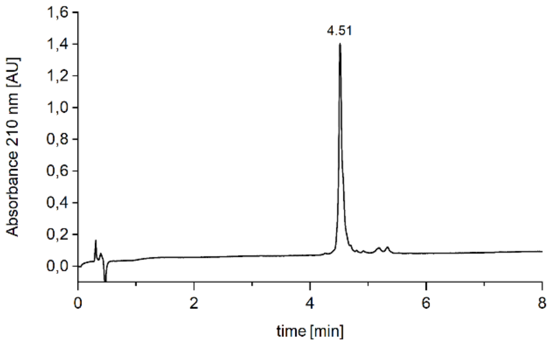

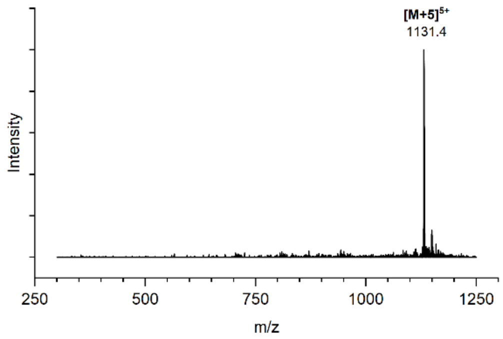

HHHHHH-PEG$_6$-YSPTSPS-YSPTSPS-YSPTSPS-Y**pS**PTSPS-YSPTSPS-Y**pS**PTSPS-NH$_2$, **39**

Yield: 1.94 µmol, 39%.
MW: 5653.7 g·mol^{-1} ($C_{243}H_{346}N_{62}O_{91}P_2$).
UPLC-MS: t_R = 4.48 min (3 → 40 % B1 in 8 min); m/z = 1131.5 ($C_{243}H_{351}N_{62}O_{91}P_2$ (M+5H)$^{5+}$, calcd.: 1131.8).

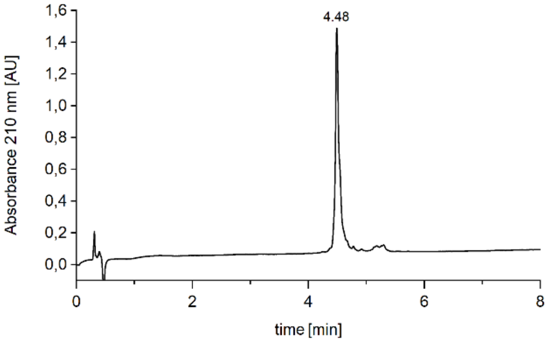

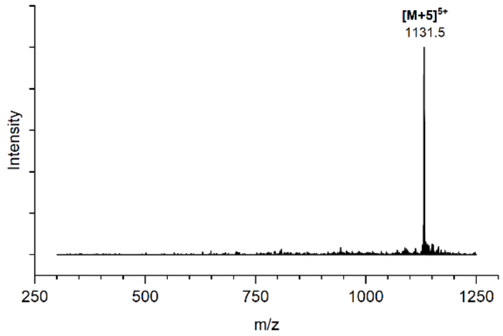

HHHHHH-PEG$_6$-YSPTSPS-YSPTSPS-Y**pS**PTSPS-YSPTSPS-YSPTSPS-Y**pS**PTSPS-NH$_2$, **40**

Yield: 1.75 µmol, 35%.
MW: 5653.7 g·mol^{-1} ($C_{243}H_{346}N_{62}O_{91}P_2$).
UPLC-MS: t_R = 4.49 min (3 → 40 % B1 in 8 min); m/z = 1131.8 ($C_{243}H_{351}N_{62}O_{91}P_2$ $(M+5H)^{5+}$, calcd.: 1131.8).

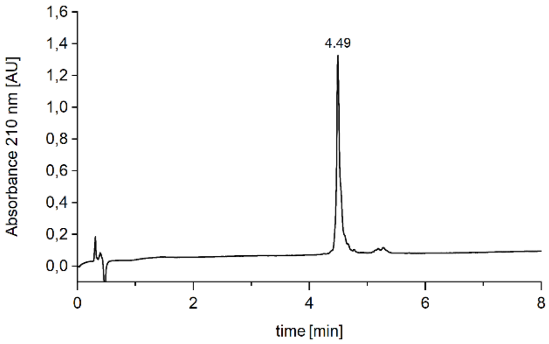

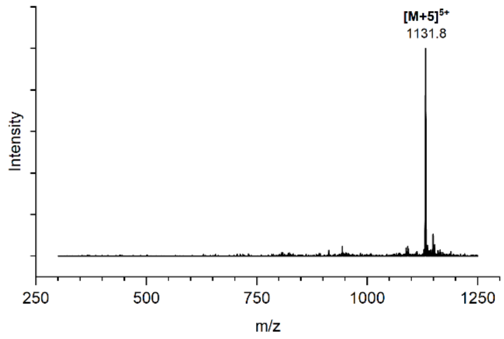

HHHHHH-PEG$_6$-YSPTSPS-Y**pS**PTSPS-YSPTSPS-YSPTSPS-YSPTSPS-Y**pS**PTSPS-NH$_2$, **41**

Yield: 1.86 µmol, 37%.
MW: 5653.7 g·mol^{-1} ($C_{243}H_{346}N_{62}O_{91}P_2$).
UPLC-MS: t_R = 4.49 min (3 → 40 % B1 in 8 min); m/z = 1131.6 ($C_{243}H_{351}N_{62}O_{91}P_2$ $(M+5H)^{5+}$, calcd.: 1131.8).

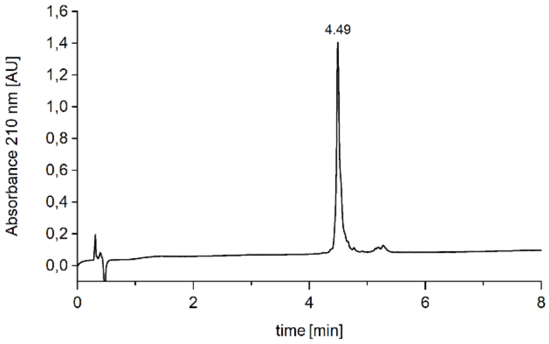

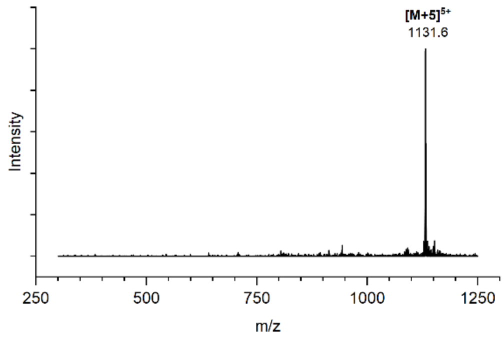

HHHHHH-PEG$_6$-Y**pS**PTSPS-YSPTSPS-YSPTSPS-YSPTSPS-YSPTSPS-Y**pS**PTSPS-NH$_2$, **42**

Yield: 1.63 µmol, 33%.
MW: 5653.7 g·mol^{-1} ($C_{243}H_{346}N_{62}O_{91}P_2$).
UPLC-MS: t_R = 4.51 min (3 → 40 % B1 in 8 min); m/z = 1131.8 ($C_{243}H_{351}N_{62}O_{91}P_2$ (M+5H)$^{5+}$, calcd.: 1131.8)

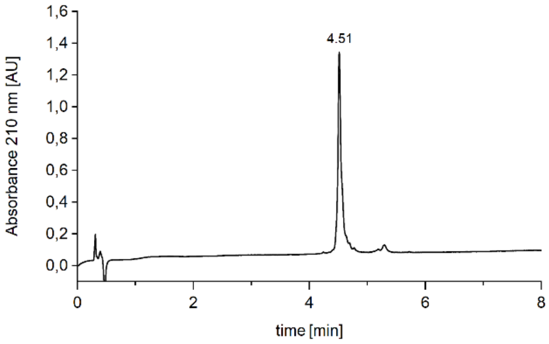

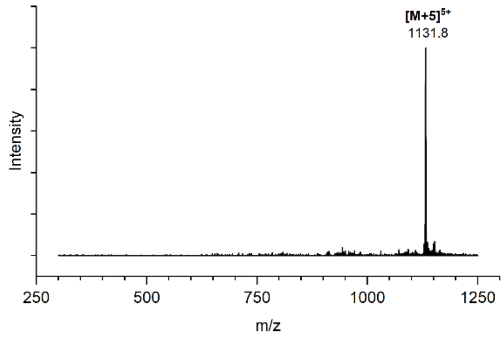

HHHHHH-PEG$_6$-YSPTSPS-YSPTSPS-Y**pS**PTSPS-Y**pS**PTSPS-YSPTSPS-YSPTSPS-NH$_2$, **43**

Yield: 0.88 µmol, 18%.
MW: 5653.7 g·mol^{-1} ($C_{243}H_{346}N_{62}O_{91}P_2$).
UPLC-MS: t_R = 4.43 min (3 → 40 % B1 in 8 min); m/z = 1131.4 ($C_{243}H_{351}N_{62}O_{91}P_2$ (M+5H)$^{5+}$, calcd.: 1131.8).

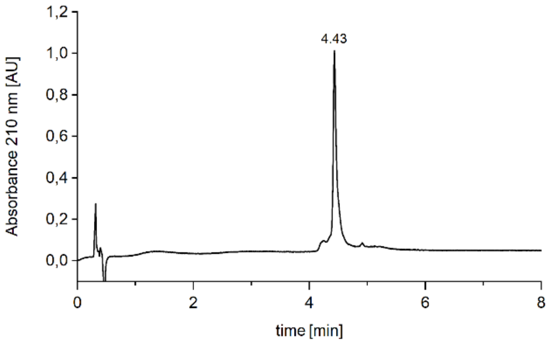

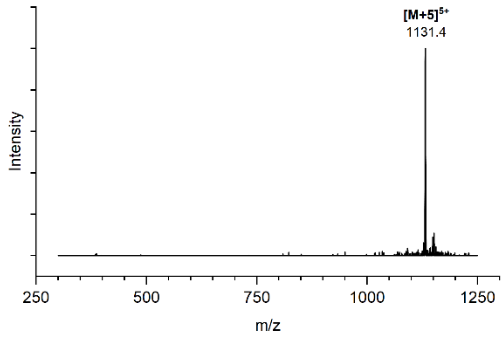

HHHHHH-PEG$_6$-YSPTSPS-YSPTSPS-YSPTSPS-Y**pS**PTSPS-Y**pS**PTSPS-YSPTSPS-NH$_2$, **44**

Yield: 1.83 µmol, 37%.
MW: 5653.7 g·mol^{-1} ($C_{243}H_{346}N_{62}O_{91}P_2$).
UPLC-MS: t_R = 4.40 min (3 → 40 % B1 in 8 min); m/z = 1131.9 ($C_{243}H_{351}N_{62}O_{91}P_2$ (M+5H)$^{5+}$, calcd.: 1131.8).

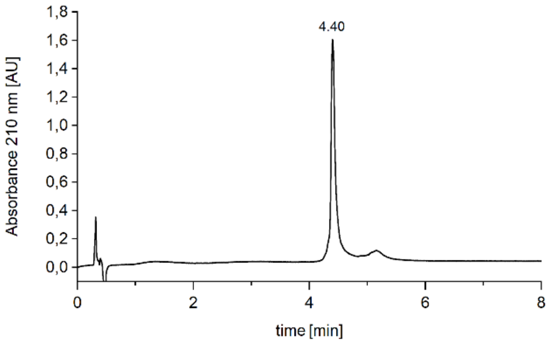

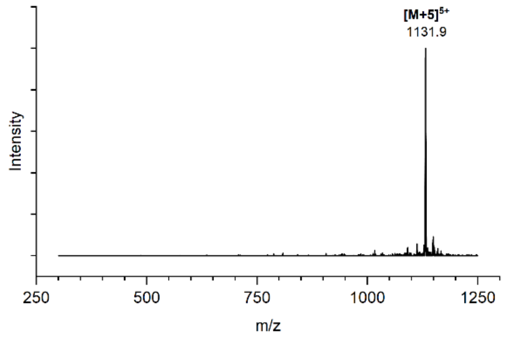

HHHHHH-PEG$_6$-YSPTSPS-YSPTSP**pS**-YSPTSPS-Y**pS**PTSPS-YSPTSPS-YSPTSPS-NH$_2$, **45**

Yield: 0.99 µmol, 20%.
MW: 5653.7 g·mol^{-1} ($C_{243}H_{346}N_{62}O_{91}P_2$).
UPLC-MS: t_R = 4.23 min (3 → 40 % B1 in 8 min); m/z = 1131.4 ($C_{243}H_{351}N_{62}O_{91}P_2$ (M+5H)$^{5+}$, calcd.: 1131.8).

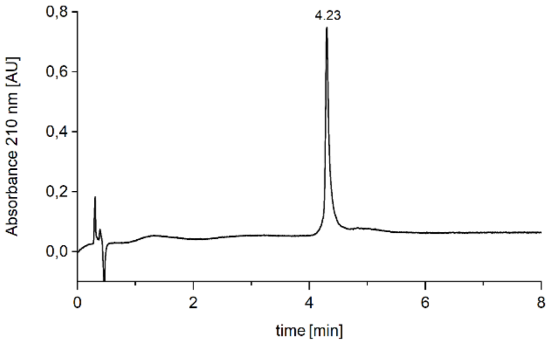

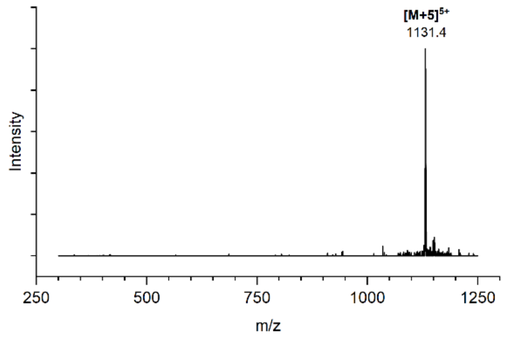

HHHHHH-PEG$_6$-YSPTSPS-YSPTSPS-YSP**pT**SPS-Y**pS**PTSPS-YSPTSPS-YSPTSPS-NH$_2$, **46**

Yield: 1.49 µmol, 30%.
MW: 5653.7 g·mol^{-1} ($C_{243}H_{346}N_{62}O_{91}P_2$).
UPLC-MS: t_R = 4.40 min (3 → 40 % B1 in 8 min); m/z = 1131.5 ($C_{243}H_{351}N_{62}O_{91}P_2$ (M+5H)$^{5+}$, calcd.: 1131.8).

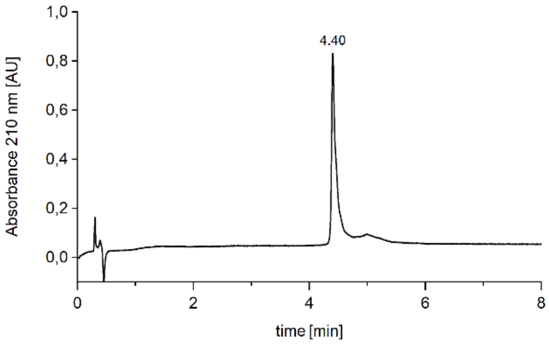

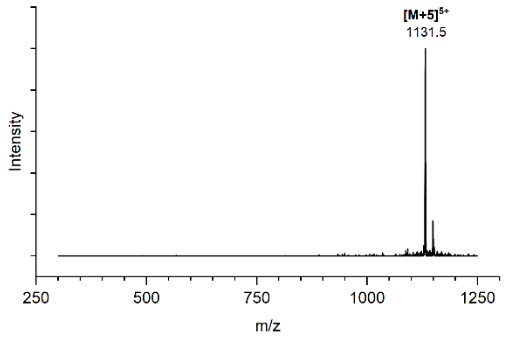

HHHHHH-PEG$_6$-YSPTSPS-YSPTSPS-YSPT**pS**PS-Y**pS**PTSPS-YSPTSPS-YSPTSPS-NH$_2$, **47**

Yield: 1.71 µmol, 34%.
MW: 5653.7 g·mol^{-1} ($C_{243}H_{346}N_{62}O_{91}P_2$).
UPLC-MS: t_R = 4.45 min (3 → 40 % B1 in 8 min); m/z = 1131.4 ($C_{243}H_{351}N_{62}O_{91}P_2$ (M+5H)$^{5+}$, calcd.: 1131.8).

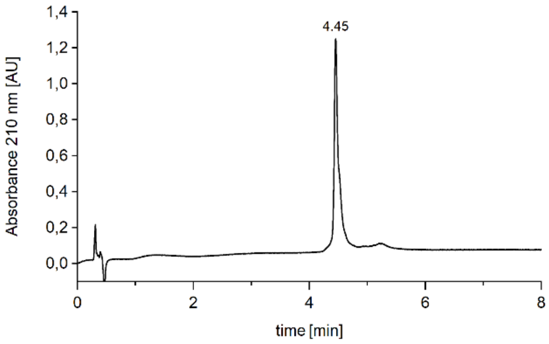

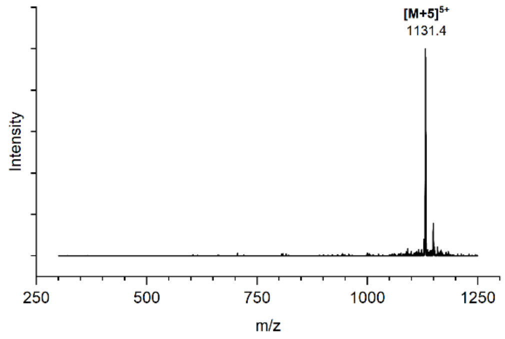

HHHHHH-PEG$_6$-YSPTSPS-YSPTSPS-YSPTSP**pS**-Y**pS**PTSPS-YSPTSPS-YSPTSPS-NH_2, **48**

Yield: 1.68 µmol, 34%.
MW: 5653.7 g·mol^{-1} ($C_{243}H_{346}N_{62}O_{91}P_2$).
UPLC-MS: t_R = 4.38 min (3 → 40 % B1 in 8 min); m/z = 1131.6 ($C_{243}H_{351}N_{62}O_{91}P_2$ $(M+5H)^{5+}$, calcd.: 1131.8).

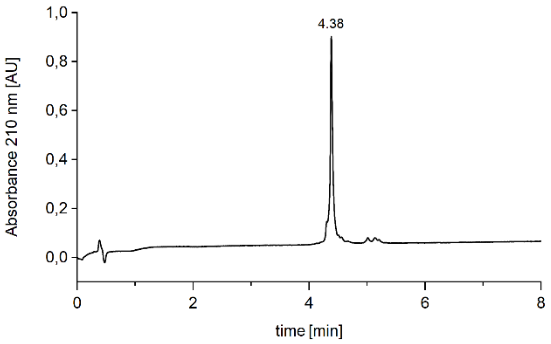

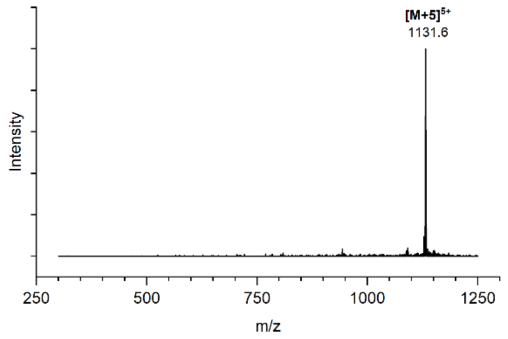

HHHHHH-PEG$_6$-YSPTSPS-YSPTSPS-YSPTSPS-Y**pS**P**pT**SPS-YSPTSPS-YSPTSPS-NH_2, **49**

Yield: 1.73 µmol, 35%.
MW: 5653.7 g·mol^{-1} ($C_{243}H_{346}N_{62}O_{91}P_2$).
UPLC-MS: t_R = 4.37 min (3 → 40 % B1 in 8 min); m/z = 1131.8 ($C_{243}H_{351}N_{62}O_{91}P_2$ $(M+5H)^{5+}$, calcd.: 1131.8).

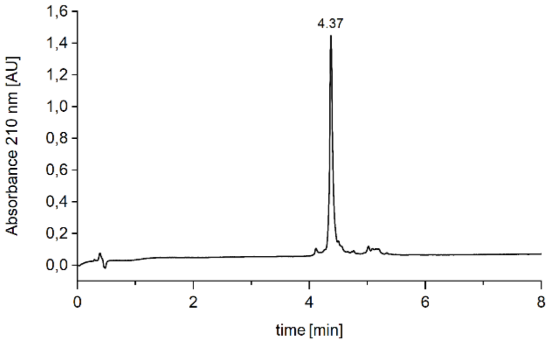

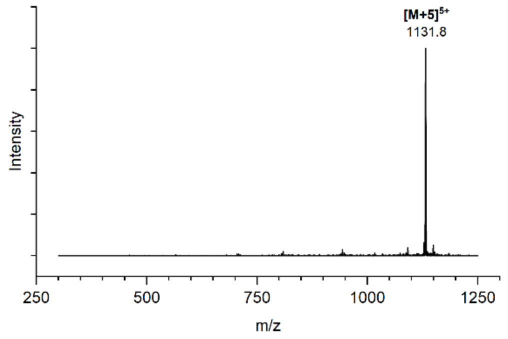

HHHHHH-PEG$_6$-YSPTSPS-YSPTSPS-YSPTSPS-Y**pS**PT**pS**PS-YSPTSPS-YSPTSPS-NH$_2$, **50**

Yield: 1.62 µmol, 32%.
MW: 5653.7 g·mol^{-1} ($C_{243}H_{346}N_{62}O_{91}P_2$).
UPLC-MS: t_R = 4.38 min (3 → 40 % B1 in 8 min); m/z = 1131.5 ($C_{243}H_{351}N_{62}O_{91}P_2$ $(M+5H)^{5+}$, calcd.: 1131.8).

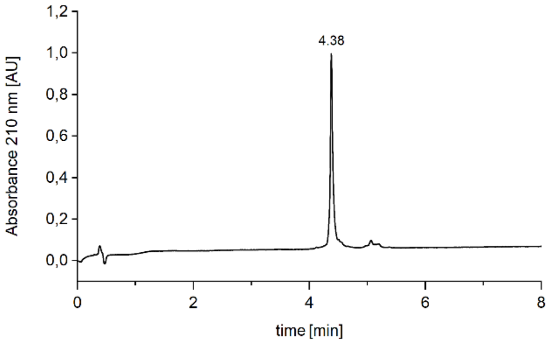

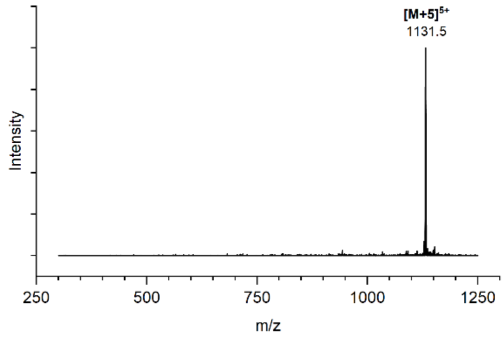

HHHHHH-PEG$_6$-YSPTSPS-YSPTSPS-YSPTSPS-Y**pS**PTSP**pS**-YSPTSPS-YSPTSPS-NH$_2$, **51**

Yield: 1.59 µmol, 32%.
MW: 5653.7 g·mol^{-1} ($C_{243}H_{346}N_{62}O_{91}P_2$).
UPLC-MS: t_R = 4.36 min (3 → 40 % B1 in 8 min); m/z = 1131.7 ($C_{243}H_{351}N_{62}O_{91}P_2$ $(M+5H)^{5+}$, calcd.: 1131.8).

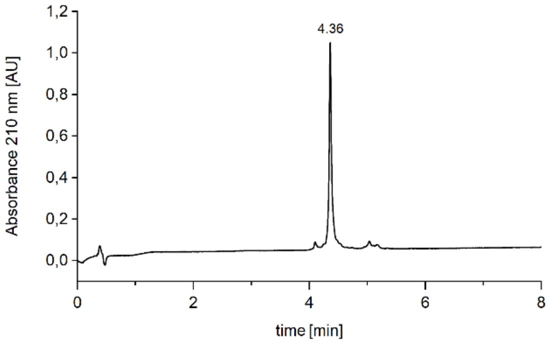

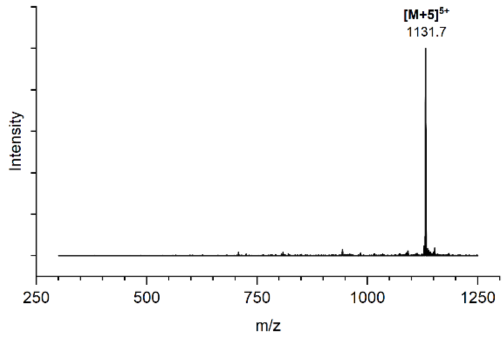

HHHHHH-PEG$_6$-YSPTSPS-YSPTSPS-YSPTSPS-Y**pS**PTSPS-YSP**pT**SPS-YSPTSPS-NH$_2$, **52**

Yield: 1.48 µmol, 30%.
MW: 5653.7 g·mol^{-1} ($C_{243}H_{346}N_{62}O_{91}P_2$).
UPLC-MS: t_R = 4.27 min (3 → 40 % B1 in 8 min); m/z = 1131.6 ($C_{243}H_{351}N_{62}O_{91}P_2$ (M+5H)$^{5+}$, calcd.: 1131.8).

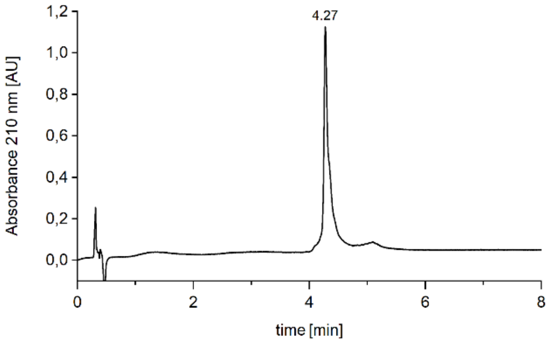

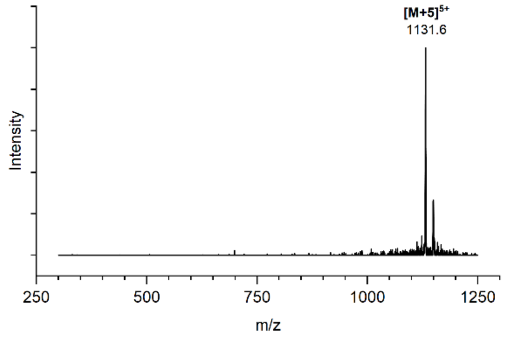

HHHHHH-PEG$_6$-YSPTSPS-YSPTSPS-YSPTSPS-Y**pS**PTSPS-YSPT**pS**PS-YSPTSPS-NH$_2$, **53**

Yield: 1.79 µmol, 36%.
MW: 5653.7 g·mol^{-1} ($C_{243}H_{346}N_{62}O_{91}P_2$).
UPLC-MS: t_R = 4.42 min (3 → 40 % B1 in 8 min); m/z = 1131.5 ($C_{243}H_{351}N_{62}O_{91}P_2$ (M+5H)$^{5+}$, calcd.: 1131.8).

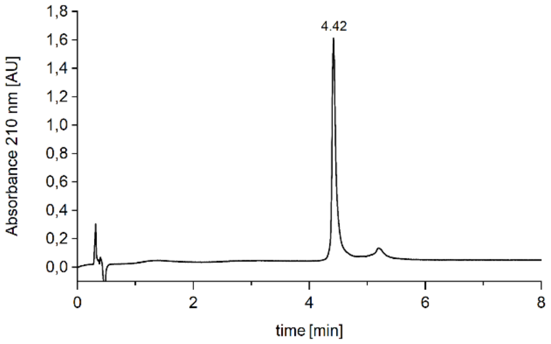

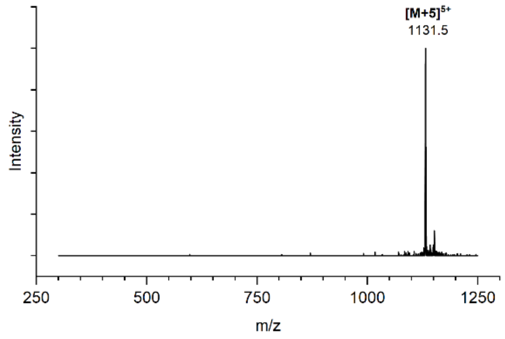

HHHHHH-PEG$_6$-YSPTSPS-YSPTSPS-YSPTSPS-Y**pS**PTSPS-YSPTSP**pS**-YSPTSPS-NH$_2$, **54**

Yield: 1.85 µmol, 37%.
MW: 5653.7 g·mol^{-1} ($C_{243}H_{346}N_{62}O_{91}P_2$).
UPLC-MS: t_R = 4.40 min (3 → 40 % B1 in 8 min); m/z = 1131.4 ($C_{243}H_{351}N_{62}O_{91}P_2$ $(M+5H)^{5+}$, calcd.: 1131.8).

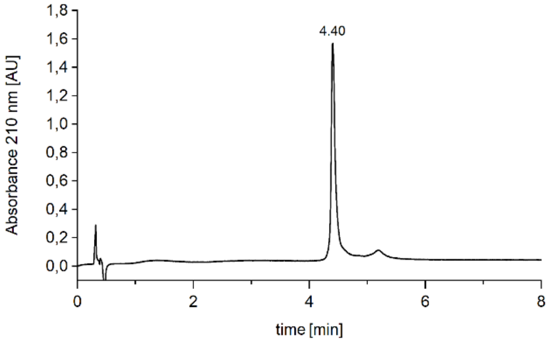

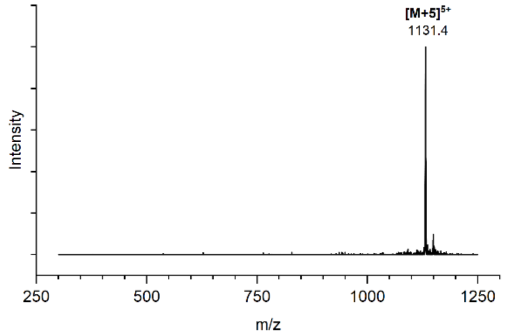

HHHHHH-PEG$_6$-YSPTSPS-YSPTSPS-YSPTSPE-Y**pS**PTSPS-YSPTSPS-YSPTSPS-NH$_2$, **55**

Yield: 2.33 µmol, 47%.
MW: 5615.8 g·mol^{-1} ($C_{245}H_{347}N_{62}O_{89}P$).
UPLC-MS: t_R = 4.40 min (3 → 40 % B1 in 8 min); m/z = 1123.9 ($C_{245}H_{352}N_{62}O_{89}P$ $(M+5H)^{5+}$, calcd.:1124.2).

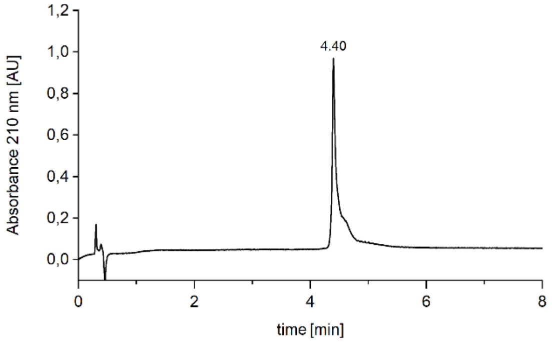

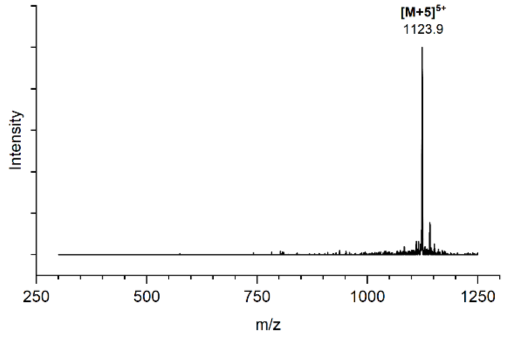

HHHHHH-PEG$_6$-YSP**pT**SPS-YSPTSPS-YSPTSPS-Y**pS**PTSPS-YSPTSPS-YSPTSPS-NH$_2$, **56**

Yield: 1.74 µmol, 35%.
MW: 5653.7 g·mol^{-1} ($C_{243}H_{346}N_{62}O_{91}P_2$).
UPLC-MS: t_R = 4.30 min (3 → 40 % B1 in 8 min); m/z = 1131.4 ($C_{243}H_{351}N_{62}O_{91}P_2$ (M+5H)$^{5+}$, calcd.: 1131.8).

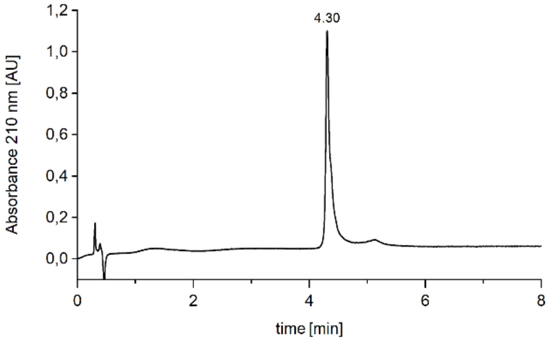

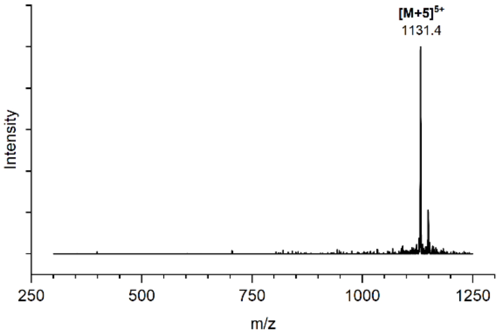

HHHHHH-PEG$_6$-YSPTSPS-YSP**pT**SPS-YSPTSPS-Y**pS**PTSPS-YSPTSPS-YSPTSPS-NH$_2$, **57**

Yield: 1.37 µmol, 27%.
MW: 5653.7 g·mol^{-1} ($C_{243}H_{346}N_{62}O_{91}P_2$).
UPLC-MS: t_R = 4.27 min (3 → 40 % B1 in 8 min); m/z = 1131.6 ($C_{243}H_{351}N_{62}O_{91}P_2$ (M+5H)$^{5+}$, calcd.: 1131.8).

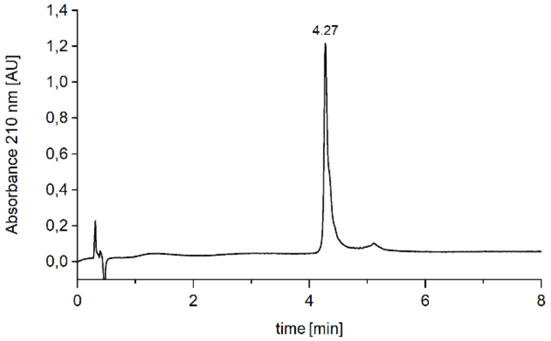

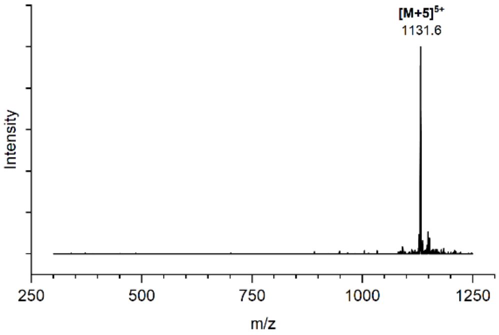

HHHHHH-PEG6-YSPTSPS-YSPTSPS-YSPTSPS-Y**pS**PTSPS-YSPTSPS-YSP**pT**SPS-NH2, **58**

Yield: 2.11 µmol, 42%.
MW: 5653.7 g·mol^{-1} ($C_{243}H_{346}N_{62}O_{91}P_2$).
UPLC-MS: t_R = 4.41 min (3 → 40 % B1 in 8 min); m/z = 1131.5 ($C_{243}H_{351}N_{62}O_{91}P_2$ $(M+5H)^{5+}$, calcd.: 1131.8).

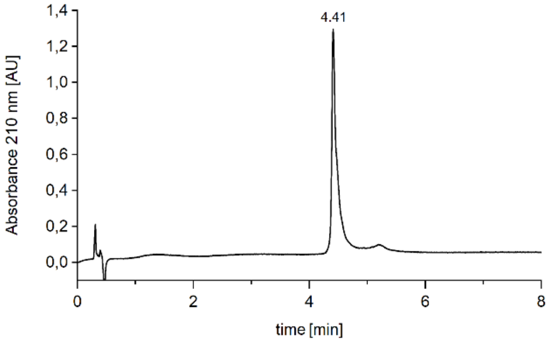

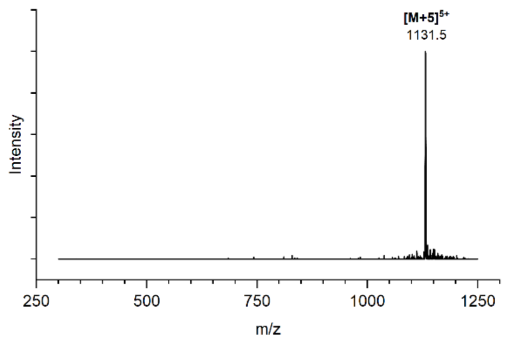

HHHHHH-PEG6-YSPTSPS-YSPTSP**pS**-YSPTSP**pS**-Y**pS**PTSPS-YSPTSPS-YSPTSPS-NH2, **59**

Yield: 1.74 µmol, 35%.
MW: 5733.7 g·mol^{-1} ($C_{243}H_{347}N_{62}O_{94}P_3$).
UPLC-MS: t_R = 4.46 min (3 → 40 % B1 in 8 min); m/z = 1147.8 ($C_{243}H_{352}N_{62}O_{94}P_3$ $(M+5H)^{5+}$, calcd.: 1147.7).

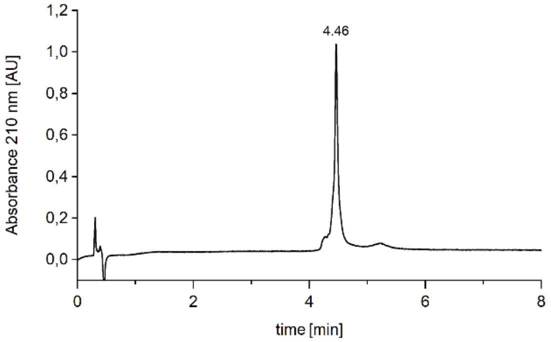

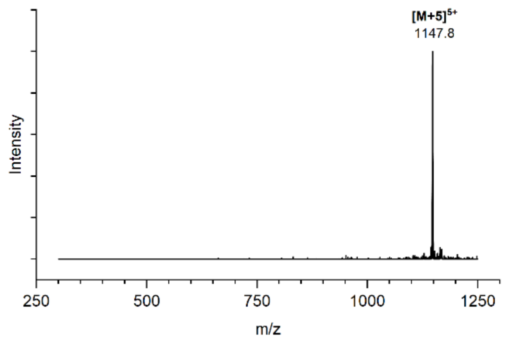

HHHHHH-PEG$_6$-YSPTSPS-YSPTSPS-Y**pS**PTSP**pS**-Y**pS**PTSPS-YSPTSPS-YSPTSPS-NH$_2$, **60**

Yield: 4.49 µmol, 29%.
MW: 5733.7 g·mol^{-1} ($C_{243}H_{347}N_{62}O_{94}P_3$).
UPLC-MS: t_R = 4.49 min (3 → 40 % B1 in 8 min); m/z = 1147.6 ($C_{243}H_{352}N_{62}O_{94}P_3$ $(M+5H)^{5+}$, calcd.: 1147.7).

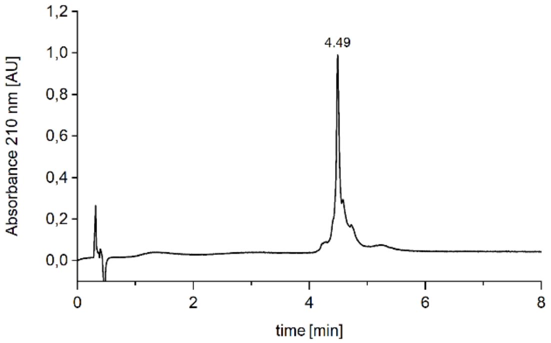

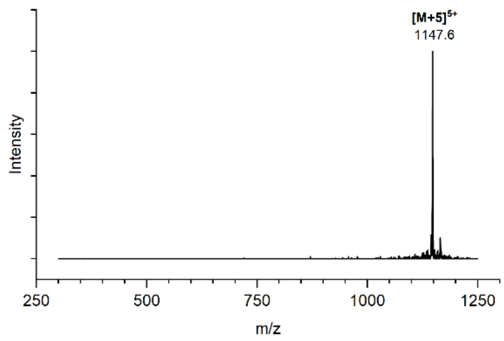

HHHHHH-PEG$_6$-YSPTSPS-YSPTSPS-YSP**pT**SP**pS**-Y**pS**PTSPS-YSPTSPS-YSPTSPS-NH$_2$, **61**

Yield: 0.99 µmol, 20%.
MW: 5733.7 g·mol^{-1} ($C_{243}H_{347}N_{62}O_{94}P_3$).
UPLC-MS: t_R = 4.49 min (3 → 40 % B1 in 8 min); m/z = 1147.6 ($C_{243}H_{352}N_{62}O_{94}P_3$ $(M+5H)^{5+}$, calcd.: 1147.7).

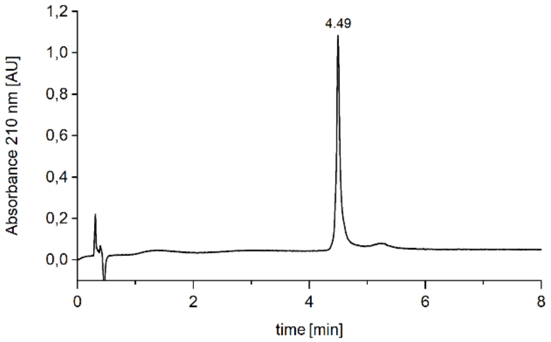

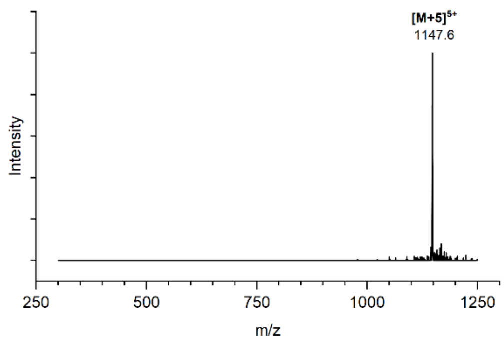

HHHHHH-PEG$_6$-YSPTSPS-YSPTSPS-YSPT**pS**P**pS**-Y**pS**PTSPS-YSPTSPS-YSPTSPS-NH$_2$, **62**

Yield: 0.38 µmol, 8%.
MW: 5733.7 g·mol^{-1} ($C_{243}H_{347}N_{62}O_{94}P_3$).
UPLC-MS: t_R = 4.50 min (3 → 40 % B1 in 8 min); m/z = 1147.9 ($C_{243}H_{352}N_{62}O_{94}P_3$ $(M+5H)^{5+}$, calcd.: 1147.7).

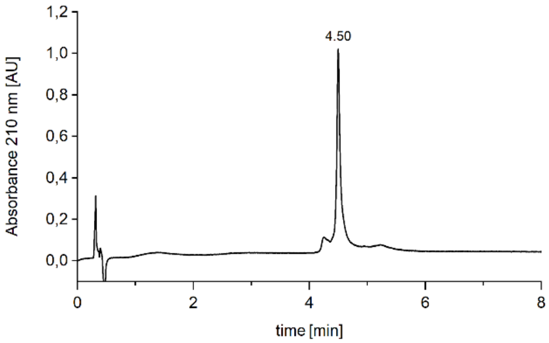

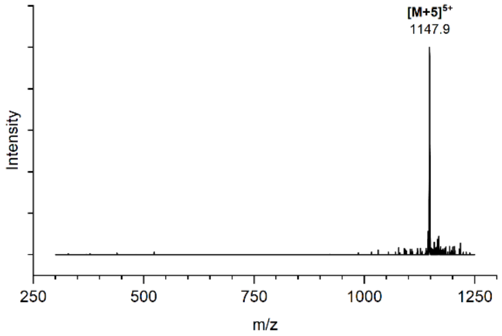

HHHHHH-PEG$_6$-YSPTSPS-YSPTSPS-YSPTSP**pS**-Y**pS**P**pT**SPS-YSPTSPS-YSPTSPS-NH$_2$, **63**

Yield: 1.71 µmol, 34%.
MW: 5733.7 g·mol^{-1} ($C_{243}H_{347}N_{62}O_{94}P_3$).
UPLC-MS: t_R = 4.40 min (3 → 40 % B1 in 8 min); m/z = 1148.0 ($C_{243}H_{352}N_{62}O_{94}P_3$ $(M+5H)^{5+}$, calcd.: 1147.7).

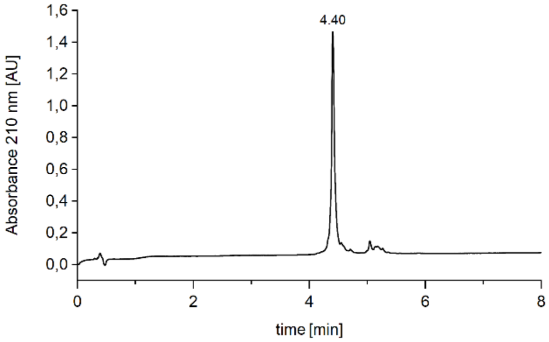

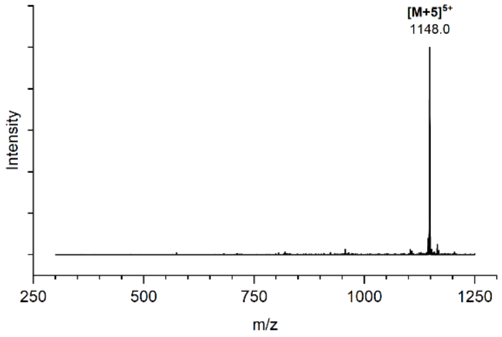

HHHHHH-PEG$_6$-YSPTSPS-YSPTSPS-YSPTSP**pS**-Y**pS**PT**pS**PS-YSPTSPS-YSPTSPS-NH$_2$, **64**

Yield: 0.71 µmol, 14%.
MW: 5733.7 g·mol^{-1} ($C_{243}H_{347}N_{62}O_{94}P_3$).
UPLC-MS: t_R = 4.52 min (3 → 40 % B1 in 8 min); m/z = 1147.4 ($C_{243}H_{352}N_{62}O_{94}P_3$ $(M+5H)^{5+}$, calcd.: 1147.7),

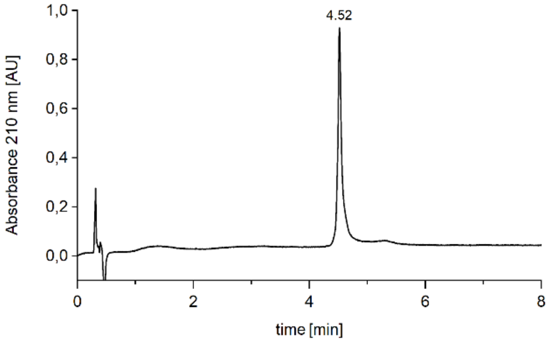

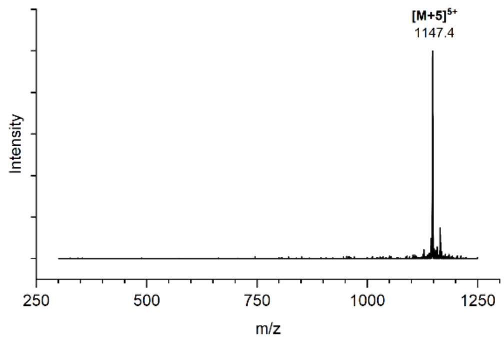

HHHHHH-PEG$_6$-YSPTSPS-YSPTSPS-YSPTSP**pS**-Y**pS**PTSP**pS**-YSPTSPS-YSPTSPS-NH$_2$, **65**

Yield: 1.73 µmol, 35%.
MW: 5733.7 g·mol^{-1} ($C_{243}H_{347}N_{62}O_{94}P_3$).
UPLC-MS: t_R = 4.47 min (3 → 40 % B1 in 8 min); m/z = 1147.2 ($C_{243}H_{352}N_{62}O_{94}P_3$ $(M+5H)^{5+}$, calcd.: 1147.7).

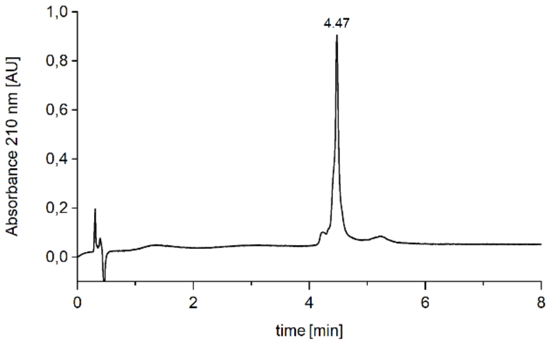

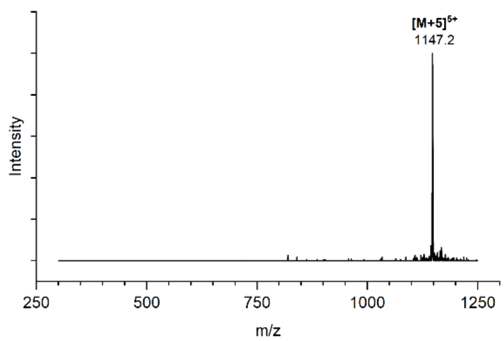

HHHHHH-PEG6-YSPTSPS-YSPTSPS-YSPTSP**pS**-Y**pS**PTSPS-Y**pS**PTSPS-YSPTSPS-NH2, **66**

Yield: 1.78 µmol, 36%.
MW: 5733.7 g·mol−1 ($C_{243}H_{347}N_{62}O_{94}P_3$).
UPLC-MS: t_R = 4.46 min (3 → 40 % B1 in 8 min); m/z = 1147.7 ($C_{243}H_{352}N_{62}O_{94}P_3$ $(M+5H)^{5+}$, calcd.: 1147.7).

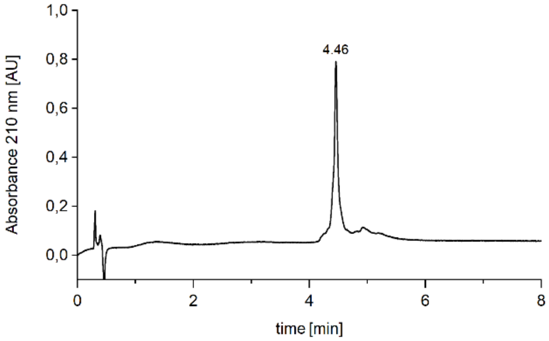

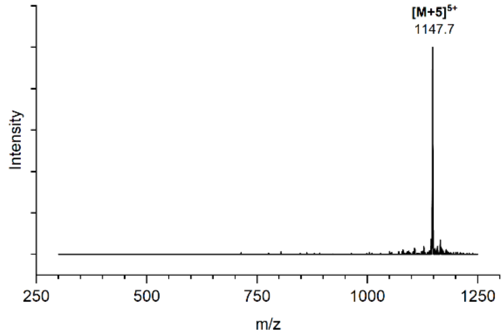

HHHHHH-PEG6-YSPTSPS-YSPTSPS-YSPTSP**pS**-Y**pS**PTSPS-YSP**pT**SPS-YSPTSPS-NH2, **67**

Yield: 1.69 µmol, 34%.
MW: 5733.7 g·mol−1 ($C_{243}H_{347}N_{62}O_{94}P_3$).
UPLC-MS: t_R = 4.46 min (3 → 40 % B1 in 8 min); m/z = 1147.4 ($C_{243}H_{352}N_{62}O_{94}P_3$ $(M+5H)^{5+}$, calcd.: 1147.7).

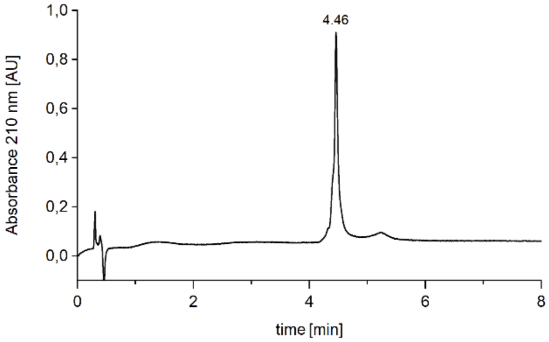

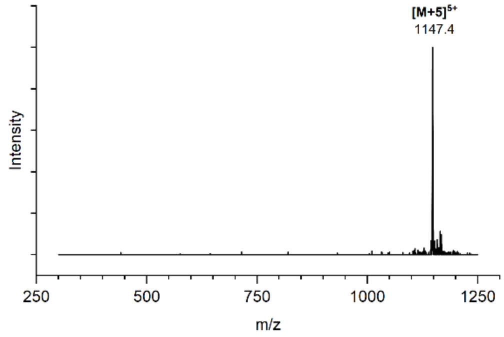

HHHHHH-PEG$_6$-YSPTSPS-YSPTSPS-YSPTSPS-YSPTSPS-YSPTSP**pS**-Y**pS**PTSPS-NH$_2$, **68**

Yield: 1.16 µmol, 23%.
MW: 5653.7 g·mol^{-1} ($C_{243}H_{346}N_{62}O_{91}P_2$).
UPLC-MS: t_R = 4.41 min (3 → 40 % B1 in 8 min); m/z = 1131.8 ($C_{243}H_{351}N_{62}O_{91}P_2$ $(M+5H)^{5+}$, calcd.: 1131.8).

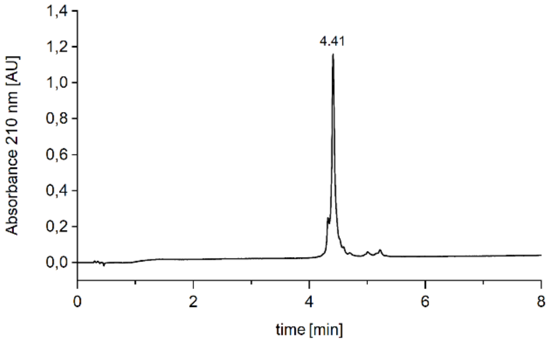

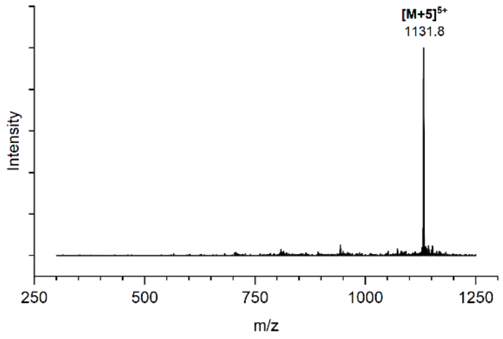

HHHHHH-PEG$_6$-YSPTSPS-YSPTSPS-YSPTSPS-YSPTSPS-YSPTSPS-Y**pS**P**pT**SPS-NH$_2$, **69**

Yield: 1.10 µmol, 22%.
MW: 5653.7 g·mol^{-1} ($C_{243}H_{346}N_{62}O_{91}P_2$).
UPLC-MS: t_R = 4.40 min (3 → 40 % B1 in 8 min); m/z = 1131.5 ($C_{243}H_{351}N_{62}O_{91}P_2$ $(M+5H)^{5+}$, calcd.: 1131.8).

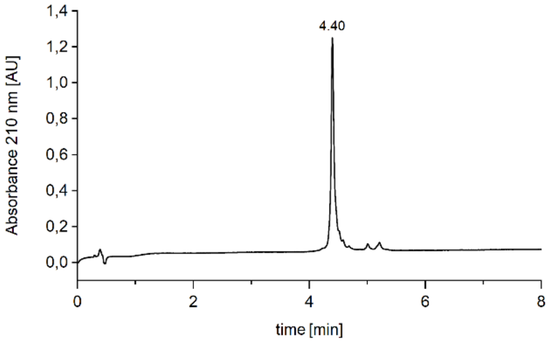

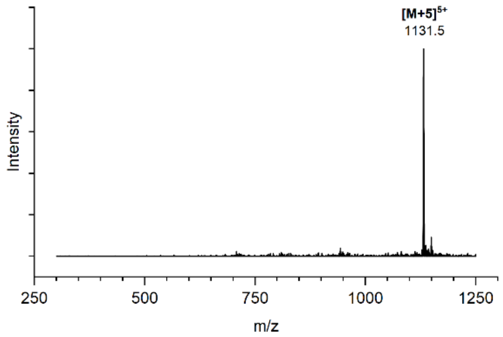

HHHHHH-PEG$_6$-YSPTSPS-YSPTSPS-YSPTSPS-YSPTSPS-YSPTSPS-Y**pS**PT**pS**PS-NH$_2$, **70**

Yield: 2.08 µmol, 42%.
MW: 5653.7 g·mol^{-1} ($C_{243}H_{346}N_{62}O_{91}P_2$).
UPLC-MS: t_R = 4.40 min (3 → 40 % B1 in 8 min); m/z = 1131.8 ($C_{243}H_{351}N_{62}O_{91}P_2$ $(M+5H)^{5+}$, calcd.: 1131.8).

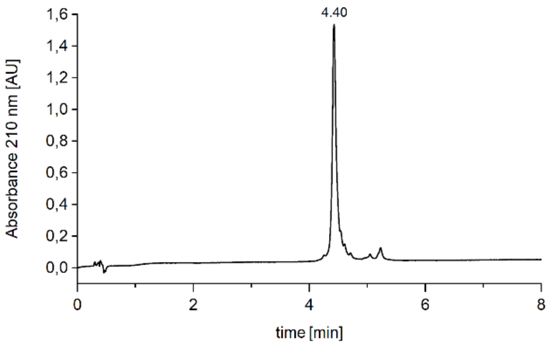

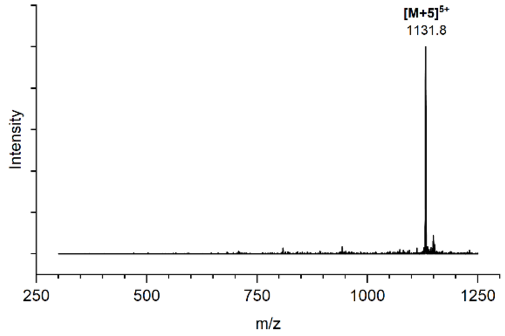

HHHHHH-PEG$_6$-YSPTSPS-YSPTSPS-YSPTSPS-YSPTSPS-YSPTSPS-Y**pS**PTSP**pS**-NH$_2$, **71**

Yield: 1.19 µmol, 24%.
MW: 5653.7 g·mol^{-1} ($C_{243}H_{346}N_{62}O_{91}P_2$).
UPLC-MS: t_R = 4.41 min (3 → 40 % B1 in 8 min); m/z = 1131.8 ($C_{243}H_{351}N_{62}O_{91}P_2$ $(M+5H)^{5+}$, calcd.: 1131.8).

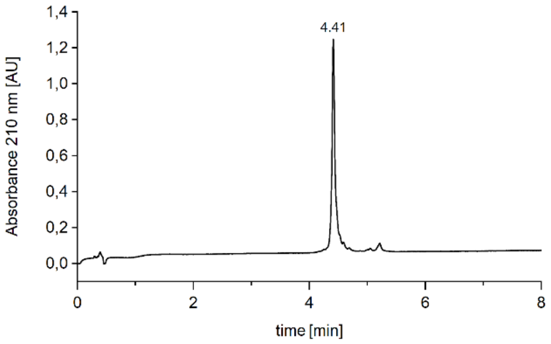

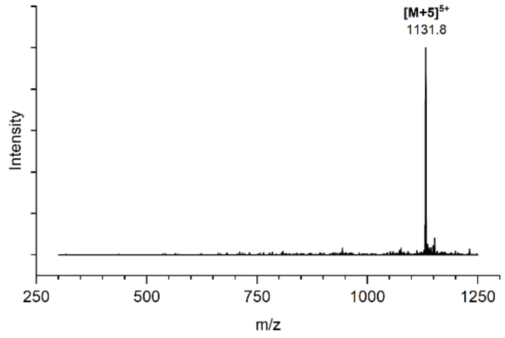

HHHHHH-PEG$_6$-YSPTSPS-YSPTSPS-YSPTSPS-YSPTSPS-YSPTSP**pS**-Y**pS**P**pT**SPS-NH$_2$, **72**

Yield: 1.85 µmol, 37%.
MW: 5733.7 g·mol^{-1} ($C_{243}H_{347}N_{62}O_{94}P_3$).
UPLC-MS: t_R = 4.44 min (3 → 40 % B1 in 8 min); m/z = 1147.5 ($C_{243}H_{352}N_{62}O_{94}P_3$ (M+5H)$^{5+}$, calcd.: 1147.7).

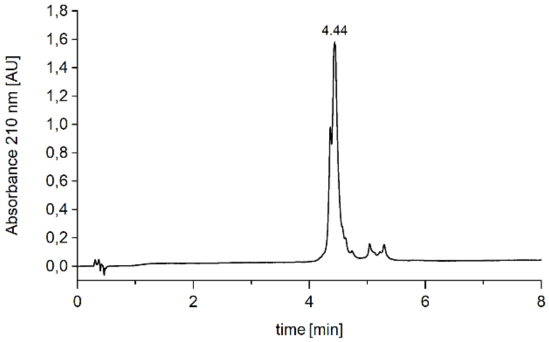

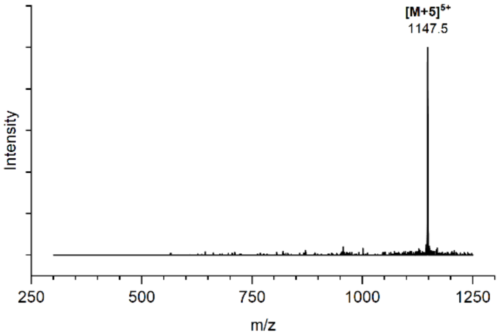

HHHHHH- PEG$_6$--YSPTSPS-YSPTSPS-YSPTSP**pS**-YSPTSPS-YSPTSPS-YSPTSPS-NH$_2$, **77**

Yield: 4.26 µmol, 36%.
MW: 5573.8 g·mol^{-1} ($C_{243}H_{345}N_{62}O_{88}P$).
UPLC-MS: t_R = 4.26 min (3 → 40 % B1 in 8 min); m/z = 1115.3 ($C_{243}H_{350}N_{62}O_{88}P$ (M+5H)$^{5+}$, calcd.: 1115.8).

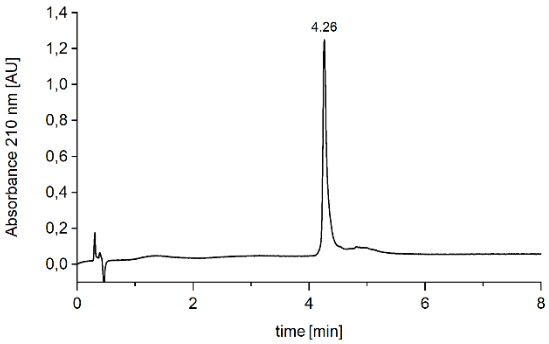

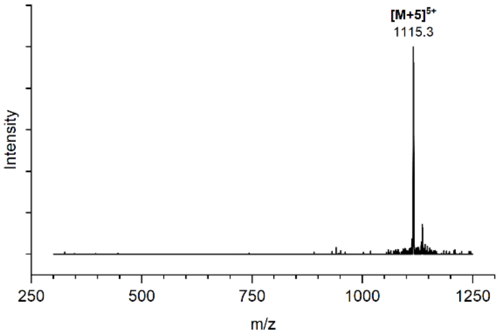

HHHHHH-PEG$_6$-YSPTSPS-YSPTSPS-YSPTSPS-YSPTSPS-YSPTSPS-YSPTSP**pS**-NH$_2$, **78**

Yield: 1.30 µmol, 26%.
MW: 5573.8 g·mol^{-1} ($C_{243}H_{345}N_{62}O_{88}P$).
UPLC-MS: t_R = 4.37 min (3 → 40 % B1 in 8 min); m/z = 1115.5 ($C_{243}H_{350}N_{62}O_{88}P$ (M+5H)$^{5+}$, calcd.: 1115.8).

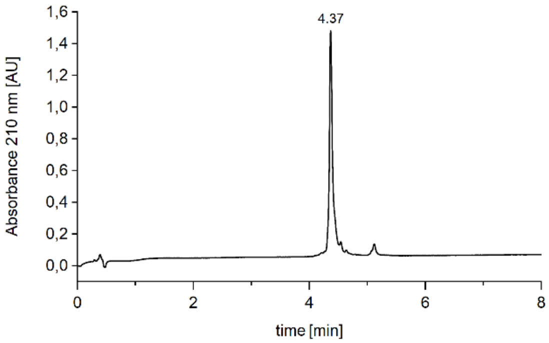

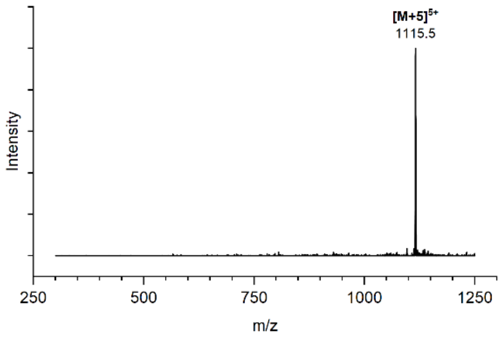

HHHHHH-PEG$_6$-YSPTSPS-YSPTSPS-YSPTSPS-Y**pS**ATSPS-YSPTSPS-YSPTSPS-NH$_2$, **79**

Yield: 1.74µmol, 35%.
MW: 5547.7 g·mol^{-1} ($C_{241}H_{343}N_{62}O_{88}P$).
UPLC-MS: t_R = 4.37 min (3 → 40 % B1 in 8 min); m/z = 1110.3 ($C_{241}H_{348}N_{62}O_{88}P$ (M+5H)$^{5+}$, calcd.: 1110.5).

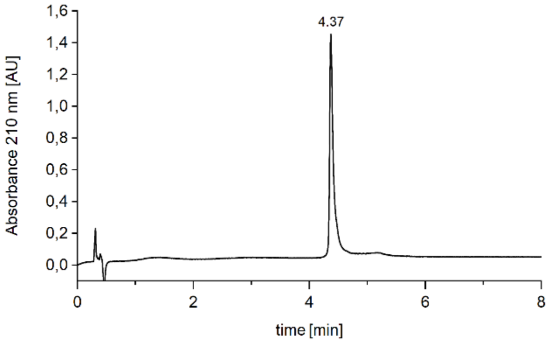

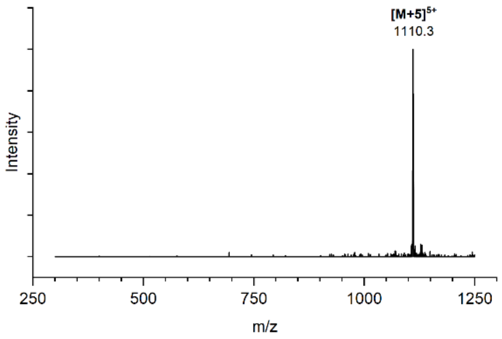

HHHHHH-PEG$_6$-YSPTSPS-YSPTSPS-YSPTSPS- Y**pS**<u>**A**</u>**pT**SPS-YSPTSPS-YSPTSPS-NH$_2$, **80**

Yield: 1.65 µmol, 33%.
MW: 5626.4 g·mol^{-1} ($C_{241}H_{344}N_{62}O_{91}P_2$).
UPLC-MS: t_R = 4.42 min (3 → 40 % B1 in 8 min); m/z = 1126.1 ($C_{241}H_{349}N_{62}O_{91}P_2$ (M+5H)$^{5+}$, calcd.: 1126.3).

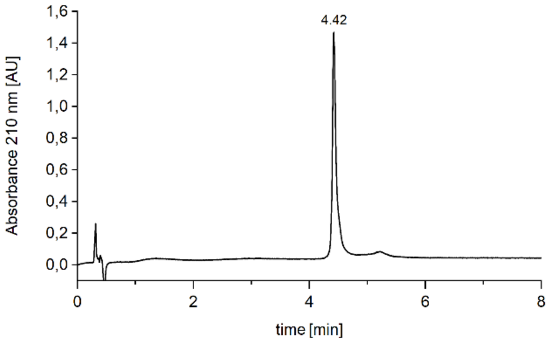

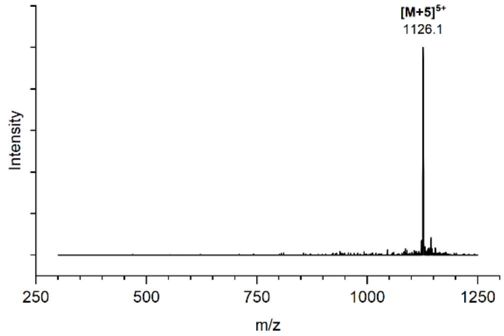

7. References

(1) Harris, P. W. R.; Williams, G. M.; Shepherd, P.; Brimble, M. A. The Synthesis of Phosphopeptides Using Microwave-assisted Solid Phase Peptide Synthesis. *Int. J. Pept. Res. Ther.* **2008**, *14* (4), 387-392.
(2) Boeglin, D.; Lubell, W. D. Aza-Amino Acid Scanning of Secondary Structure Suited for Solid-Phase Peptide Synthesis with Fmoc Chemistry and Aza-Amino Acids with Heteroatomic Side Chains. *J. Comb. Chem.* **2005**, *7* (6), 864-878.
(3) Bird, M. J.; Dawson, P. E. A shelf stable Fmoc hydrazine resin for the synthesis of peptide hydrazides. *Pept. Sci.* **2022**, 114 (5), e24268
(4) Martin, V.; Jadhav, S.; Egelund, P. H. G.; Liffert, R.; Johansson Castro, H.; Krüger, T.; Haselmann, K. F.; Thordal Le Quement, S.; Albericio, F.; Dettner, F.; et al. Harnessing polarity and viscosity to identify green binary solvent mixtures as viable alternatives to DMF in solid-phase peptide synthesis. *Green Chem.* **2021**, *23* (9), 3295-3311.
(5) Micsonai, A.; Moussong, É.; Wien, F.; Boros, E.; Vadászi, H.; Murvai, N.; Lee, Y.-H.; Molnár, T.; Réfrégiers, M.; Goto, Y.; et al. BeStSel: webserver for secondary structure and fold prediction for protein CD spectroscopy. *Nucleic Acids Res.* **2022**, *50* (W1), W90-W98.
